# Harnessing Interactional Sensory Genes for Rationally Reprogramming Chaotic Metabolism

**DOI:** 10.34133/research.0017

**Published:** 2022-12-21

**Authors:** Chunlin Tan, Ping Xu, Fei Tao

**Affiliations:** State Key Laboratory of Microbial Metabolism, Joint International Research Laboratory of Metabolic and Developmental Sciences, and School of Life Sciences and Biotechnology, Shanghai Jiao Tong University, Shanghai, China.

## Abstract

Rationally controlling cellular metabolism is of great importance but challenging owing to its highly complex and chaotic nature. Natural existing sensory proteins like histidine kinases (HKs) are understood as “sensitive nodes” of biological networks that can trigger disruptive metabolic reprogramming (MRP) upon perceiving environmental fluctuation. Here, the “sensitive node” genes were adopted to devise a global MRP platform consisting of a CRISPR interference-mediated dual-gene combinational knockdown toolbox and survivorship-based metabolic interaction decoding algorithm. The platform allows users to decode the interfering effects of *n* × *n* gene pairs while only requiring the synthesis of *n* pairs of primers. A total of 35 HK genes and 24 glycine metabolic genes were selected as the targets to determine the effectiveness of our platform in a *Vibrio* sp. FA2. The platform was applied to decode the interfering impact of HKs on antibiotic resistance in strain FA2. A pattern of combined knockdown of HK genes (*sasA_8* and *04288*) was demonstrated to be capable of reducing antibiotic resistance of *Vibrio* by 108-fold. Patterns of combined knockdown of glycine pathway genes (e.g., *gcvT* and *ltaE*) and several HK genes (e.g., *cpxA* and *btsS*) were also revealed to increase glycine production. Our platform may enable an efficient and rational approach for global MRP based on the elucidation of high-order gene interactions. A web-based 1-stop service (https://smrp.sjtu.edu.cn) is also provided to simplify the implementation of this smart strategy in a broad range of cells.

## Introduction

Metabolism has long been the center of our investigation and utilization of biological systems. Metabolic reprogramming (MRP) is recognized as involved in physiology, such as drug resistance, cell differentiation, cancer progression, and metastasis [1,2]. For example, antibiotic resistance has been repeatedly reported to result from spontaneous MRP [3–5]. MRP is also a fundamental approach in synthetic biology that involves the redirection of metabolic flux and remodeling of metabolic networks [6]. For example, yeast cell growth on xylose was substantially enhanced by performing a global rewiring of the cellular metabolic network [7]. Wang et al. [8] discovered a switch of metabolic status that can reshape the cellular metabolic network, resulting in high-level acetoin production. Therefore, MRP is a crucial process for controlling and understanding cells in physiology and biomanufacturing.

Unfortunately, it is difficult to artificially achieve an effective MRP, especially at the global level of metabolic networks. Theoretically, the primary obstacle lies within the complexity of the chaotic metabolic networks, which consist of thousands of metabolites, enzymes, genes, and their interactions [9]. As this complexity results in poor predictability of metabolic behavior, it is difficult to predict the final cellular response to a given metabolic perturbation [10]. Practically, the lack of an effective approach for decoding the combinational metabolism interference severely increases the space of trials in MRP studies, depriving the advanced opportunity for MRP research [11]. Currently, it is still time-consuming and labor-intensive to obtain the desired production performance in biomanufacturing [12]. For instance, it took more than 10 years of hard work and around 50 million US dollars for Paddon et al. [13] to industrialize the biosynthesis process of artemisinin and achieve large-scale mass production of artemisinic acid. There is an urgent need to develop powerful tools for addressing these challenges.

**Fig. 1.**
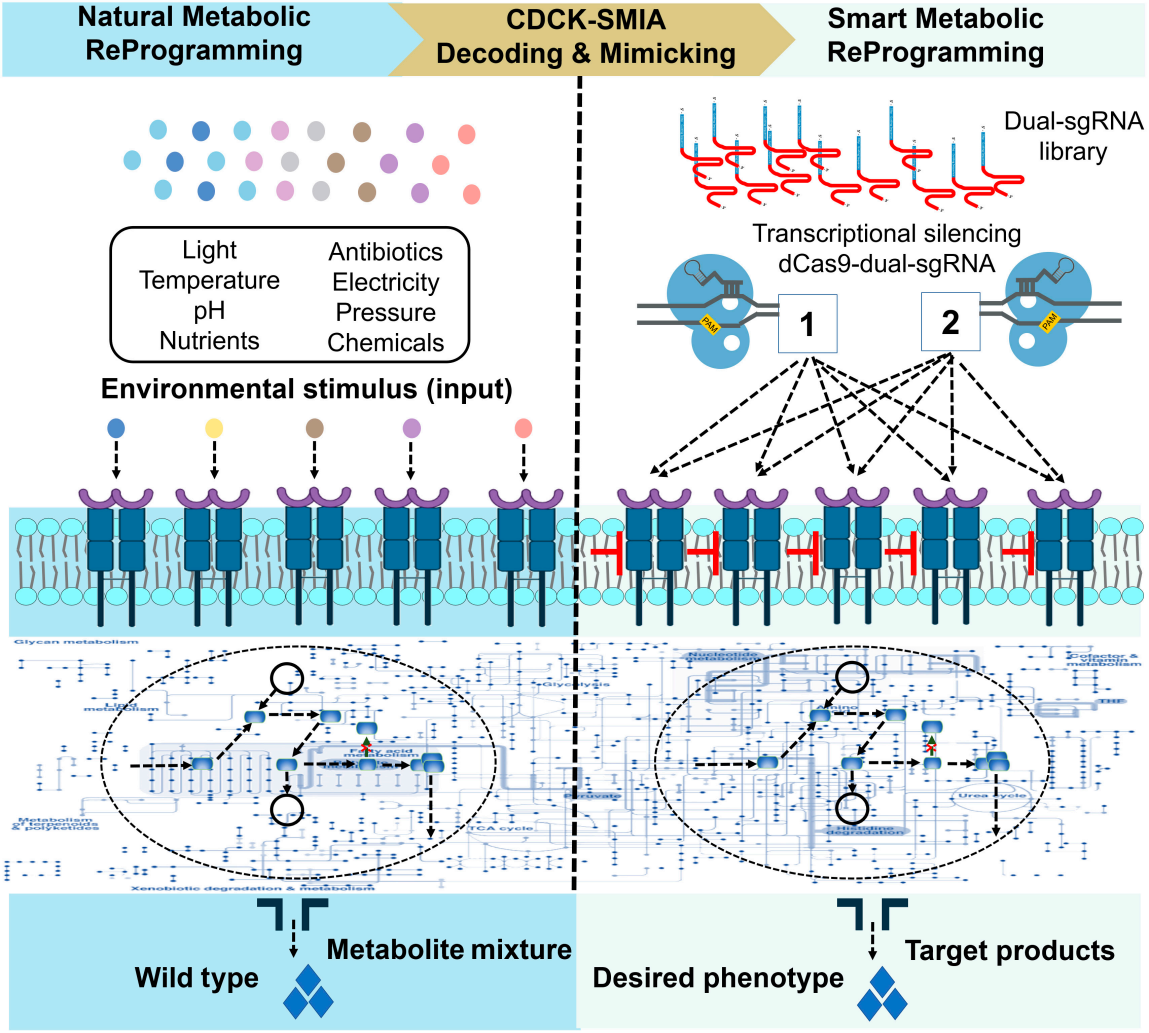
Schematic diagram of smart metabolic reprogramming. The different environmental cues, such as light, temperature, chemicals, pH, and/or pressure, naturally stimulate microbial cells to switch on their cell metabolic reprogramming (MRP) in response to these changes. The sensory proteins, such as histidine kinases, can be conceptualized as sitting on “sensitive nodes” of the metabolic networks; these can trigger natural MRP through high-order gene interactions upon perceiving environmental fluctuation. In the present study, we developed a platform for decoding and mimicking the natural MRP caused by environmental stimulations based on combinational interference of “sensitive node” genes. This method is exceptionally effective in changing metabolic status, which can be considered smart MRP in terms of the high interaction order, convenience, and throughput. CDCK, the CRISPRi-mediated dual-gene combinational knockdown; SMIA, survivorship-based metabolic interaction analysis algorithm.

In contrast, various environmental cues, such as light, temperature, chemicals, and pH, can naturally stimulate cells to switch on MRP [14]. This natural MRP is achieved by sensing environmental signals through natural sensory proteins **(**Fig. 1**)**. Natural existing sensory proteins, such as histidine kinases (HKs), are understood as “sensitive nodes” of the metabolic networks, which can perceive and transduce environmental signals to cellular responses, including MRP in many physiological processes such as cell development, antibiotic resistance, and chemotaxis [14]. HKs are well known as the sensory proteins that control the expression of genes for adaptation to the changing environments. Certain HKs have been shown to contribute to antibiotic resistance. For example, a sensory protein of HKs was recognized as a β-lactam receptor to induce resistance to β-lactam antibiotics. Li et al. [15] identified a previously unidentified mechanism that HK/response regulator pair (*VbrK*/*VbrR*) in *Vibrio parahaemolyticus* triggers the expression of a β-lactamase and the resistance of the strains to β-lactam antibiotics. In addition, transcription factors can switch cellular metabolic status by interacting with small molecules, ions, environmental factors (e.g., temperature or pH), amino acids, succinate, and secondary metabolites [16–19]. Commonly, natural MRP caused by natural sensory proteins is substantial and effective owing to their importance for the survival of cells in fluctuating environments [20]. Therefore, mimicking natural MRP caused by environmental vibrations via “sensitive node” gene manipulation could be an effective strategy for reprogramming microbial metabolic networks.

In this study, we developed a platform that can decode the interfering effects of different sensory genes in genome-scale and rational reprogramming cellular global metabolism (Figs. 1 and 3). This platform consists of a CRISPR interference (CRISPRi)-mediated dual-gene combinational knockdown (CDCK) toolbox and survivorship-based metabolic interaction analysis (SMIA) algorism. Using this platform, we successfully decoded the interfering MRP effects on bacterial antibiotic resistance by targeting all HKs of the *Vibrio alginolyticus* FA2, reducing antibiotic tolerance by 108 folds. *V. alginolyticus* FA2 is a fast-growing bacterial chassis that is considered as the next-generation biotechnological workhorse [21]. *V. alginolyticus* FA2 has characteristics of fast growth, fast substrate assimilation, protein synthesis, and biomass production, thus increasing attraction in biomanufacturing. Several *Vibrio* strains such as *V. parahaemolyticus* are important pathogens, which attracts researchers' interests for physiology. Therefore, we chose the *V. alginolyticus* FA2 to test our MRP platform both in physiology and biomanufacturing. In addition, we investigated the effects of MRP targeting HKs and glycine pathway genes on the production of amino acids. To make the platform more available, we established a website for MRP research, in which users can access our 1-stop service of automatically designing related experiments and conducting data analysis by simply uploading raw data. Overall, our platform is versatile and can be considered a smart MRP platform that can be widely adopted to decode and control a broad range of metabolic networks rationally.

## Results

### Testing of CRISPRi-mediated multiple gene knockdown in *V. alginolyticus* FA2

**Fig. 2.**
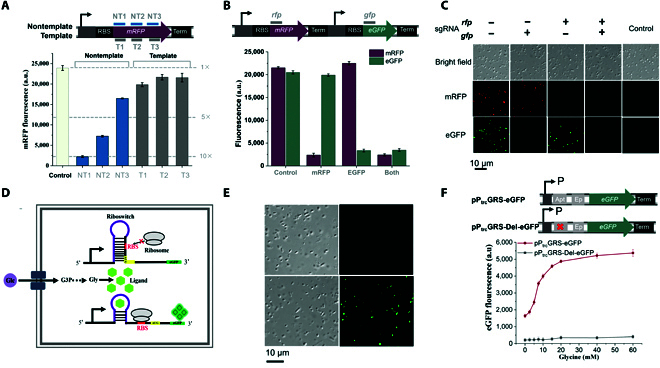
Performance of CRISPRi-based multiple gene knockdown and glycine-on riboswitch in the fast-growing *Vibrio* chassis. (A) CRISPRi blocks transcription elongation in a strand-specific manner. A synthetic fluorescence-based reporter system containing the mRFP-coding gene and eGFP-coding gene is used here to test the CRISPRi system. Six sgRNAs that bind to the nontemplate (NT) DNA strand and template (T) DNA strand are expressed from the pE2a plasmid, with their effects targeting mRFP and eGFP measured by in vivo fluorescence assays. The control shows fluorescence of the cells with the dCas9 protein but without sgRNA. (B) Multiple sgRNAs can independently silence mRFP and eGFP fluorescence protein reporters in the same cell. Each sgRNA specifically silences its cognate gene but not the other gene. Both the mRFP and eGFP-coding genes are repressed when both the sgRNAs are present. (C) Microscopic images for using 2 sgRNAs to control the fluorescence protein expression. (Top) Bright-field images of the *Vibrio alginolyticus* FA2 cells; (middle) RFP channel; (bottom) GFP channel. Scale bar, 10 μm. Control: cells without fluorescence reporters. (D) Schematic diagram of the glycine-on riboswitch principle. In the glycine biosensor, eGFP can serve as a reporter signal so that the concentration of glycine is coupled to the fluorescence intensity. (E) Fluorescence microscopy images of *V. alginolyticus* FA2 strain carrying the glycine-on riboswitch with (lower row) or without (upper row) addition of glycine. (F) Fluorescence values for 2 different constructs. eGFP values were measured when cultivated with varying concentrations of glycine. Values of eGFP and mRFP are median ± SD for at least 3 biological replicates. P_trc_: promoter trc; Apt: aptamer; Ep: expression platform. GRS: glycine riboswitch. pP_trc_GRS-eGFP: glycine riboswitch controlled by P_trc_. pP_trc_GRS-Del-eGFP: glycine aptamers were deleted.

As shown in Fig. 1, we wanted to develop a platform for global MRP by mimicking environmental stimulation based on combinational knocking down natural sensory genes using CRISPRi. To test the functionality of the CRISPRi system in the *V. alginolyticus* FA2, we created a reporter *V. alginolyticus* FA2 expressing mRFP (monomeric red fluorescent protein [22]), a catalytically dead Cas9 (dCas9 [23]), and sgRNAs complementary to 1 of 2 mRFP coding sequence regions [23]: the template (T) or nontemplate (NT) DNA strand (Fig. 2A). Our results indicated that single-guide RNAs (sgRNAs) targeting the nontemplate DNA strand effectively repressed *mrfp* gene expression (>10-fold repression), whereas those targeting the template strand showed little effect (Fig. 2A).

We next assessed whether the CRISPRi system could knock down multiple genes independently in the *V. alginolyticus* FA2. Briefly, a dual-color fluorescence system based on mRFP and eGFP (enhanced green fluorescent protein [22]) was devised, and 2 sgRNAs complementary to the NT1 sites of the mRFP and the eGFP coding sequences were designed (Fig. 2B and C). The expression of each sgRNA alone only silenced its cognate gene, exerting no impact on the other genes. Upon coexpression of the 2 sgRNAs, both genes *mrfp* and *egfp* were successfully knocked down (Fig. 2B and C). These results support that the CRISPRi system works well and show that sgRNA-guided targeting is specific and not affected by other sgRNAs in the *V. alginolyticus* FA2.

### Testing of *Vibrio* riboswitch for high-throughput screening

Riboswitch-mediated sensors can be powerful tools for timely and precise monitoring of the production of analyte/target compounds during fermentation [24]. A glycine-on riboswitch, which was from the upstream nucleotide sequences of *VC1422* gene of *Vibrio cholerae* [25], was used to detect the glycine concentrations in the cell and subsequently combined with fluorescence-activated cell sorting (FACS) to develop a high-throughput screening approach. A schematic of the principle of the glycine-on riboswitch is shown in Fig. 2D. The glycine-on riboswitch can change its secondary structure and activate the expression of its downstream *egfp* gene in response to an increased amount of glycine. Given the critical role of the leading promoter, we used different promoters to construct test plasmids (pP_trc_GRS-eGFP, pP_J23100_GRS-eGFP, and pP_native_GRS-eGFP) and corresponding control plasmids, in which glycine aptamers were deleted (pP_trc_GRS-Del-eGFP, pP_J23100_GRS-Del-eGFP, and pP_native_GRS-Del-eGFP). The sequences of these plasmids are listed in Table S3.

To determine whether the glycine-on riboswitch could respond to increased glycine concentrations, constructs and their corresponding control plasmids were transferred into the *V. alginolyticus* FA2. To select an appropriate glycine concentration range, we profiled the growth of the *V. alginolyticus* FA2 in a chemically defined M9 medium [26] supplemented with various concentrations (10 to 100 mM) of glycine. Cell growth was significantly inhibited in the M9 medium supplemented with 70 to 100 mM glycine (Fig. S4). Therefore, we tested the functionality of the glycine-riboswitch in the *V. alginolyticus* FA2 using an M9 medium supplemented with various concentrations (0 to 60 mM) of glycine. The dose response of the glycine-on riboswitch-based eGFP sensors is shown in Fig. 2E and F. Our findings suggest that the pP_trc_GRS-eGFP-containing strain can upregulate eGFP expression in response to an increased glycine concentration; no activation was observed in its control (pP_trc_GRS-Del-eGFP-containing strain). The average eGFP fluorescence value increased (>3-fold) when the glycine concentration was increased from 0 to 60 mM (Fig. 2F). The glycine-on riboswitch responds approximately linearly to glycine in the concentration range of 0–20 mM (Fig. 2F). Our results indicate that the glycine-on riboswitch functions well and provides the basis for high-throughput screening in the *V. alginolyticus* FA2.

### Establishment of MRP platform

**Fig. 3.**
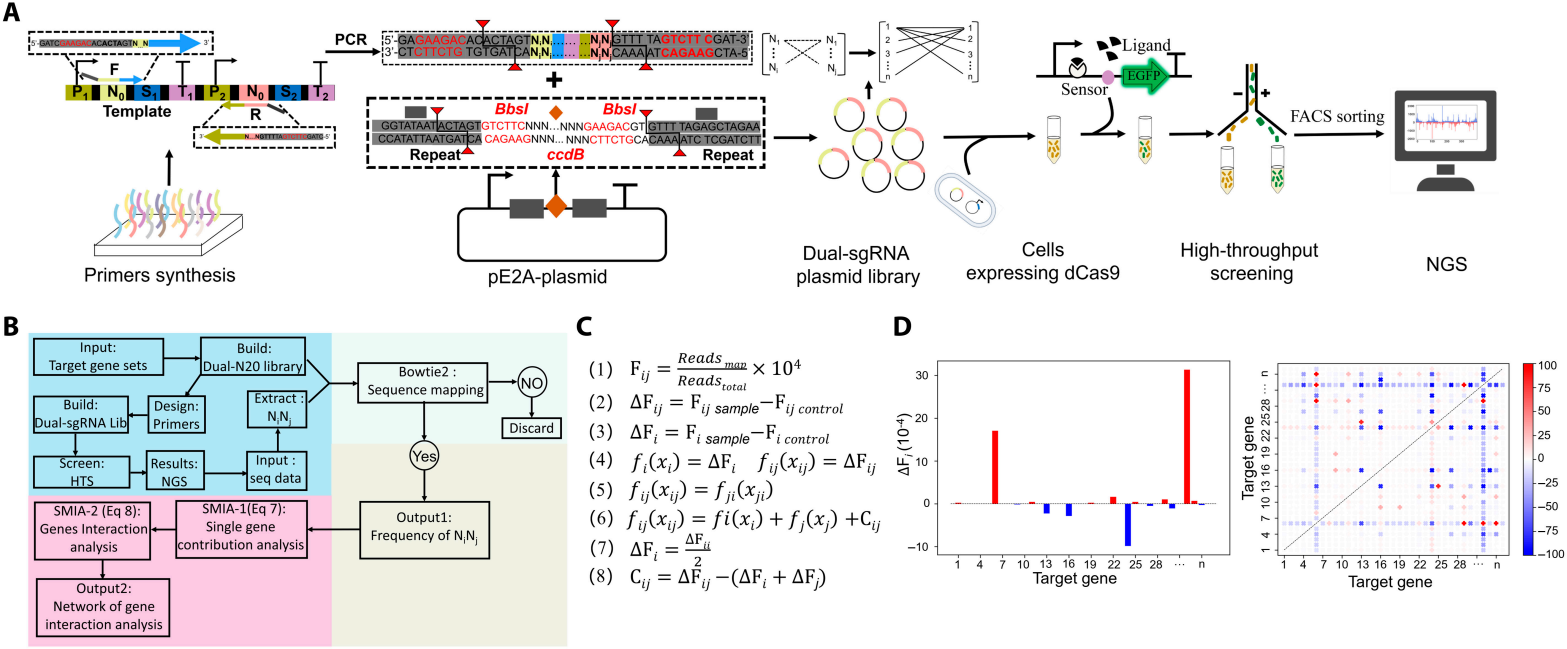
Principle and workflow of CDCK-SMIA platform. (A) Schematic representation of the CRISPRi-mediated dual-gene combinational knockdown (CDCK) coupled with biosensor-mediated high-throughput screening. A dual-sgRNA library targeting genes of interest is constructed by gene shuffling using polymerase chain reaction (PCR) and Golden Gate Assembly. The dual-sgRNA library gene segments are amplified from the template and cloned into the pE2a-tool plasmid by Golden Gate Assembly and then transformed into *E. coli*, resulting in dual-sgRNA library plasmid-producing strains. The dual-sgRNA plasmid is also transferred into the *Vibrio alginolyticus* FA2 expressing the dCas9 protein and a glycine-on riboswitch mediated with eGFP protein, resulting in cell libraries. The cell libraries are grown under isopropyl-β-d-thiogalactoside and arabinose to induce expression of the eGFP protein and the dCas9 protein, respectively. The induced cell libraries are assessed with FACS, and cells displaying higher fluorescence signals than control cells are sorted out. The plasmids of the sorted cells are extracted as the DNA template for subsequent PCR. Then, the dual-sgRNA segments are amplified by PCR from the template and are assessed by next-generation sequencing (NGS). (B) Flow diagram of the CDCK experiment design and survivorship-based metabolic interaction analysis (SMIA) for the smart metabolic reprogramming (MRP) platform. The workflow was embedded in the website SMRP (https://smrp.sjtu.edu.cn) to provide a 1-stop service for smart MRP research, which allows automatically designing of related experiments and comprehensive analysis of sequencing data. (C) Calculation equations of the SMIA platform. Defined i/or j, and C as the gene number and cross-index. In theory, the position effects of the i and j gene are the same, as shown in Eq. 5. Equation 7: *C*_ij_ = 0, i = j. Equation 8: *C*_ij_ ≠ 0, i ≠ j. (D) Representative results of the SMIA. (Left) Contribution of the single-gene knockdown. (Right) Contribution of the combinational dual-gene knockdown. (If *C_ij_* > 0, it indicates that i and j genes have a positive interaction, showing “+.” If *C_ij_* = 0, then the i and j genes are not interacting with each other, showing in white. If *C_ij_* < 0, then the i and j genes interact negatively, displaying as “×.”) The ∆*F_i_* indicates the contribution of single-gene knockdown to the survival frequency of a dual-gene knockdown pattern.

Our MRP platform consists of CDCK tools and a SMIA algorism. Fig. 3A presents a schematic diagram of our CDCK working principle. Briefly, the dual-gene knockdown library was obtained by randomly shuffling the DNA fragments containing different sgRNAs, with the effectiveness of generating n × n dual-gene knocking-down patterns while only requiring the synthesis of n pairs of primers. Libraries constructed using these dual-sgRNA patterns are exposed to subsequent selection pressure or FACS and then analyzed using high-throughput amplicon sequencing.

We constructed 2 dual-sgRNA libraries targeting 35 × 35 dual HKs and 24 × 24 dual glycine-pathway genes. Each dual-sgRNA cell library contains approximately 40,000 transformants in *E. coli* through polymerase chain reaction (PCR) shuffling and Golden-Gate Assembly. As shown in Fig. S2, each dual-sgRNA library in *E. coli* was termed library 1, such as HK-L1 for HK genes or G-L1 for glycine pathway genes. The dual-sgRNA library plasmids of the HK-L1 and G-L1 were then transferred by electroporation into the *V. alginolyticus* FA2 containing dCas9 and pP_trc_GRS-eGFP expression cassettes resulting in Library 2 (HK-L2 or G-L2). Generally, the observed frequencies of each sgRNA in L1 are relatively even, presenting less gene bias in library construction (Figs. 6F and 7F). The L2 library-containing strains were then cultivated with arabinose and isopropyl-β-d-thiogalactoside (IPTG) to switch on the CRISPRi system and eGFP expression, respectively. These library-containing cells are exposed to FACS or stress selection pressure.

To decode the cross-talk of different genes, we developed the SMIA algorism, which can be used for determining both single-gene and combined-gene contributions to physiological responses directly from high-throughput sequencing data and calculating the interaction parameters of different genes accordingly. As shown in Fig. 3B, we first use Bowtie2 to map the obtained reads with the designed dual-gene knockdown library, count the number of times that each dual-gene knockdown pattern can be mapped, divide it by the total number of reads, and normalize it as the survival frequency of the dual-gene knockdown pattern (Fig. 3C and Eq. 1). As shown in Fig. 3C, we characterized gene interactions by comparing changes in survival frequency between control and test samples. The single-gene contribution to the metabolic response upon selection pressure was first calculated by the survival-frequency change of the knockdown patterns with 2 sgRNAs targeting the same gene (Eq. 7). Then, gene interaction extent (cross-index) was determined by subtracting the sum of 2 single-gene-contributed survival-frequency changes from the survival-frequency change of corresponding dual-gene knockdown patterns (Eq. 8). The representative results of SMIA are shown in Fig. 3D.

Our MRP platform consists of CDCK approach and SMIA algorism. For the CDCK approach, we developed the dual-sgRNA cell library in *E. coli* through PCR shuffling and Golden-Gate Assembly. The dual-sgRNA library plasmids were then transferred by electroporation into the target strain. We could reasonably anticipate that the platform is feasible in the target cells, as long as the CRISPRi tools function well in the target cells. In addition, we successfully conducted the bioinformatic design for sensitive nodes, HKs, and regulators through our web-based MRP platform in a further 13 frequently used microorganisms, as described in Table S28. We put the well-designed primers in the supplementary bioinformatic design tables; therefore, the researchers can directly conduct the related experiments. Furthermore, our web-based 1-stop service can simplify the implementation of the SMIA in this smart strategy, thus increasing the feasibility of our platform. On the basis of the bioinformatics analysis, it would be feasible to conduct rational designs for a broad range of cells; we, therefore, anticipate that our platform should apply to a wide variety of species.

To facilitate the experimental design of CDCK and subsequent SMIA, we established a website to provide a 1-stop service (https://smrp.sjtu.edu.cn). Using this website, one can design a dual-gRNA library with a customized target gene set or let the server automatically extract the gene set and create the library by simply uploading a genome sequence in FASTA format. It takes 15 to 45 min for a typical microbial genome, depending on the number of sensitive-node genes that can be annotated. After completing the high-throughput sequencing, one can get the SMIA job done by uploading the sequencing data and obtaining a comprehensive analysis report (supplementary SMIA analysis report). Typically, the analysis service takes 5 to 30 min, depending on the data size and the number of target genes. We provide an account register service to help users to manage tasks conveniently. Anyone can submit tasks through the registered account, check the task status and download data with a web browser.

### Interfering effects of MRP targeting HKs on antibiotic resistance

**Fig. 4.**
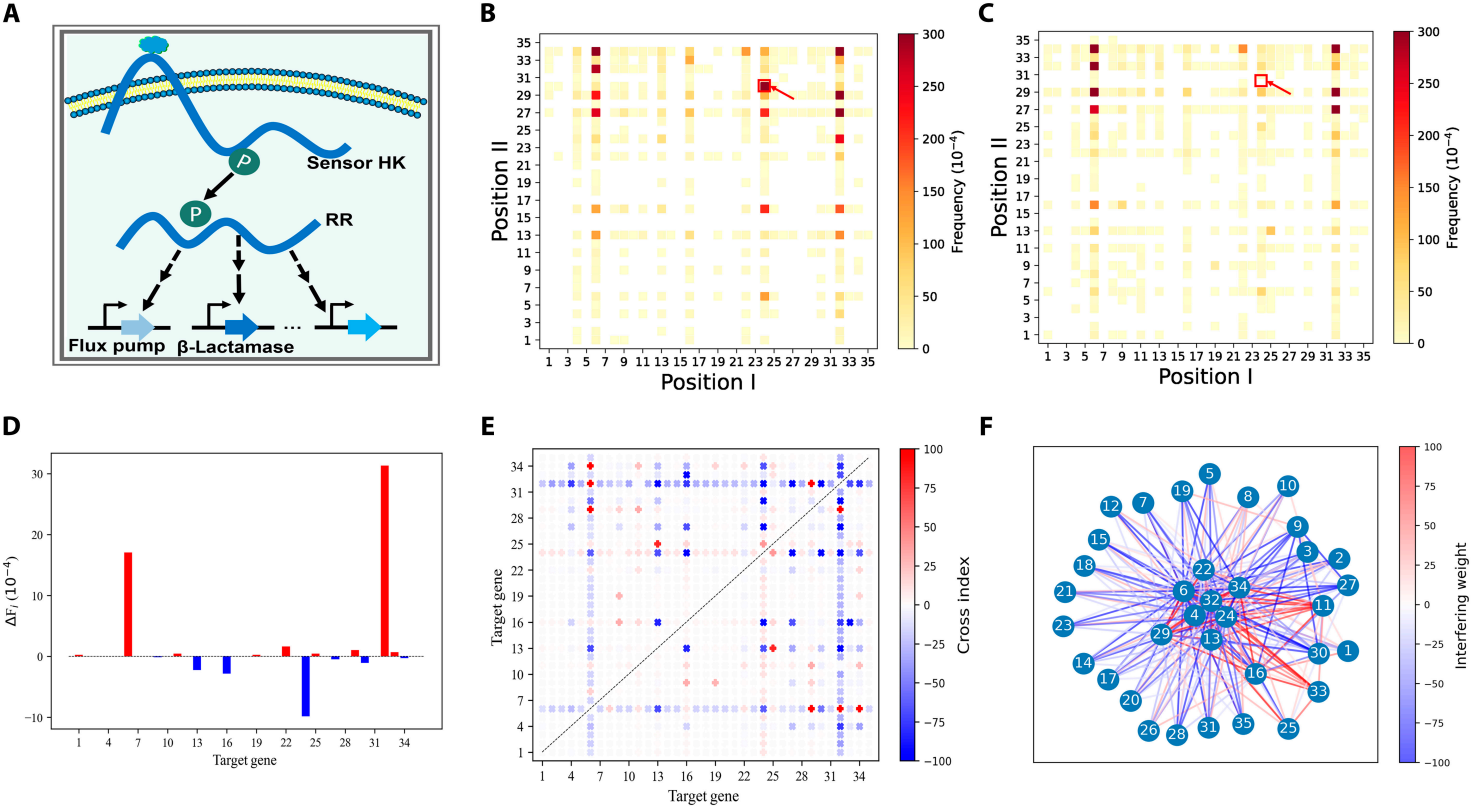
Interfering effects of metabolic reprogramming (MRP) targeting histidine kinases (HKs) on the antibiotic resistance of *Vibrio alginolyticus* FA2. (A) Schematic model for HK-mediated signal transduction that affects antibiotic resistance through high-order gene interaction. HKs are known as the sensory proteins for sensing environmental signals, leading to the autophosphorylation and subsequent phosphoryl transfer to the cognate response regulators. Upon phosphorylation, a response regulator usually controls the expression of genes for adaptation to changing environments. Sensory proteins of HKs for instance have been recognized as β-lactam receptors to induce resistance to β-lactam antibiotics. (B) Frequency of the different dual-sgRNA cassettes of HK-L2 library in dCas9-containing *V. alginolyticus* FA2 cultivated without ampicillin (control) and (C) with 5 mg/ml of ampicillin (experiments). (D) Contribution of the single-gene knockdown to the survival-frequency change of the coculture. (E) Contribution of the combinational dual-gene knockdown for the survival-frequency change of the coculture. (F) Gene-to-gene interaction networks analysis. The connection of different nodes indicates that there are interaction effects between 2 genes. When the edge gets thicker, the interaction effects are more substantial. Red represents the 2 genes' positive interaction, and blue indicates that the 2 genes interact negatively. All the HK genes of the *V. alginolyticus* FA2 are named and numbered from 1 to 35. Interfering weight represents the superposition of the frequency change contributions observed in different experiments. (For interpretation of the references to color in this figure legend, the reader is referred to the web version of this article.)

**Fig. 5.**
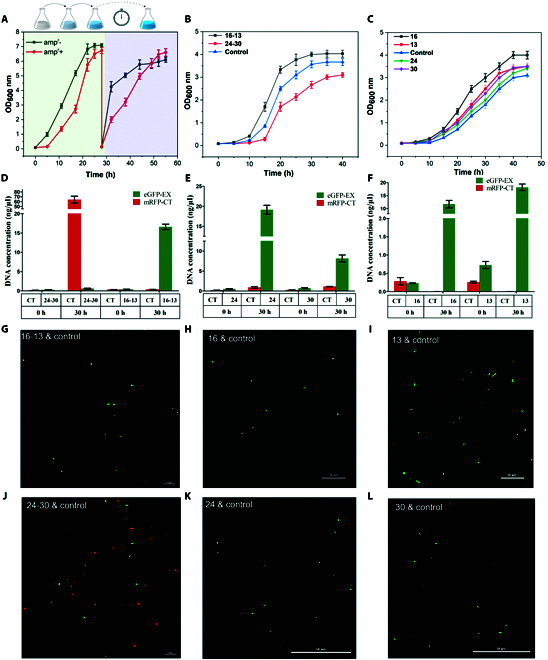
Experiment validation for the interfering effects of metabolic reprogramming (MRP) targeting histidine kinases (HKs) on the antibiotic resistance of the *Vibrio alginolyticus* FA2. (A) Comparisons of cell growth of strain HK-L2 in the LB3 medium cultivated with or without 5 mg/ml of ampicillin. The strains were grown at 37 °C, 200 rpm for 28 h, and the cells were transferred to the fresh LB3 medium and cultured for 28 h again with 5 mg/ml ampicillin. (B) Comparisons of cell growth of strain 24-30, strain 16-13, and strains without sgRNA libraries (control). (C) Comparisons of cell growth of strains 24, 30, 16, 13, and the control. (D to F) qPCR assessing the DNA concentration for the 2 marker genes (*mrfp* and *egfp*) in cocultures of strain 24-30 and control cells; strain 16-13 and control cells; strain 24 and control cells; strain 30 and control cells; strain 16 and control cells; as well as strain 13 and control cells, at the initial and late logarithmic growth stages. EX: experimental group; CT: control group; eGFP-EX: A synthetic eGFP-based reporter system was introduced into the strains with the corresponding sgRNA (experimental group); mRFP-CT: An artificial mRFP-based reporter system was introduced into the strains without sgRNA (control). (G to L) Microscopic images of the cocultured cells of strain 16-13 and control; strain 16 and control; strain 13 and control; 24-30 and control; strain 24 and control; strain 30 and control at the late logarithmic growth stages, respectively. Control: the dCas9-containing *V. alginolyticus* FA2 without the sgRNA library. Values of OD_600_ nm and DNA concentration assessed by qPCR are median ± SD for at least 3 biological replicates.

The CDCK-SMIA platform was used to investigate the effects of MRP on the resistance of the *V. alginolyticus* FA2 to the antibiotic ampicillin when targeting HKs. We selected 35 HK genes as targets for the combinational knockdown (Fig. 4). The details of the targeted genes (numbered 1 to 35), such as gene name and gene ID, are shown in Table S4. We also used the number of HKs to represent the strains, in which the corresponding HK genes were knocked down. Here, we cultivated the strain targeting HKs (HK-L2) in an lysogeny broth 3 (LB3) medium containing 5 mg/ml ampicillin. As shown in Fig. 5A, the antibiotics significantly inhibited the growth of the *V. alginolyticus* FA2. After 16 h of cultivation, the optical density at 600 nm (OD_600_) of strain HK-L2 (with ampicillin) decreased by approximately 2-fold compared to the control (without ampicillin); this was the largest detected difference in growth. Subsequently, the resulting strains were transferred to fresh LB3 medium as seeds and supplemented with 5 mg/ml ampicillin again for the second round of antibiotic exposure. After 33 h of cultivation, the OD_600_ of strain HK-L2 cultivated with or without ampicillin was approximately 1.8 and 4.0, respectively. A higher growth difference between the control and experimental group was obtained compared with that of the previous 28 h (Fig. 5A). Therefore, we harvested cells during this period for next-generation sequencing (NGS). Every round of the screening experiment was repeated 3 times, for instance, namely 2-CT-1, 2-CT-2, and 2-CT-3 for control groups and 2-EX-1, 2-EX-2, and 2-EX-3 for experimental groups as for the second-round screening.

The survival frequencies revealed significant differences between the control and experimental groups (Fig. 4B and C and Fig. S6). Specifically, the 3 dual-sgRNA cassettes with the most considerable frequency changes in the experimental group were 16-13, 13-29, and 6-33, compared to the control (experiment: control, per 10^4^ times, 272:18, 140:16, and 131:6, respectively). The frequency of dual-sgRNA cassettes of strain 24-30 was 648 in the 2-CT-3 group and 0 in the 2-Ex-3 group (Tables S22 and S25). Moreover, the dual-sgRNA cassettes of strain 24–30 were also remarkable in another independent experiment, in which the frequency (10^−4^) of strain 24–30 was 323 in the 2-CT-2 group and 0 in the 2-Ex-2 group (Tables S21 and S24). The results showed that ampicillin antibiotic pressure enriched some cells (e.g., strain 16–13), and some were washed out (e.g., strain 24–30). Through SMIA, we found that the genes HK-6, HK-22, HK-29, and HK-32 contribute positively to the survival of antibiotic resistance. In contrast, the genes HK-13, HK-16, HK-24, and HK-30 contribute negatively to survival (Fig. 4D and Fig. S11). According to the dual-gene combinational contribution analysis, many HK pairs in the *V. alginolyticus* FA2 were associated with antibiotic resistance, of which 5 pairs increase ampicillin resistance, and 18 pairs may reduce ampicillin resistance (Fig. 4E). To unveil the role of gene interactions on antibiotic resistance more thoroughly, we integrated the data generated from multiple antibiotic selection experiments to form a comprehensive interaction network (Fig. 4F). It is shown that every HK gene can affect drug resistance through interaction with other genes, of which HK-33 and HK-11 interacted with other genes to enhance resistance, while HK-30 interacted with other genes to decrease resistance. Surprisingly, some HK genes, such as HK-24, interact with some genes to increase resistance but interact with the other genes to decrease resistance (Fig. 4F).

For validation of the finds obtained in SMIA, we independently constructed strains targeting HKs, namely strains 16-13, 24-30, 16, 13, 24, and 30 (the numbers of the HKs were also defined as strains 16-13, 24-30, 16, 13, 24, and 30). Thereafter, the strains were cultured in an LB3 medium under the same cultivation condition as described above to determine whether they complied with the SMIA. As shown in Fig. 5B, the growth rate of the 16-13, 24-30, and control strains under the same condition during 0 to 15 h were 0.113, 0.018, and 0.057 h^−1^, respectively. In Fig. 5C, the growth rate of the 16, 13, 24, 30, and control strains were 0.100, 0.081 0.071 0.082, and 0.061 h^−1^, respectively. Thus, strains, in which HK-16 and HK-13 were simultaneously or individually knocked down, all grew approximately 1.3-fold to 2.0-fold faster than the control cells (wild-type strain expressed dcas9 without sgRNA) cultivated with 5 mg/ml ampicillin during 0 to 15 h (Fig. 5B and C). These results further validated that HK-24 and HK-30 were associated with ampicillin resistance in the *V. alginolyticus* FA2.

Surprisingly, the *V. alginolyticus* FA2, in which genes 24 and 30 were simultaneously knocked down, grew 3.1-fold slower than the control during 0 to 15 h (Fig. 5B). However, the *V. alginolyticus*s FA2, in which the genes HK-24 and HK-30 were individually knocked down, grew approximately 1.2 to 1.3-fold faster than the control during 0 to 30 h (Fig. 5C). These findings indicate that metabolic interaction, rather than independent effects of genes 24 and 30 may account for the observed decrease in *V. alginolyticus* FA2 tolerance to ampicillin antibiotics.

We expressed mRFP in the control strain and eGFP in strains 24-30, 16-13, 24, 30, 16, and 13 to further investigate the metabolic interactions under the condition of survival competition. Briefly, we cocultivated them under the same conditions as described above. After that, the plasmids were extracted from the cocultured cells at the initial and late logarithmic growth stages, and the DNA concentration of the 2 marker genes was quantified by quantitative PCR (qPCR). The different DNA concentrations of the marker genes represent the cell numbers of the control and experimental strains. As shown in Fig. 5D to F, the experimental and control DNA concentrations were almost equal at 0 h. At the late logarithmic growth stages of the coculture, the DNA concentration of the control was 63.9 ng/μl, which was approximately 108-fold higher than that of strain 24-30 (Fig. 5D). In contrast, the DNA concentrations of strains 24 and 30 were 19.1 ng/μl, and 8.2 ng/μl, respectively, which were approximately 21-fold and 7.1-fold higher than that of the respective control (Fig. 5E). These results support that the combinational knockdown of HK-24 and HK-30 conferred no growth disadvantage, while their independent knockdown conferred a growth advantage over the control when cocultured together. Therefore, it was further demonstrated that the metabolic interaction rather than independent effects of HK-24 and HK-30 leads to the *V. alginolyticus* FA2 decreasing its tolerance to ampicillin antibiotics.

At the late logarithmic growth stage of the coculture, the DNA concentration of strain 16-13 was approximately 45-fold higher than that of the control (Fig. 5D). The DNA concentrations of strains 16 and 13 were also higher than that of the control (Fig. 5F). These results support that both combinational and independent knockdown of HK-16 and HK-13 conferred a growth advantage over the control when they were cocultured together. It was indicated that the independent effects rather than metabolic interaction of HK-16 and HK-13 lead to the *V. alginolyticus* FA2 decreasing its tolerance to ampicillin antibiotics. To visually assess growth differences between the experimental and control strains, the cocultured bacteria were observed in the late logarithmic stage using a confocal microscope. After simultaneously activating the coculture cells by fluorescein isothiocyanate (FITC), tetramethylrhodamine (TRITC) channels of the confocal microscopy, the eGFP- and mRFP-containing cells were observed. As shown in Fig. 5G to L, the survival trends were consistent with those of qPCR and the growth curve analysis. This is the first reveal that interactions between HKs can affect antibiotic resistance, which suggests a strong potential of our MRP platform for studying the physiology of microorganisms, especially the effects of high-order interactions among multiple genes.

### Effects of MRP targeting of glycine pathway genes on glycine production

**Fig. 6.**
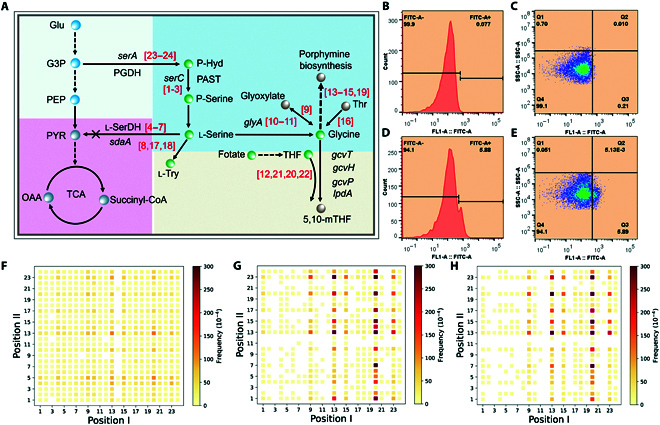
Effects of metabolic reprogramming (MRP) targeting glycine pathway genes on glycine production in *Vibrio alginolyticus* FA2. (A) Glycine metabolic pathway in *V. alginolyticus* FA2. All the glycine-related pathway genes are named and numbered from 1 to 24. (B and C) Flow cytometry diagrams showing representative distributions of the CRISPRi-mediated dual-gene combinational knockdown (CDCK) variant cells according to their green fluorescence for the cells without sgRNA library (control group). Q4/ or FITC-A-: cells below the threshold for the “Low” state; Q3/ or FITC-A+: cells in the “HIGH” state. (D and E) Flow cytometry diagrams showing representative distributions of the CDCK variant cells with sgRNA library (experiment group). (F) Frequency of the different dual-sgRNA cassettes of Gly-L1 library in wild-type *E. coli*. (G) Frequency of the different dual-sgRNA cassettes of Gly-L2 library in *V. alginolyticus* FA2 containing dCas9 before arabinose-based induction of dCas9 protein. (H) Frequency of the different dual-sgRNA cassettes of Gly-L2 library in dCas9-containing *V. alginolyticus* FA2 after induction. Abbreviations: Glu glucose, G3P glyceraldehyde3-phosphate, PEP phosphoenolpyruvate, Pyr pyruvate, OAA oxaloacetic acid, PGDH 3-phosphoglycerate dehydrogenase, PAST phosphoserine aminotransferase, PSP phosphoserine phosphatase, l-SerDH l-serine dehydratase, P-Hyd phosphohydroxypyruvate, TCA tricarboxylic acid cycle, THF tetrahydrofolate, 5,10-mTHF 5,10-methylenetetrahydrofol. (For interpretation of the references to color in this figure legend, the reader is referred to the web version of this article.)

To investigate the potential of our MRP platform for enhancing bioproduction, we also selected glycine synthesis as a target since it is one of the typical valuable amino acids. In addition, the glycine-riboswitch has been successfully demonstrated to activate the expression of its downstream *egfp* gene in response to an increased amount of glycine in the *V. alginolyticus* FA2 as described above. As a proof of concept, we constructed a compact sgRNA library targeting 24 glycine pathway genes to determine the effectiveness of our CDCK-SMIA platform in the fast-growing microbial chassis *V. alginolyticus* FA2, including those genes responsible for the glycine biosynthesis and degradation. The targeted genes were numbered 1 to 24, and the corresponding gene details are summarized in Fig. 6A and Table S5. We first constructed the dual-sgRNA library (G-L1) and then transferred the library into the *V. alginolyticus* FA2 containing dCas9-pP_trc_GRS-eGFP plasmid (G-L2). Variant cells from the CRISPRi-mediated dual-gene knockdown library of the glycine pathway were screened on the basis of their fluorescence using FACS. Cells with high fluorescence (G-S) were sorted compared to the control (Fig. 6B to E). Thereafter, the plasmids from these cells were extracted, and the dual-sgRNA cassette fragments were amplified using PCR.

We counted the frequencies of different dual-sgRNA cassettes in the sorted cells through NGS data analysis (Fig. 6F to H). The survival frequencies were significantly different among the L1, L2, and G-S groups. The survival frequencies of dual-sgRNA cassettes were relatively uniform in the L1 (Fig. 6F), while enrichments and decreases were observed in the L2 (Fig. 6G) and G-S (Fig. 6H) groups. This observation was attributed to the fact that the knockdown of core genes in the metabolic pathways could also negatively affect the growth of the cells. After careful analysis of the results from the G-S group, the dual-sgRNA cassette frequencies from the glycine degradation pathway were found to be markedly higher than those from the biosynthesis pathway (Fig. 6A and H). This result is consistent with the previous reports on glycine pathway engineering [27,28]. According to the analysis in Table S13, the dual-sgRNA cassette combination of genes HK-20 and HK-13 (20–13, *gcvT*-*ltaE*) showed the highest survival frequency (849 times/10^4^ times). It was indicated that the combined knockdown of the genes HK-20 and HK-13 would increase the in vivo glycine production. Further, we found that genes HK-20 and HK-13 were involved in the glycine degradation pathway, which is consistent with the fundamental theory of metabolic engineering [27,28]. These results demonstrate that our CDCK-SMIA platform can be effectively applied for reprogramming metabolic pathways to obtain promising bioproduction performance.

### Interfering effects of MRP targeting HKs on glycine production

**Fig. 7.**
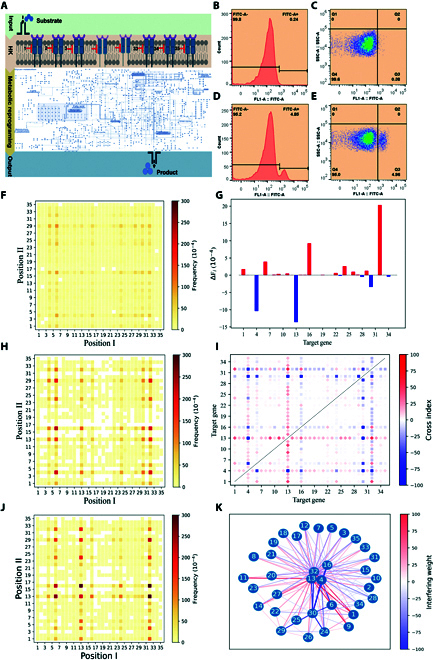
Effects of metabolic reprogramming (MRP) targeting histidine kinases (HKs) on glycine production in *Vibrio alginolyticus* FA2. (A) Schematic diagram of the combinational knockdown of HKs triggering MRP that affects metabolite synthesis. We named and numbered 1 to 35 for the HK genes of the *V. alginolyticus* FA2. (B and C) Flow cytometry diagrams showing representative distributions of the CRISPRi-mediated dual-gene combinational knockdown (CDCK) variant cells according to their green fluorescence for the cells without sgRNA library (control group). Q4/ or FITC-A−: cells below the threshold for the “LOW” state; Q3/ or FITC-A+: cells in “HIGH” state. (D and E) Flow cytometry diagrams showing representative distributions of the CDCK variant cells according to their green fluorescence for the cells with sgRNA library (experiments group). (F) Frequency of the different dual-sgRNA cassettes in the *E. coli* HK-L1 library. (G) Contribution of the single knockdown gene for the survival-frequency change of the coculture. (H) Frequency of the different dual-sgRNA cassettes of HK-L2 library in *V. alginolyticus* FA2 containing dCas9 before arabinose-based induction of dCas9 protein. (I) Contribution of the combinational dual-gene knockdown for the survival-frequency change of the coculture. (J) Frequency of the different dual-sgRNA cassettes of HK-L2 library in dCas9-containing *V. alginolyticus* FA2 after induction of dCas9 protein. (K) Gene-to-gene interaction networks analysis. The connection of different nodes indicates interaction effects between 2 genes. When the edge gets thicker, the interaction effects are more substantial. The red edge color represents the positive interaction, and the blue indicates the negative interaction. All the HK genes of the *V. alginolyticus* FA2 are numbered from 1 to 35. (For interpretation of the references to color in this figure legend, the reader is referred to the web version of this article.)

Unlike the genes of the glycine pathway, the sensory genes correlate indirectly with glycine metabolism. However, it is still reasonable to suggest that they can influence glycine metabolism through higher-order gene interactions. Therefore, we investigated the interfering effects of MRP on glycine production by coupling the dual-gene combinational knockdown library of HKs with the glycine-on mediated high-throughput screening method (Fig. 7A). As shown in Fig. 7E, the cells in the experimental group were divided into 2 groups on the basis of the eGFP fluorescence intensities: Q3 (cells with high eGFP fluorescence value, FITC-A+) and Q4 (cell with low eGFP fluorescence value, FITC-A−), with proportions of 4.96% and 95.0%, respectively. 99.6% of the cells in the control group were located in the Q4 (Fig. 7C). FACS results showed that the percentages of FITC-A+ were 0.24% and 4.85% in the control and experimental groups (Fig. 7B and D). Therefore, it was indicated that the glycine production of the Q4 quadrant cells might be higher than those of Q3 since the higher fluorescence coupled with the higher glycine concentration using the glycine-on riboswitch.

For the NGS process, we investigated the numbers of reads corresponding to the designed dual-sgRNA cassettes, resulting in the mapping ratios of ~90.2%, 85.3%, and 80.5% for HK-L1, HK-L2, and HK-S, respectively (Table S26). The high mapping ratios suggest that the procedures of CDCK are sufficiently reliable. The frequencies of the different dual-sgRNA cassettes were relatively uniform in the HK-L1 group (Fig. 7F). In contrast, position enrichments and decreases were observed in the HK-L2 (Fig. 7H) and HK-S (Fig. 7J) groups. These findings indicate that the CRISPRi system functioned well with the effectiveness of enrichment and decreasing variant cells.

After ranking the frequencies of the dual-sgRNA cassettes obtained from NGS data, the dual-sgRNA cassette combinations of the top 3 frequencies in the HK-S group were the combination of HK-16 and HK-1 (16-1, 2.0-fold), HK-32, and HK-2 (32-2, 1.8-fold), and HK-32 and HK-1 (32-1, 1.7-fold), as compared to the control group, respectively (Tables S8, S10, and S11). In the SMIA of the sorted cells (HK-S), we can find that the genes HK-1, HK-6, HK-16, HK-22, HK-24, HK-29, and HK-32 contribute positively to the accumulation of glycine. In contrast, the genes HK-4, HK-30, and HK-13 contribute negatively to glycine production (Fig. 7G). According to the dual-gene combinational contribution analysis, many HK pairs are associated with glycine production, of which 13 dual-gene knockdowns may increase glycine production, and 11 dual-gene knockdowns may reduce glycine production (Fig. 7I). We also integrated data analysis results from multiple experiments to form a comprehensive gene interaction network which shows that the gene pairs 13-30, 4-30, 32-30, and 29-30 negatively interact in enhance glycine production while gene pairs 13-11, 9-13, 1-13 positively interacts in enhance glycine accumulation (Fig. 7K). For further validation, we independently constructed targeting strains 16-1, 32-2, and 32-1 (strain 16-1, strain 32-2, strain 32-1). The cells were then cultured under the same conditions described above. FACS revealed that the fluorescence intensities of strains 16–1, 32–2, and 32–1 were 4.2-fold, 4.3-fold, and 4.8-fold higher than the control strain (Fig. S5), which were consistent with the NGS data analysis. Further, we extracted the intracellular products of the strains and measured amino acid content in 3 repeated experiments using the Amino Acid Analyzer (Figs. S8 to S10). Strain 16-1 was found to produce 3530.0 ng/ml, 3686.4 ng/ml and 3894.6 ng/ml of glycine in 20 h in 3 repeated independent experiments, respectively, under the same culture conditions as described above. All 3 experimental data indicate an increased yield of approximately 3.1% compared to their control (Figs. S8 to S10). The results show that our CDCK-SMIA platform can be used to investigate the effects of MRP**-**targeting HKs on glycine production in the *V. alginolyticus* FA2.

## Discussion

Decoding and controlling chaotic metabolic networks have long been the central demand of biological research due to the general relevance of metabolism to various aspects. For example, it is increasingly recognized that metabolism is closely related to antibiotic resistance, which is one of the urgent threats to both public and global health [29]. Studies show that the evolution of tolerance against antibiotics strongly depends on the reprogramming of bacterial metabolism [30,31]. Synthetic biology is one of the most widely discussed and notable technologies in the 21st century [32]. As is well known, synthetic biology has been achieving the goal by precisely rewiring metabolic networks [33], which could also be called MRP. However, desired MRP is difficult to achieve because of the metabolic network complexity [34,35]. Therefore, it is of significance to precisely reprogram the metabolic networks for desired phenotype or target products.

Here, we established an MRP platform consisting of CDCK and SMIA for efficiently reprogramming the metabolic networks. With this platform, we can decode the interfering effects of the “sensitive node” genes of the metabolic networks (e.g., HKs) for MRP from a global metabolic perspective. We successfully applied our CDCK-SMIA platform to investigate the effects of MRP targeting HKs on antibiotic resistance of the *V. alginolyticus* FA2 (Figs. 4 and 5). We also manipulated the HKs and glycine pathway genes; both focused on modulating glycine production (Figs. 6 and 7). Moreover, we established a 1-stop web service for MRP research, where users can automatically design experiments. After wet-lab experiments, the services can also be used to conduct data analysis by simply uploading the raw data. Our CDCK-SMIA platform serves as a smart platform for implementing MRP through the global combinational interference of the “sensitive node” genes in the metabolic network.

Natural sensory proteins like HKs can be seen as hub nodes of signal transduction networks because they link diverse intracellular and extracellular stimuli to specific cellular responses, including development, cell division, and antibiotic resistance [36]. Therefore, sensory proteins are also considered to reside on the typical “sensitive nodes” of the metabolic networks, which can trigger natural MRP upon perceiving environmental fluctuation. While metabolic engineering has traditionally focused on material flow (metabolites) and energy flow (cofactors) [37], we conducted MRP based on the “sensitive nodes” for studying metabolic engineering from the perspective of information flow. On the other hand, it is increasingly realized that the cross-talk of signaling pathways is not a nondesired drawback that people thought; on the contrary, it may bring additional advantages such as coordinating physiological responses, improving the accuracy of concentration identification, and allowing massive signal recognitions with compact receptor array [38,39]. Using our MRP platform, we systematically decode the relationship between HKs and antibiotic resistance by identifying their contributions to ampicillin resistance on both single-gene and dual-gene interference levels. We found that the impacts upon modulating multiple-gene interactions were different from impacts upon modulating the corresponding genes individually, as suggested by the significant changes in metabolic status in *V. alginolyticus* FA2 through combinational gene knockdown via our MRP platform (Figs. 4 and 5). Apparently, operating these “sensitive nodes” genes can be considered a smart MRP since only manipulating compact genes (e. g., 35 HK genes) can achieve the goals of the MRP with sufficient diversity, for instance.

In addition to HK genes, the genes that reside on “sensitive nodes” of the metabolic networks also include those that can cause global changes in the metabolic state, such as silent genes [8] and regulator proteins [16]. We have incorporated these “sensitive node” genes in our web service to improve the complement of our MRP strategy, thereby broadening the applications. Notably, dual-sgRNA libraries can be constructed using the gene shuffling method based on PCR, while a dual-sgRNA library targeting n × n gene pairs can be obtained by synthesizing only n pairs of primers (Fig. 3A), indicating the cost-efficiency of our platform. Note that the CRISPRi system used in our CDCK approach has been previously demonstrated as effective in many hosts, including mammalian cells and bacteria [23,40,41]; we, therefore, anticipate that our platform should be applicable to a wide variety of species.

Metabolic remodeling is an essential aspect of microbial physiology; for example, drug resistance, a significant threat to global health, has been associated with MRP [42,43]. In this study, we found several HK genes associated with ampicillin antibiotics in *Vibrio* (Figs. 4 and 5), such as HK-16 (*cpxA*). This result is consistent with a previous study, which reported that HK-16 (*cpxA*) was associated with the antibiotics in *E. coli* and *Klebsiella pneumoniae* [44,45]. These findings support that our platform enables investigations of microbial physiology. More interestingly, the multiple-gene interactions rather than the independence effects of the genes 24 (*sasA_8*) and 30 (*04288*, hypothetical HK protein) were found to decrease ampicillin antibiotics resistance in *V. alginolyticus* FA2 (Figs. 4 and 5), as experimentally verified using 3 methods: q-PCR, growth curve analysis, and confocal microscopy (Fig. 5D to L). On the basis of these results, the combinational knockdown of the HK genes *sasA_8* and 04288 can achieve the desired effects on antibiotic resistance compared to independent knockdowns, which have never been reported in bacteria. Our platform can be used to discover new targets for bacteria antibiotic resistance and is suggested to be effective for studying the physiology of microorganisms, especially the effects of multiple-gene interactions.

Controlling metabolic flux is well known to be of significance for synthetic biology, which is often considered a “green” technology for producing bulk and fine chemicals [46–50]. We attempted to control metabolic flux to improve glycine production due to its importance in the food, pharmaceutical, chemical, and agricultural industries [51]. We first tested our MRP platform by directly targeting the natural glycine pathway and found that combinational knockdown genes in the glycine degradation pathway (e.g., *gcvT* and *ltaE*) could increase glycine production (Fig. 6). The results were consistent with that of a previous report [27], indicating the reliability of our platform. Secondly, the effects of the natural sensory proteins on glycine production by targeting HKs were also investigated. Glycine production was found to be increased by the simultaneous knockdown of the HK genes *cpxA* and *btsS* in the *V. alginolyticus* FA2 (Fig. S8), which was never reported before. Our finding suggested that the production of biochemicals could be improved by harnessing the interference between the genes of global regulatory factors (e.g., HKs). Such methodology can be adopted in the biomanufacturing field to enhance production through MRP, which is beyond the traditional approaches.

In summary, we developed a smart MRP platform including the CDCK and SMIA. This platform holds great promise as a versatile platform for metabolic network reprogramming that could be suitable for a variety of fundamental and applied biological research applications, such as studying drug resistance mechanisms and biomanufacturing.

## Materials and Methods

### Strains, media, and growth conditions

All strains used in this study are listed in Table S2. *E. coli* DH5α was used for general cloning and was cultivated at 37 °C in the LB medium. *V. alginolyticus* FA2 [21] was used for constructing the CRISPRi-mediated dual-gene combination knockdown system and was cultivated at 37 °C in the LB3 (LB with 3% NaCl, w/w) medium. The *V. alginolyticus* FA2 harboring the glycine-riboswitch plasmid was cultivated in the M9 minimal medium [26]. Chloramphenicol (CmR, 10 μg/ml) or tetracycline (TetR, 5 μg/ml) was added to the medium as required.

### Plasmid construction

All plasmids used in this study are listed in Table S2. Primers used for plasmid construction are detailed in Table S1. We used 2 × Phanta Max Master Mix (Vazyme Biotech Co., Ltd), and 2 × CloneExpress Mix (Vazyme Biotech Co., Ltd) to construct the plasmids. Golden Gate Assembly was used for designing and building the sgRNA library [52]. The dCas9 and sgRNA chimera genes were synthesized from the previously described vectors [23]. The gene encoding dCas9 was inserted into the pACYCdute-1 vector containing a chloramphenicol-selectable marker and a p15A replication origin, resulting in the pdCas9 plasmid. The sgRNA chimera was inserted into the pE2a vector with a TetR-selectable marker and ColE1 replication origin, resulting in the pE2a-sgRNA plasmid. Then, the 2 sgRNA chimeras were ligated into the pE2a-plasmid, generating the pE2a-2sgRNA plasmid. The pE2a-tool plasmid was constructed by inserting the *ccdB* gene into the N20 position of the pE2a-sgRNA plasmid using Golden Gate Assembly guidelines (Fig. 3A). The *mrfp* and *egfp* genes were synthesized and inserted into the pE2a-sgRNA plasmid to generate the fluorescence marker plasmids pE2a-mRFP-sgRNA and pE2a-mRFP-eGFP-sgRNA.

### Construction and testing of the glycine-on riboswitch

To assess the potential usability of glycine-on riboswitch in *V. alginolyticus* FA2, different promoters and selective marker gene *egfp* were used to construct the plasmids, resulting in pP_trc_GRS-eGFP (promoter P_trc_), pP_J23100_GRS-eGFP (promoter P_J23100_), and pP_native_GRS-eGFP (the potential promoter of the VC1442 gene in *V. cholerae)*; then, we deleted the aptamer domains used for binding glycine in these plasmids, resulting in the control plasmids pP_trc_GRS-Del-eGFP, pP_J23100_GRS-Del-eGFP, and pP_native_GRS-Del-eGFP. Detailed sequences of constructed plasmids are listed in Table S3. All the plasmids were transferred into the *V. alginolyticus* FA2 to test whether the glycine-on riboswitch works in the *V. alginolyticus* FA2. We cultivated the strains of the experiment group and their controls in the M9 medium [26] supplemented with various glycine concentrations (0 to 100 mM). Then, the levels of eGFP fluorescence were quantified using flow cytometry (BD Biosciences FACSAria III).

### Design and construction of the Dual-sgRNA library

The primers of the dual-sgRNA library were designed via our 1-stop website according to the guidelines, which are shown in Fig. 3A. Dual-sgRNA library genes were generated from PCR amplification using the pE2a-2sgRNA plasmid as a template. Then, the PCR products containing the dual-sgRNA library were inserted into the pE2a-tool plasmid using the *BbsI* restriction enzyme and T4 DNA ligase. The resulting products were then transformed into *E. coli* DH5α competent cells. We washed out around 40,000 transformants with the liquid LB medium and mixed them as the library 1 strain (L1). The plasmids of the L1 strains were extracted as the dual-sgRNA library plasmid for the subsequent experiments.

### CRISPRi-mediated dual-gene combinational knockdown

The dCas9 protein and pP_trc_GRS-eGFP were combined into a single plasmid. Then, the resulting dCas9-pP_trc_GRS-eGFP plasmid was transferred into *V. alginolyticus* FA2 (termed the “dCas9-pP_trc_GRS-eGFP strain”). Next, the dual-sgRNA library plasmid was transferred into the dCas9-pP_trc_GRS-eGFP strain as described above. Then, we washed out around 40,000 transformants with the liquid LB3 medium and mixed them evenly as the initially constructed *V. alginolyticus* FA2 containing dCas9 and pP_trc_GRS-eGFP (Fig. S2). Then, arabinose and IPTG were used to induce the expression of dCas9 and eGFP. On the basis of FACS, variant CDCK cells with high fluorescence values were screened out compared with the control. Next, we extracted the plasmid from these sorted cells as the templates, and the dual-sgRNA fragments were amplified by PCR. Then, the PCR products were assessed by NGS.

### Electroporation transformation protocol for *V. alginolyticus* FA2

The *V. alginolyticus* FA2 cultures were inoculated into the LB3 medium, cultivated at 37 °C, 200 rpm overnight, and then diluted 1:100 into the fresh medium. Cells were harvested at OD_600_ nm ~ 0.5 and pelleted by centrifugation at 4000 rpm for 5 min at 4 °C. The pellet was washed 3 times with precooled buffer (680 mM sucrose and 7 mM K_2_HPO_4_ [pH 7.0]), and the final cell pellet was resuspended using the buffer as a 200-fold concentrate of the initial culture. For transformation, 50 ng of plasmid DNA was added into the 100 μl of the competent cells in 0.1-mm cuvettes and electroporated using an electroporator (MicroPulser #1652100, Bio-Rad) at 900 V, 25 μF, and 200 Ω, followed by recovery in recovery media (BHI + v2 salt +680 mM sucrose) for 1 h at 37 °C, 200 rpm, and subsequent plating on the selective medium. Brain heart infusion medium (BHI) + v2 salt (1 L): 37 g BHI (Becton-Dickinson, Heidelberg, Germany), 204 mM NaCl, 4.2 mM KCl, 23.1 mM MgCl_2_. The plates were incubated for around 8 h at 37 °C.

### Flow cytometry and reporter assays

The strains were cultivated in the minimal M9 medium and contained the appropriate antibiotics at 37 °C, 200 rpm. When the cells reached the mid-log phase, eGFP intensity was measured using a flow cytometer (BD Biosciences FACSAria III) equipped with a high-throughput sampler. Cells were sampled with a slow flow rate until at least 20,000 cells had been harvested. We used CytExpert software (Beckman Coulter) and FlowJo software (BD) to analyze the FACS data by gating on a polygonal region containing at least 80% cell population in the forward scatter-side scatter plot. All the experiments were conducted repeatedly 3 times. To quantify the mRFP and eGFP signals, the fluorescence was read at excitation/emission of 584/607 nm, and 499/503 nm, respectively, using a Multifunctional Microplate Reader (Tecan Spark Synergy2).

### NGS data analysis and SMIA assay

After the high-throughput screening of the cell libraries, the survived cells were sorted out on the basis of the interests' fluorescence values or other screening criteria. After that, we extracted the plasmids from these cells and amplified the dual-sgRNA cassette fragments using PCR. NGS assessed the PCR products. We developed an integrated python3 software package to analyze the sequencing data, including sgRNA design and NGS data processing functions (Table S6). The overall workflow on how to design the CDCK platform is shown in Fig. 3A. We developed the dual-sgRNAs targeting n × n gene pairs by synthesizing only n pairs of primers using gene shuffling and Golden Gate Assembly. To facilitate the use of the platform, we developed a 1-stop website in which the users only need to enter the name of the candidate microorganism. The designed primers by the website are available. The users can conduct similar research more efficiently. The website is available at the URL of https://smrp.sjtu.edu.cn.

The website's working procedures are as follows: Firstly, the “Statistics of survival frequency” is based on the NGS data obtained from the wet-lab experiment of our proposed smart MRP (SMRP). The SMRP platform consists of a CDCK platform and SMIA. We conducted the CDCK by constructing the dual-sgRNA libraries coupled with the high-throughput screening method. The SMIA consists of 2 parts of “Statistics of survival frequency” and “Comparison analysis.” As shown in the supplementary SMIA analysis report, we counted the survival frequencies (per 10^4^ times) of different dual-sgRNA cassettes in the survived cells through NGS. After submitting the control and sample data, our system of python3 package can compare the input data with the database obtained from the “Design” mode and draw the abundance distribution maps. We named and numbered 1 to 35 for all the target genes for convenience. The “Position I" and “Position II" represent the first and second sgRNA targeting the genes of interest.

Moreover, the “Comparison analysis” is based on the data of survival frequencies obtained from the process described above. Take Fig. 3D as an example, and it shows the results of SMIA obtained by comparing the control and sample data. For the SMIA assay, we defined *i*/or *j*, ∆*F*_*i*,_ and *C_ij_* as the gene number, frequency of single-gene contribution, and frequency of dual-gene contribution (defined as cross-index), respectively (Fig. 3C). *F* is the frequency value obtained experimentally or inferred from experimental data. *F_ij_* can be calculated as in Eq. 1 using the original data (Tables S7–S24). Theoretically, the position effects of genes i and j are the same, as shown in Eq. 5. Equation 6 is obtained if there is no interaction between genes i and j. ∆*F_i_* (Eq. 7) and *C_ij_* (Eq. 8) can be calculated from Eqs. 1 to 6. The represented calculation results are in Fig. 3D. If C*_ij_* > 0, genes i and j have a positive interaction, showing “+.” If *C_ij_* = 0, genes i and j do not interact with each other, as shown in white. If *C_ij_* < 0, genes i and j interact negatively and are displayed as “×.” ∆*F_i_* indicates the contribution of a single-gene knockdown to the survival rate of cell libraries. We then integrated the multiple experimental results of antibiotic selection /or FACS selection to build the comprehensive metabolic network interaction maps using the NetwokX python3 package. The connection of different nodes indicates interaction effects between 2 genes. When the edge gets thicker, the interaction effects are more substantial.

### Growth curve determination

To determine the effect of ampicillin on *V. alginolyticus* FA2 growth, the strains were grown in an LB3 medium at 37 °C, 200 rpm, supplemented with or without 5 mg/ml ampicillin (defined as amp^r^ + or amp^r^- group). The OD_600_ of the strains was determined every 5 h to assay the *V. alginolyticus* FA2 growth using a UV–vis spectrophotometer [21]. Experiments were repeated 3 times, namely 1-CT-1, 1-CT-2, and 1-CT-3 for control groups, and 1-Ex-1, 1-Ex-2, and 1-Ex-3 for experimental groups. Subsequently, the resulting strains were transferred to the fresh LB3 medium as seeds and supplemented with 5 mg/ml ampicillin again to do the second round of antibiotic screening. We harvested cells at this period for NGS. Experiments were repeated 3 times., namely 2-CT-1, 2-CT-2, and 2-CT-3 for control groups, and 2-EX-1, 2-EX-2, and 2-EX-3 for experimental groups.

### Microscopy and image analysis

eGFP- and mRFP-expressing cells were grown on the LB3 medium until the mid-log phase and observed using a Scanning Confocal Microscope (Nikon Ni-E A1 HD25). The eGFP- and mRFP-expressing cells were analyzed by testing each pixel for the presence of FITC, TRITC, and TD fluorescence under the same threshold. Images were processed and analyzed with NIS-Elements Viewer 5.21 software.

### Measurement of intracellular glycine concentration

Engineered *V. alginolyticus* FA2 cells and control cells were harvested under the same OD_600_ nm and disrupted with extraction buffer (0.1 M HCl: 10% [v:v] trichloroacetic acid = 1:2) for 1 h. Then the cells were lysed by sonication in the buffer for 15 min. The lysate was centrifuged at 10,000 rpm for 5 min, and the supernatant was assessed for glycine concentration using an Amino Acid Analyzer (Hitachi L-8900) equipped with an ion exchange resin column (size: 4.6 mm ID × 60 mm, particle size: 3 μm, resin).

## Data Availability

All data needed to evaluate the conclusions in the paper are present in the paper and/or the Supplementary Materials.

## Supplementary Materials

Supplementary Materials Section I. Figs. S1 to S13 Section II. Supplementary SMIA analysis report Section III. Supplementary discussion Section IV. Tables S1 to S28
