# Supplementary material for: Harnessing Interactional Sensory Genes for Rationally Reprogramming Chaotic Metabolism
Source: Research (Wash D C). 2022 Dec 21;2022:0017. doi: 10.34133/research.0017 (PMC11407584; doi:10.34133/research.0017)
Supplement: Supplementary Materials — Section I. Figs. S1 to S13 Section II. Supplementary SMIA analysis report Section III. Supplementary discussion Section IV. Tables S1 to S28 [file research.0017.f1.pdf]

Electronic Supplementary Information

Harnessing interactional sensory genes for rational reprogramming chaotic metabolism

Chunlin Tan, Ping Xu* and Fei Tao*

The State Key Laboratory of Microbial Metabolism, Joint International Research Laboratory of Metabolic and Developmental Sciences and School of Life Sciences and Biotechnology, Shanghai Jiao Tong University, Shanghai, China

*Corresponding author: Prof. Dr. Fei Tao or Prof. Dr. Ping Xu

Mailing address: School of Life Sciences & Biotechnology, Shanghai Jiao Tong University, Shanghai 200240, P. R. China.

E-mail: taofei@sjtu.edu.cn; pingxu@sjtu.edu.cn;

Tel: +86-21-34204066; Fax: +86-21-34206723.

Contents

Section I: Supplementary Figures S1–S13

Section II: Supplementary SMIA analysis report

Section III: Supplementary discussion

Section IV: Supplementary Tables S1–S28

Supplementary Figures

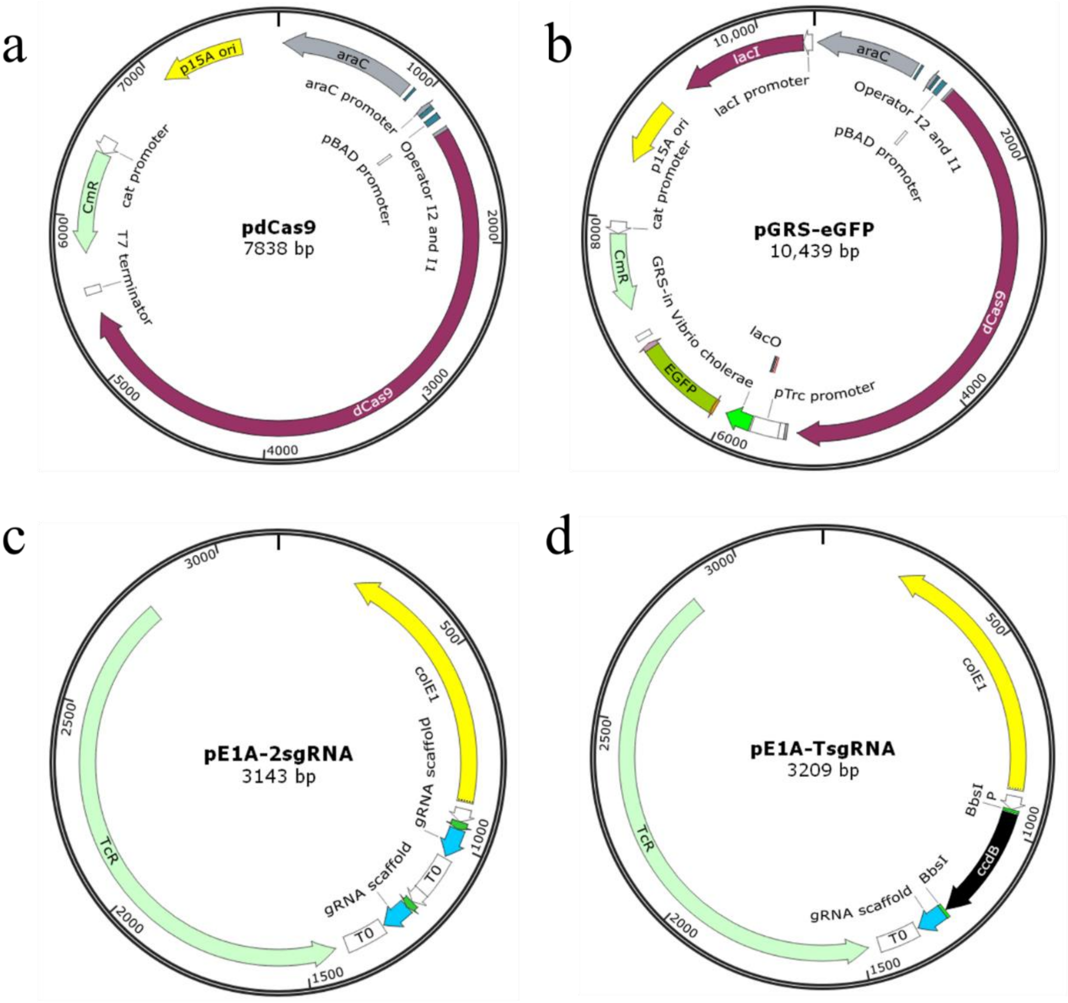

Fig. S1. Plasmid maps. (a) pdCas9 (b) pGRS-eGFP (c) pE2A-2sgRNA (d) pE2A-TsgRNA (See also Supplementary Table 4).

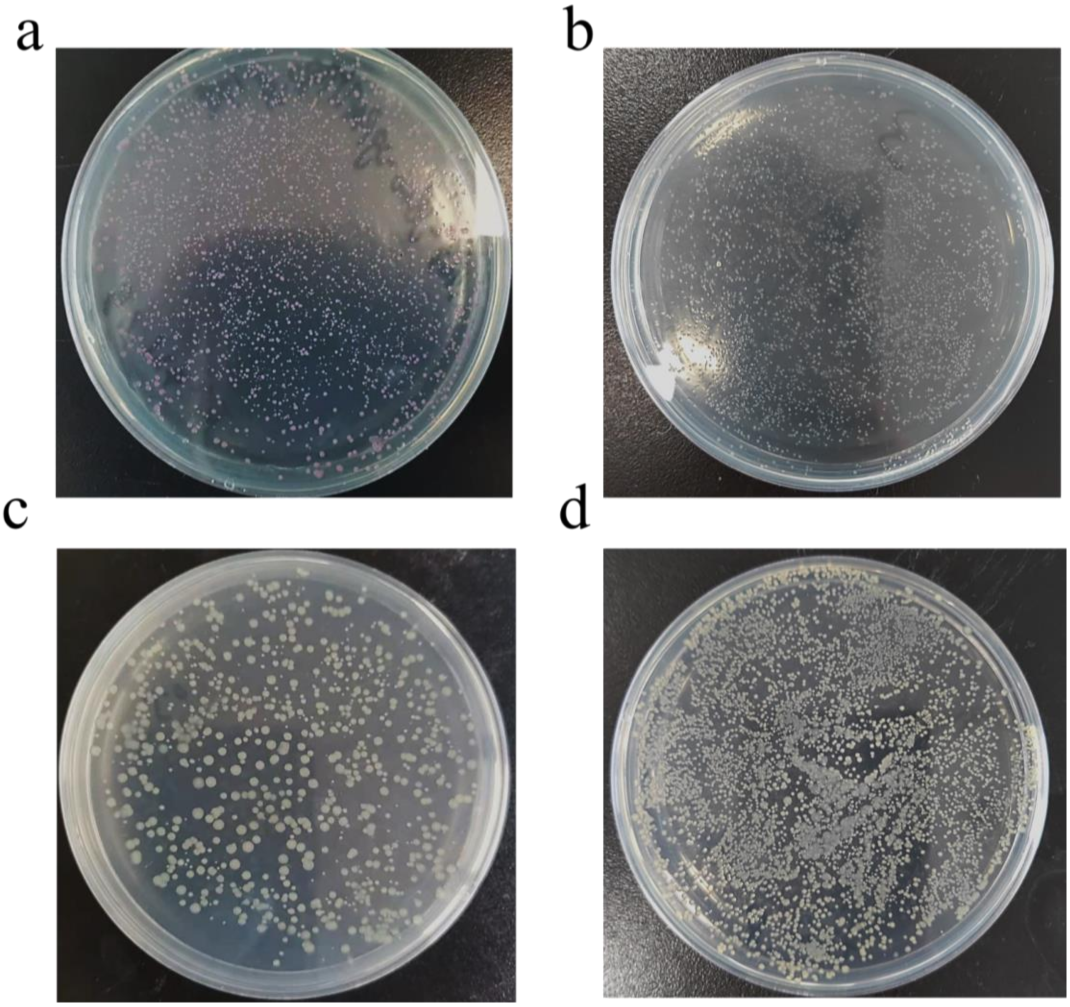

Fig. S2. Dual-sgRNA library plasmid construction. (a) and (b) The dual-sgRNA library plasmid construction in *E. coli* through PCR amplification and golden-gate assembly. (c) and (d) The dual-sgRNA library in *Vibrio* FA2. The sgRNA library plasmids were transferred into the *Vibrio* FA2 containing dCas9 and pGRS-pTrc-eGFP expression cassette by electroporation, resulting in approximately 40,000 transformants. Representative images are shown.

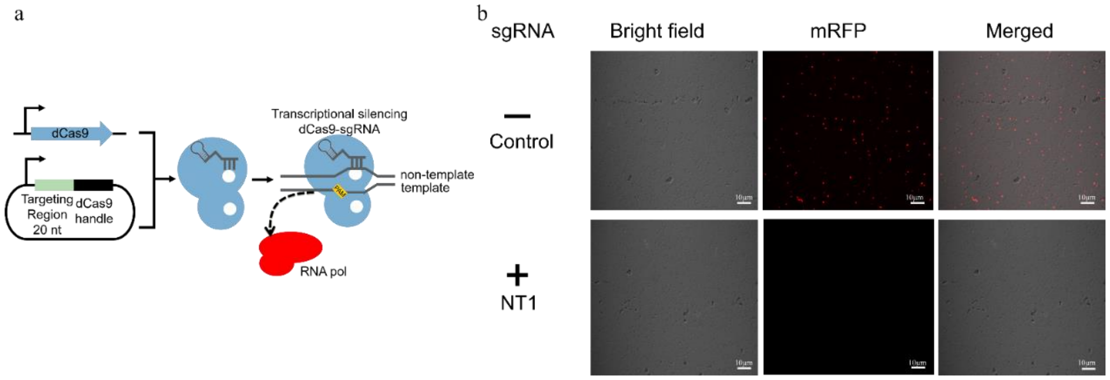

Fig. S3. Targeting specificity of the CRISPRi system in *Vibrio* FA2 (a) Schematic of the CRISPRi system in *Vibrio* FA2. (b) Microscopic images for using sgRNAs to control fluorescent proteins expression. (Top) Bright-field images of the *Vibrio* FA2 cells; (middle) RFP channel; (right) merged channel. Co-expression of sgRNA and dCas9 silences the fluorescent protein. The knockdown effect is strong using the sgRNA targeting nontemplate1 (NT1), as almost no fluorescence is observed from cells with fluorescent protein silenced. Scale bar, 10 mm. Control shows cells without sgRNA.

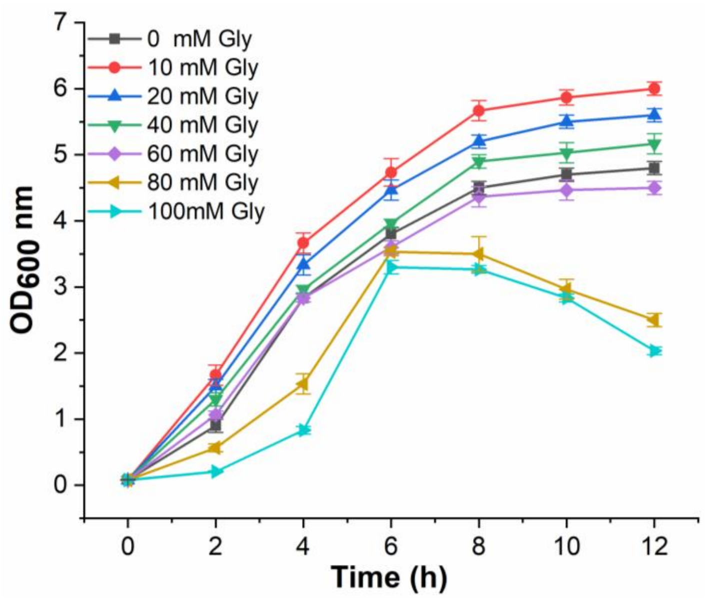

Fig. S4. Growth assay of *Vibrio* FA2 cells in an optimized M9 medium without glycine or supplemented with various concentrations of glycine.

	Sample Name	Count	Mean : FL1-A	CV : FL1-A
	negtive.fcs	19226	61.3	150
	control.fcs	18732	164	111
	sample 16-1.fcs	18516	258	152
	sample 32-1.fcs	18460	264	161
	sample 32-2.fcs	18453	297	137

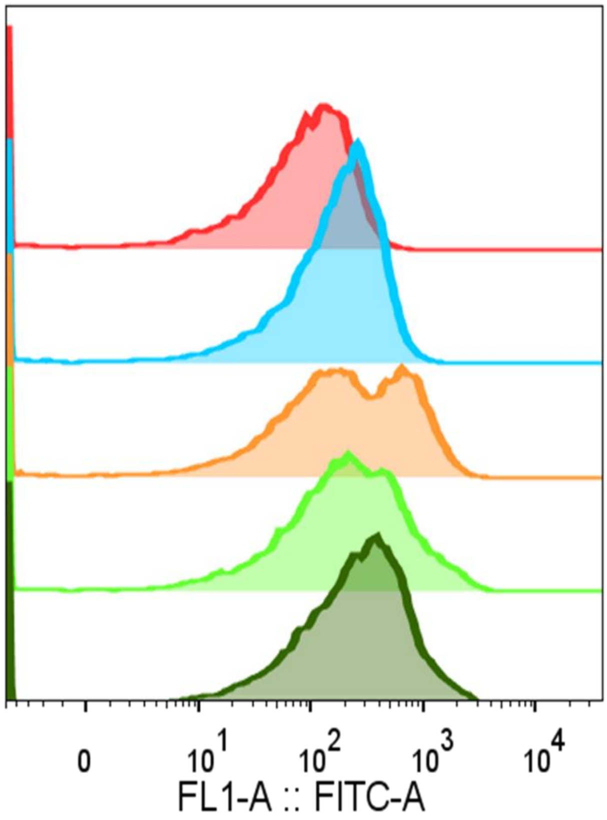

Fig. S5. Effects of metabolic reprogramming (MRP) targeting histidine kinases (HKs) on the glycine production of *Vibrio* FA2. FACS analysis of the CRISPRi-mediated dual-gene combinational knockdown (CDCK) cell libraries for 16-1, 32-1, 32-2 (experiment) and the control.

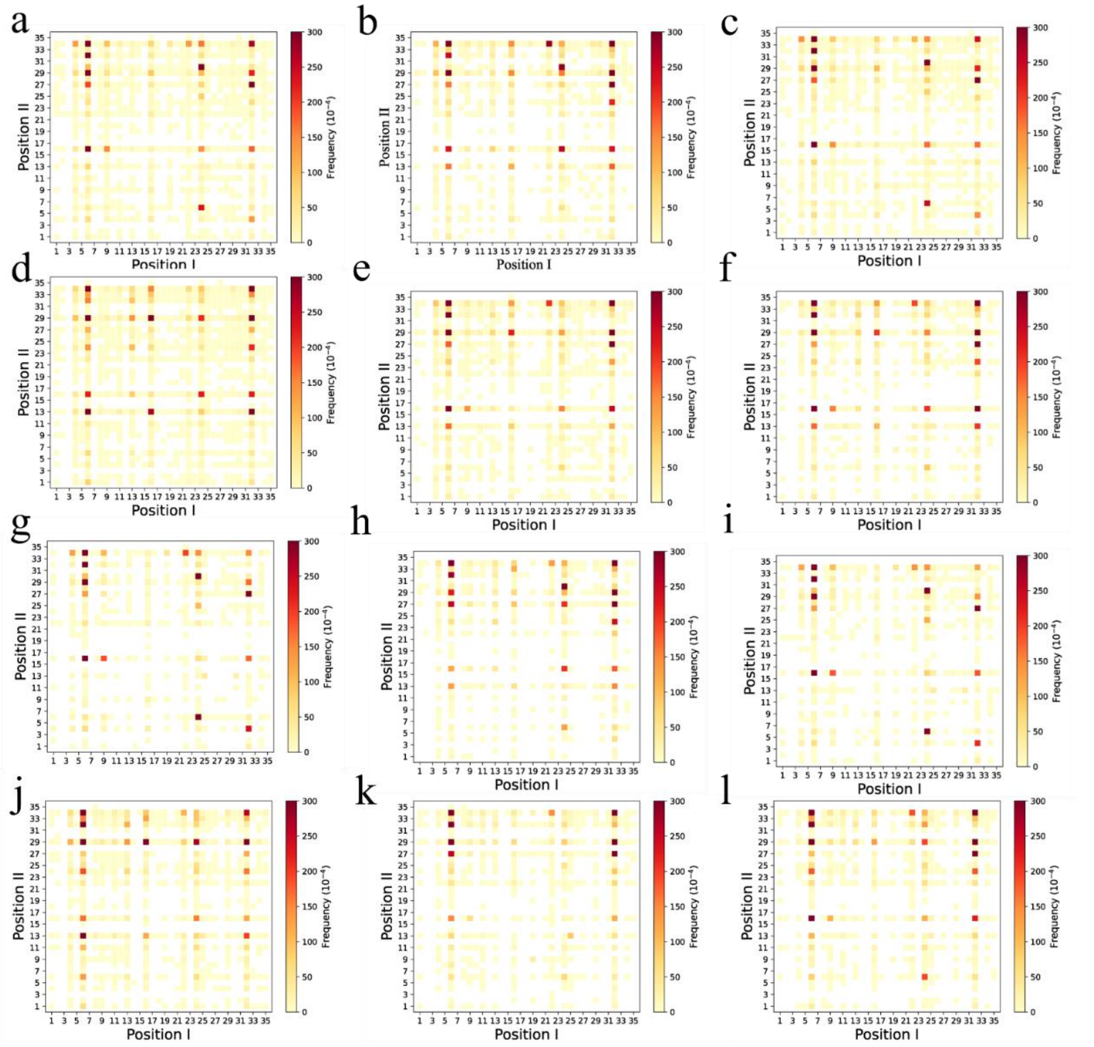

Fig. S6. Frequency of the different dual-sgRNA cassettes of HK-L2 library in dCas9-containing *Vibrio* FA2 cultivated without ampicillin (control, a, b, c, g, h, i) and with 5 mg/mL of ampicillin (experiment, d, e, f, j, k, l). Top left to bottom right: 1-CT-1, 1-CT-2, 1-CT-3; 1-Ex-1, 1-Ex-2, 1-Ex-3; 2-CT-1, 2-CT-2, 2-CT-3; 2-Ex-1, 2-Ex-2, 2-Ex-3, respectively.

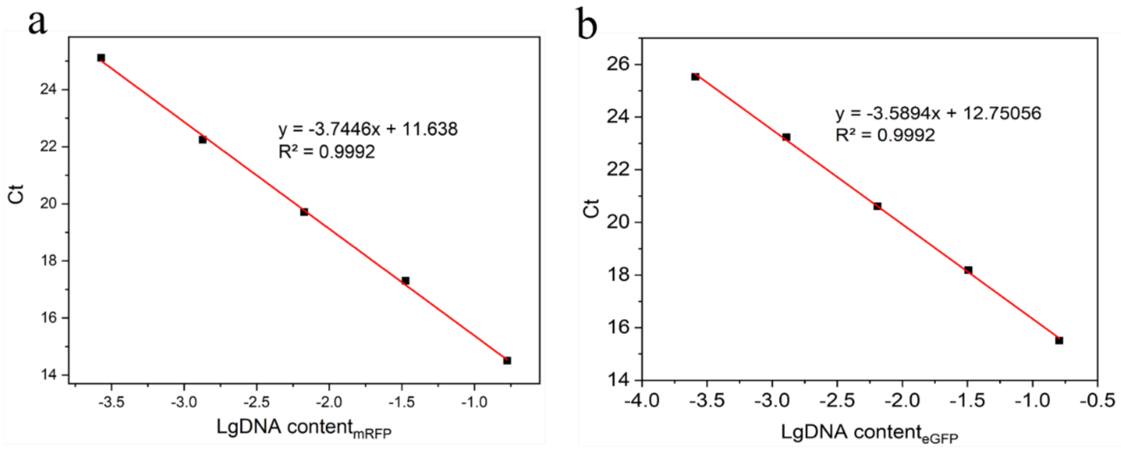

Fig. S7. Standard curves of primers in q-PCR for genes *mrfp* and *egfp*.

a

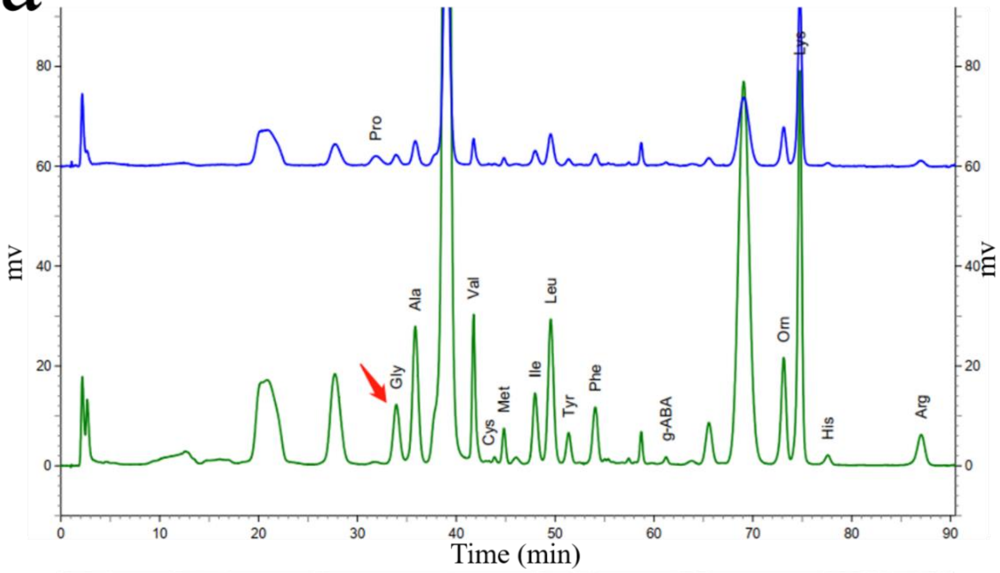

Peak #	Retention Time	Name	Area	Resolution	Mol concentration (nmol/mL)	CalcMol (ng/mL)
12	33.940	Gly	2223373	1.57	45.57	3421.06

b

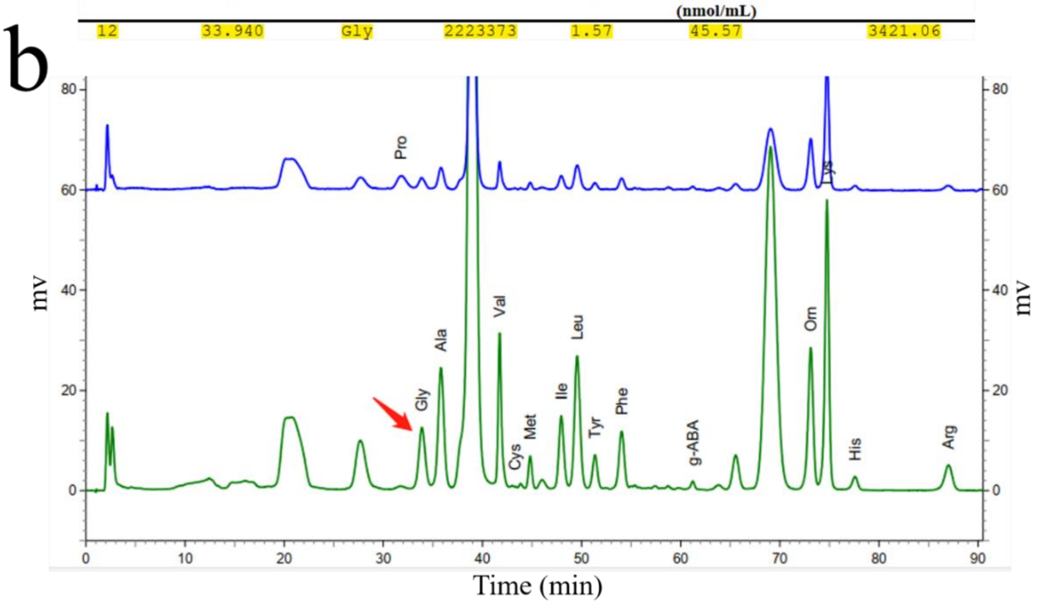

Peak #	Retention Time	Name	Area	Resolution	Mol concentration (nmol/mL)	CalcMol (ng/mL)
13	33.893	Gly	2294161	1.44	47.02	3529.98

Fig. S8. Repeated experiment-1 of HPLC analysis of glycine concentration for the control (a) and sample (b). Engineered *Vibrio* FA2 cells and control were harvested under the same OD_{600} nm and disrupted with the extraction buffer (0.1M HCl: 10% (v:v) trichloroacetic acid=1:2) for 1h. Then the cells were lysed by sonication in the buffer for 15min. The lysate of 10-fold concentrations was centrifuged at 10,000 rpm for 5min, and the supernatant was sent to Amino Acid Analyzer (Hitachi L-8900) equipped with column (size,4.6mm ID × 60mm, particle size: 3μm, resin: Hitachi special ion exchange resin)

a

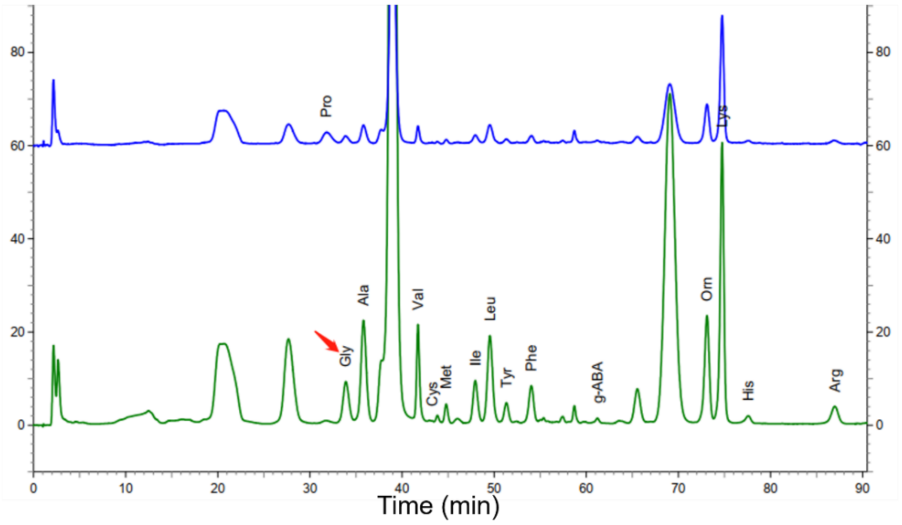

Peak #	Retention Time	Name	Area	Resolution	Mol concentration (nmol/mL)	CalcMol (ng/mL)
12	33.953	Gly	1214545	1.62	22.83	3773.80

b

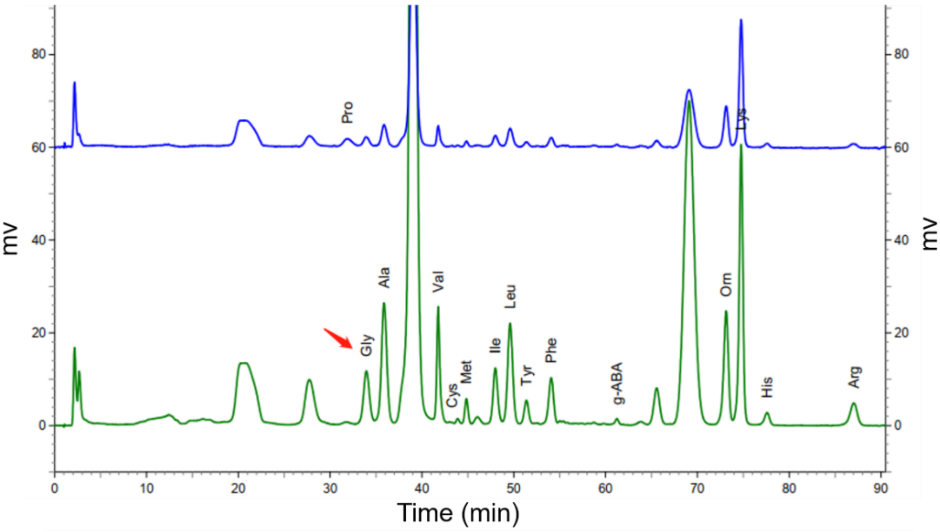

Peak #	Retention Time	Name	Area	Resolution	Mol concentration (nmol/mL)	CalcMol (ng/mL)
13	33.900	Gly	1253423	1.56	23.58	3894.60

Fig. S9. Repeated experiment-2 of HPLC analysis of glycine concentration for the control (a) and sample (b). Engineered *Vibrio* FA2 cells and control were harvested under the same OD_{600} nm and disrupted with the extraction buffer (0.1M HCl: 10% (v:v) trichloroacetic acid=1:2) for 1h. Then the cells were lysed by sonication in the buffer for 15min. The lysate of 10-fold concentrations was centrifuged at 10,000 rpm for 5 min, and the supernatant was sent to Amino Acid Analyzer (Hitachi L-8900) equipped with column (size,4.6mm ID × 60mm, particle size: 3μm, resin: Hitachi special ion exchange resin.

a

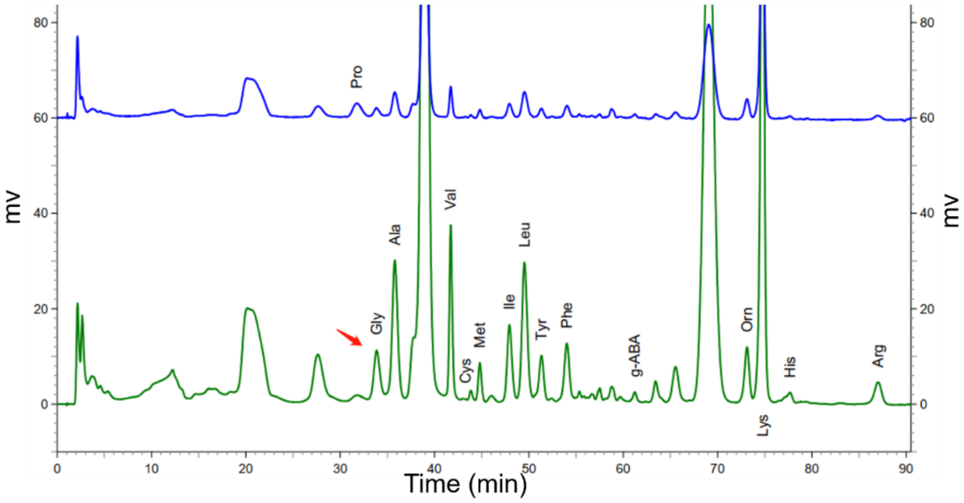

Peak #	Retention Time	Name	Area	Resolution	Mol concentration (nmol/mL)	CalcMol (ng/mL)
14	33.967	Gly	1548137	4.10	47.65	3578.98

b

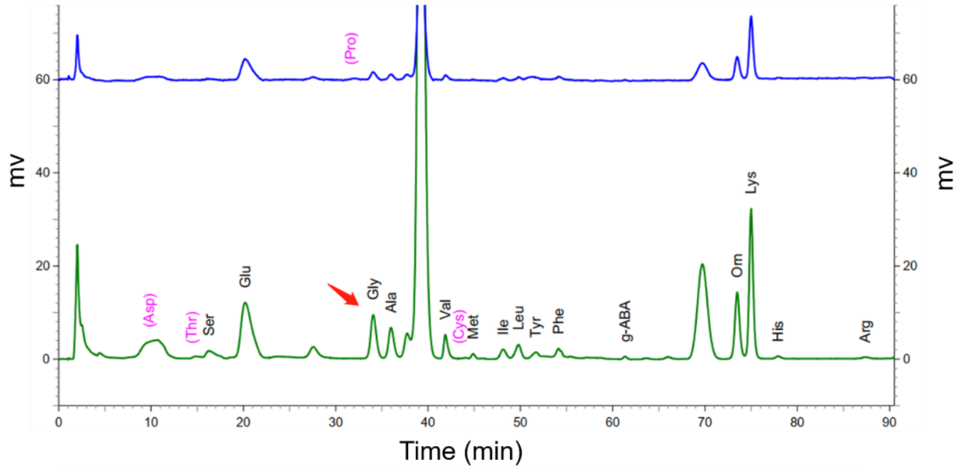

Peak #	Retention Time	Name	Area	Resolution	Mol concentration (nmol/mL)	CalcMol (ng/mL)
13	33.915	Gly	1594582	4.70	49.11	3686.35

Fig. S10. Repeated experiment-3 of HPLC analysis of glycine concentration for the control (a) and sample (b). Engineered *Vibrio* FA2 cells and control were harvested under the same OD_{600} nm and disrupted with the extraction buffer (0.1M HCl: 10% (v:v) trichloroacetic acid=1:2) for 1h. Then the cells were lysed by sonication in the buffer for 15min. The lysate of 10-fold concentrations was centrifuged at 10,000 rpm for 5 min, and the supernatant was sent to Amino Acid Analyzer (Hitachi L-8900) equipped with column (size,4.6mm ID × 60mm, particle size: 3μm, resin: Hitachi special ion exchange resin.

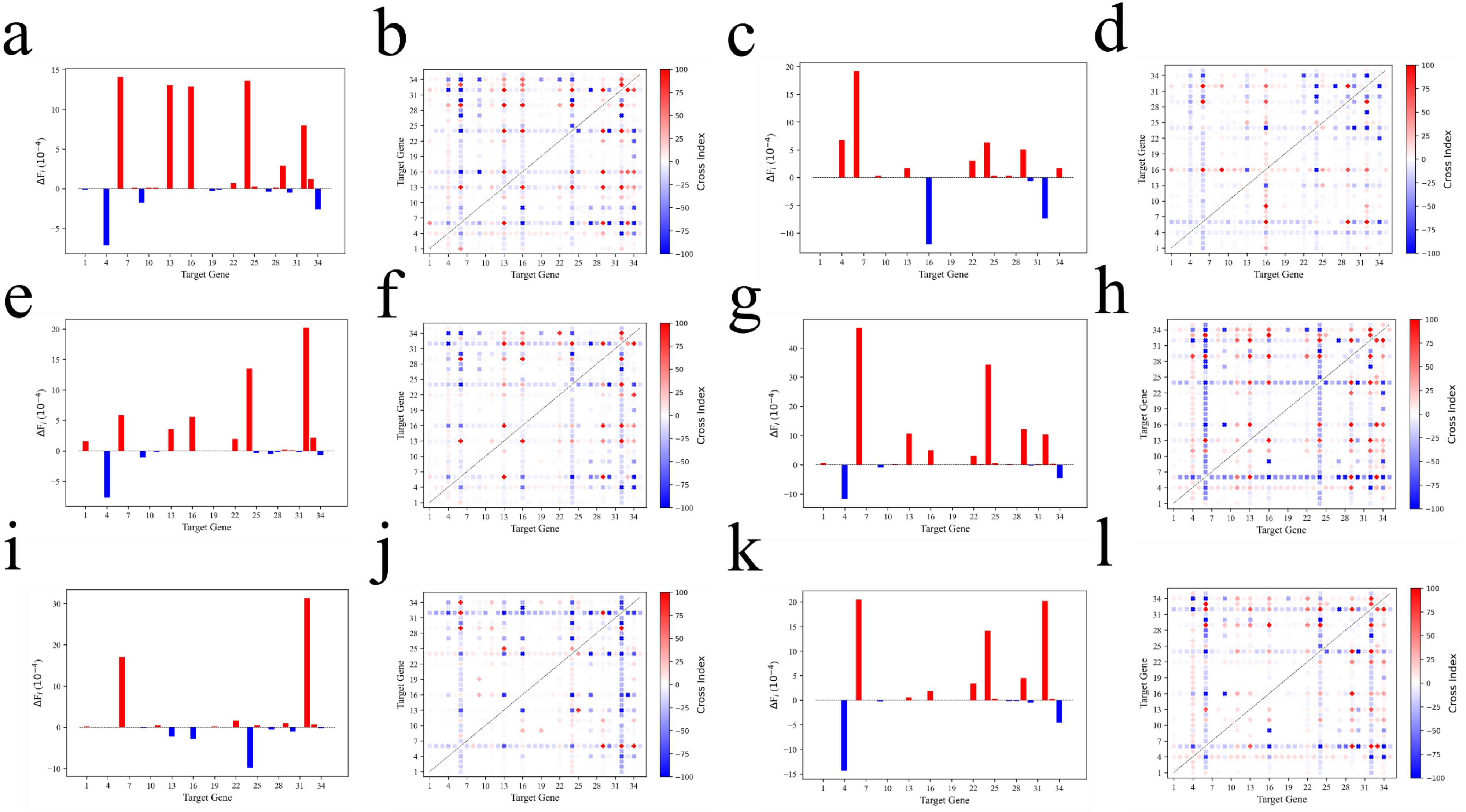

a
b
c
d
e
f
g
h
i
j
k
l
ΔF_i (10^-4)
Target Gene
Cross Index

Fig. S11. SMIA of the NGS data from dCas9-containing and HK-L2 library-containing *Vibrio* FA2 strain cultivated without ampicillin (control) and with 5 mg/mL of ampicillin (experiment). Top left to bottom right: (a) single-gene contribution of the 1-Ex-1 compared to the 1-CT-1 (b) dual-gene contribution of the 1-Ex-1 compared to the 1-CT-1 (c) single-gene contribution of the 1-Ex-2 compared to the 1-CT-2 (d) dual-gene contribution of the 1-Ex-2 compared to the 1-CT-2 (e) single-gene contribution of the 1-Ex-3 compared to the 1-CT-3 (f) dual-gene contribution of the 1-Ex-3 compared to the 1-CT-3 (g) single-gene contribution of the 2-Ex-1 compared to the 2-CT-1 (h) dual-gene contribution of the 2-Ex-1 compared to the 2-CT-1 (i) single-gene contribution of the 2-Ex-2 compared to the 2-CT-2 (j) dual-gene contribution of the 2-Ex-2 compared to the 2-CT-2 (k) single-gene contribution of the 2-Ex-3 compared to the 2-CT-3 (l) dual-gene contribution of the 2-Ex-3 compared to the 2-CT-3

	Sample Name	Subset Name	Mean:FL1-A	CV:FL1-A
	Negtive.fcs	Lymphocytes	68.9	140
	Control.fcs	Lymphocytes	377	68.0
	Sample.fcs	Lymphocytes	1051	114

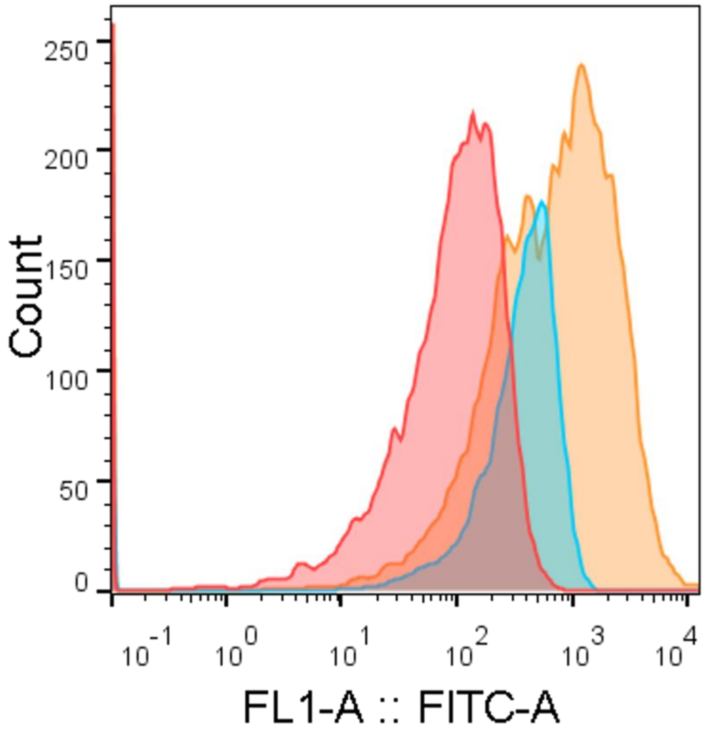

‘‘

Fig. S12 Effects of metabolic reprogramming targeting histidine kinases on the adenosine triphosphate (ATP) concentration of *Vibrio* FA2. Flow cytometry diagrams showing representative distributions of the CRISPRi-mediated dual-gene combinational knockdown variant cells according to their green fluorescence for the *Vibrio* FA2 cells without sgRNA library (control group), the *Vibrio* FA2 cells with sgRNA library (experimental group) and the negative group (wild type).

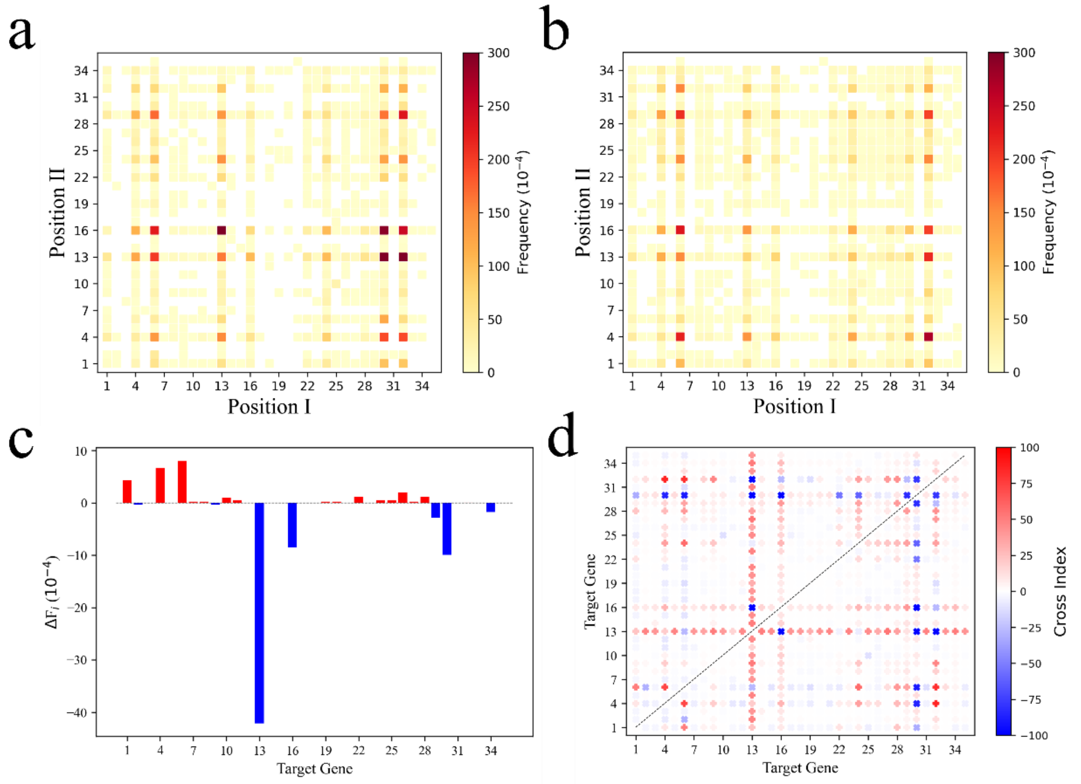

Fig. S13 Interfering effects of metabolic reprogramming targeting histidine kinases on the adenosine triphosphate (ATP) concentration of *Vibrio* FA2 strain. (a) Frequency of the different dual-sgRNA cassettes of HK-L2 library in *Vibrio* FA2 strain containing dCas9 before arabinose-based induction of dCas9 protein (control). (b) Frequency of the different dual-sgRNA cassettes of HK-L2 library in *Vibrio* FA2 strain containing dCas9 after arabinose-based induction of dCas9 protein (experiments). (c) Contribution of the single-gene knockdown to the survival-frequency change of the coculture. (d) Contribution of the combinational dual-gene knockdown for the survival-frequency change of the coculture."

SMIA ANALYSIS REPORT

1. Information of analysis job

Job name: 2-EX-2 VS 2-CK-2

Job ID: 16552918185593

Name of the uploaded control file: 2-dui-2.fq

Name of the uploaded sample file: 2-shi-2.fq

Correspoinding design ID: 1655286820415

Correspoinding strain: Vibrio alginolyticus FA2

Submision time: 2022-06-15 19:16

2. Statistics of survival frequency

We used NGS-based applicon sequencing to determine the survival frequency of different genotypes under a given screening condition. We aligned the obtained sequencing reads with the designed N20 library, counted the number of reads that could be mapped, and then calculated the percentage of survivors. All the data are normalized to allow comparison between sequencing data from different experiments.

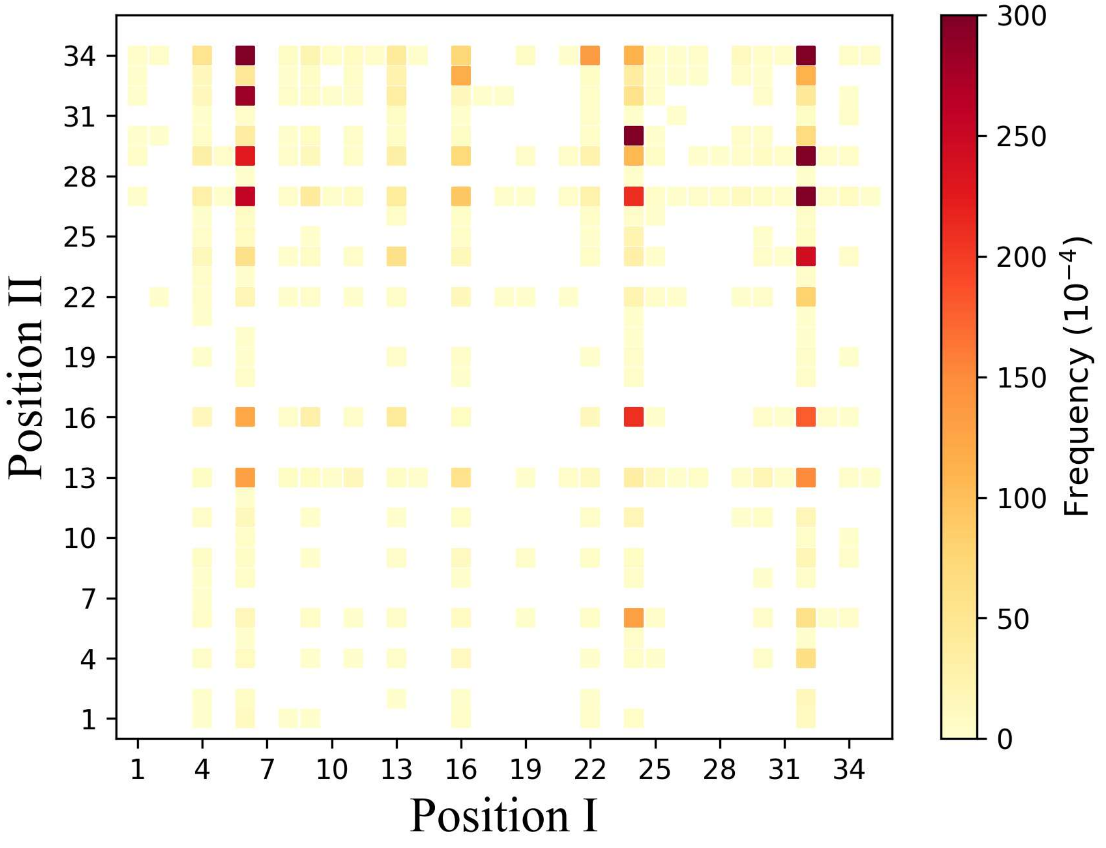

Fig. 1 | Survial frequencies related to different dual-sgRNA cassettes (Control)

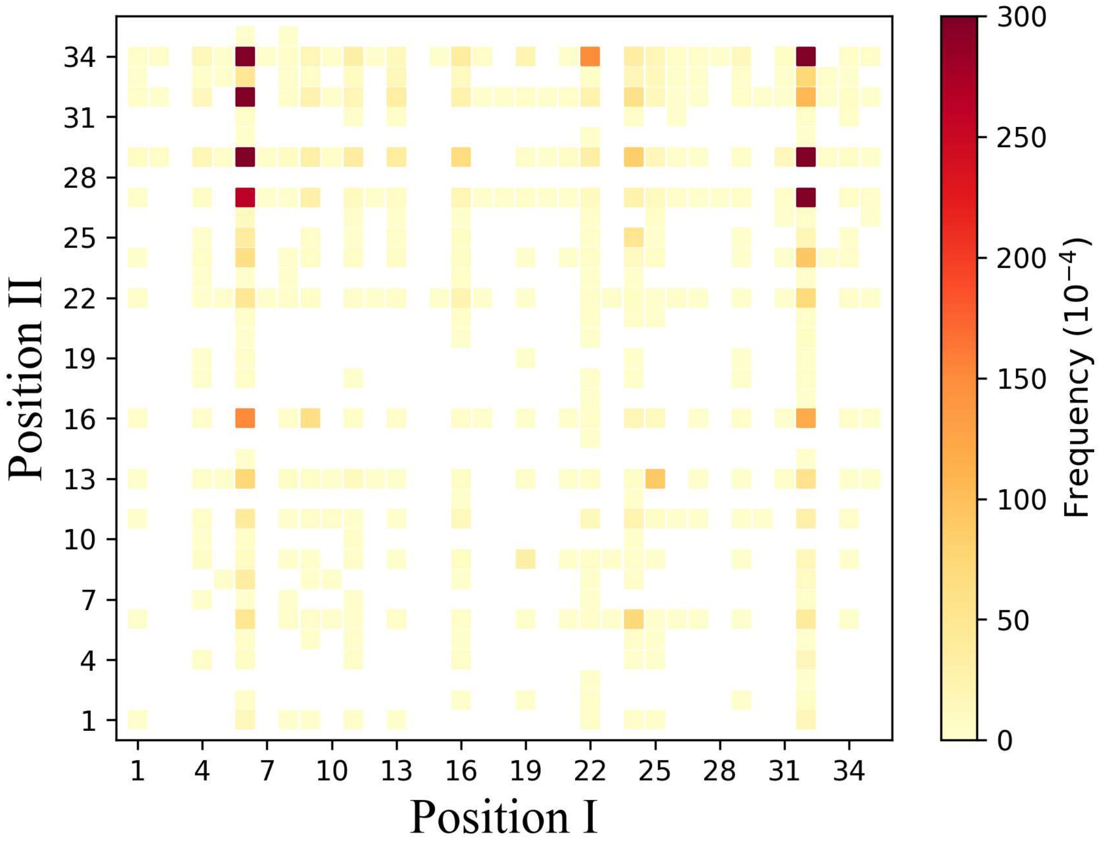

Fig. 2 | Survial frequencies related to different dual-sgRNA cassettes (Sample)

The two presented figures are the survival frequency statistics for the two samples separately. The X-axis represents the gene No. of the genes located in position 1 of the constructed plasmid, and the Y-axis represents the gene No. of the genes located in position 2 of the constructed plasmid. The color of each square represents the frequency of its corresponding genotype. The dark color represents high survival frequency.

3. Comparison analysis

Based on the above analysis, we calculated the change in survival frequency for each genotype in the control and test samples, and then we calculated the contribution of every single gene to the change in survival frequency. The calculation is based on the change in survival frequency of the genotypes that contain the same genes in position I and position II in the constructed plasmid.

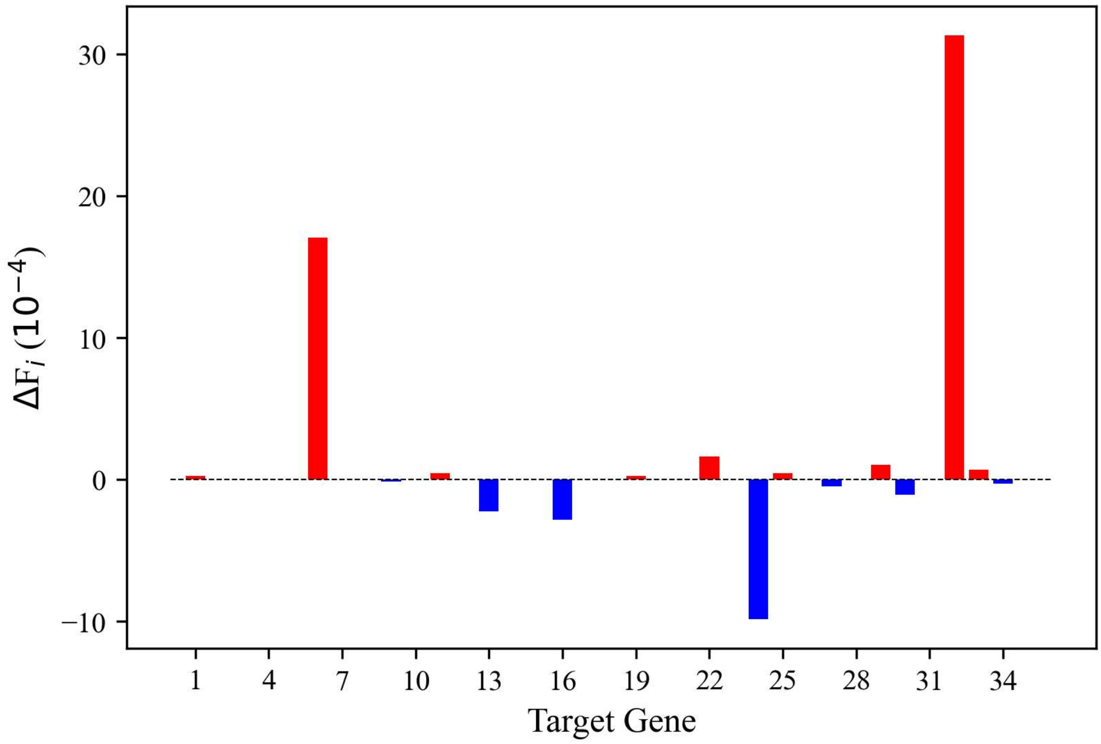

Fig. 3 | Contribution of the single knockdown gene

As shown in the figure above, the X-axis represents the gene No. of the targeted genes, and the Y-axis represents the change in the survival rate generated by the corresponding gene. Red represents an increase in survival, blue represents a decline in survival, and the height of the column represents the degree of change.

Based on single-gene contribution, it is possible to calculate the change in survival rate brought about by any combinational knocking down of two genes in the absence of gene interaction. Then comparing this value with the actual observed value can examine whether there is an interaction between genes in the actual situation.

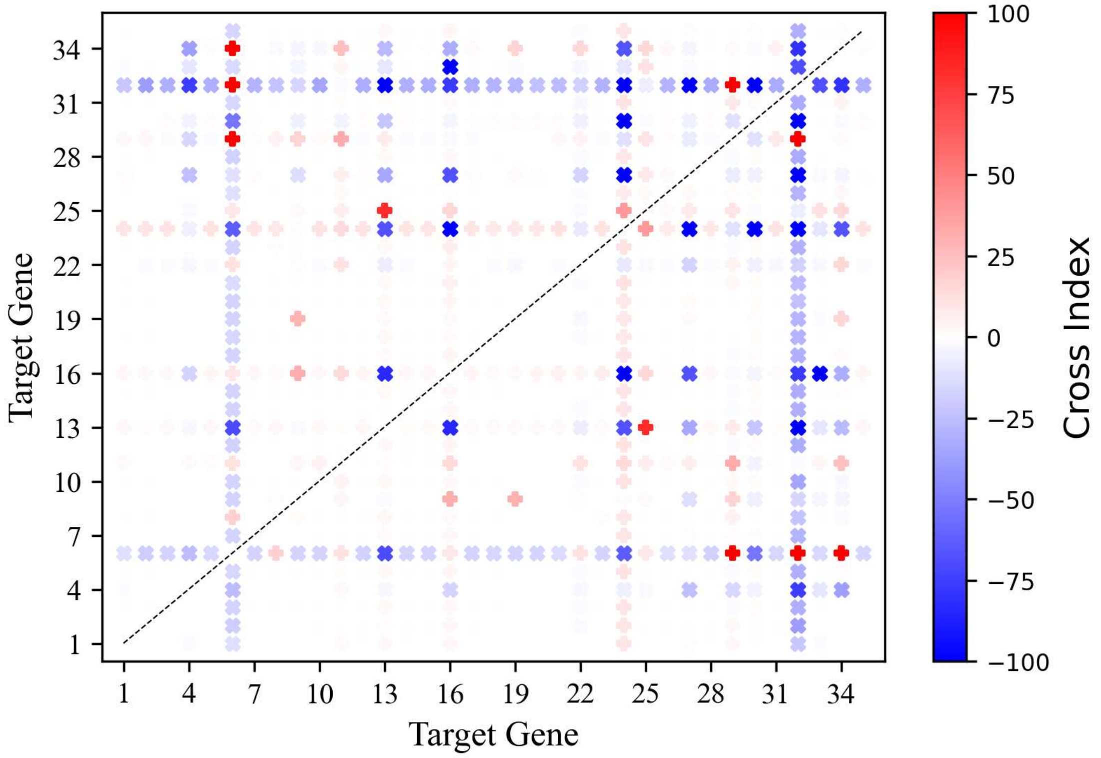

Fig. 4 | Contribution of the dual knockdown gene

As shown in the figure, the numbers in the axes represent the serial numbers of the target genes. The cross represents a positive interaction between the two genes, and the X represents a negative interaction between the two genes. The gradient of color represents the degree of interaction. Dark colors represent extensive interaction indexes.

4. Explanations

A significant core concept of SMIA analysis is survival frequency. When studying survival-related conditions, such as antibiotic resistance, it is the actual survival rate. In other types of experiments, such as sorting cells using flow cytometry, this survival rate is the probability that this genotype will be picked.

An essential premise is that we hypothesize that the physiological effects of gene knockdown by expressing sgRNAs at two locations are directly additive. This is the basis for subsequent gene interaction analysis.

Abbreviations:

SMRP: smart metabolic reprogramming
CDCK: CRISPRi-mediated dual-gene combinational knockdown
SMIA: survivorship-based metabolic interaction analysis
NGS: survivorship-based metabolic interaction analysis

5. Citation and contact

Citation:

(1).Chunlin Tan, Fei Tao, Ping Xu, Harnessing cellular perception apparatus for smart metabolic reprogramming, bioRxiv 2022.04.03.486851, doi: https://doi.org/10.1101/2022.04.03.486851.

Contact:

Prof. Dr. Fei Tao
State Key Laboratory of Microbial metabolism, School of Life Sciences & Biotechnology, Shanghai Jiao Tong University,
Shanghai 200240, People's Republic of China;
feitao@sjtu.edu.cn; +86-21-34206647.

Supplementary Discussion

Interfering Effects of MRP Targeting HKs on Adenosine Triphosphate Metabolism

Unlike the genes of the adenosine triphosphate (ATP) pathway, the sensory genes correlate indirectly with ATP metabolism. However, it is still reasonable to suggest that they can influence ATP metabolism through higher-order gene interactions. Therefore, we investigated the interfering effects of MRP on ATP metabolism by coupling the dual-gene combinational knockdown library of HKs with the ATP-sensing riboswitch [1,2] mediated high throughput screening method (Figure S12 and S13). The experimental procedure of FACS and NGS processing for ATP-sensing mediated screening was consistence with glycine-riboswitch mediated processing.

Apart from glycine-riboswitch mediated screening method, we also tried another commonly used adenosine triphosphate (ATP)-riboswitch as the screening method to test our MRP platform on investigating the effects of metabolic reprogramming targeting histidine kinases on the ATP metabolism of *Vibrio* FA2. As shown in Figure S12, the part of cells in the experimental group has high-state fluorescence values than the control. The cells with high-state fluorescence values were assessed by NGS. SMIA results showed that the frequency distribution of the dual-sgRNA cassettes is different between the experimental and control groups. Some single-gene knockdown contributions to the coculture and gene-to-gene interaction were also observed from the SMIA (Figure S13). We can reasonably speculate that our MRP platform may also play a role in investigating the interaction effects of sensory proteins on other chemicals concentration, which can be considered an important foundation for future researchers.

1. Watson, P.Y. and Fedor, M.J. (2012) The *ydaO* motif is an ATP-sensing riboswitch in *Bacillus subtilis. Nat. Chem. Biol.* 8, 963–965. 10.1038/nchembio.1095
2. Barrick, J.E. *et al.* (2004) New RNA motifs suggest an expanded scope for riboswitches in bacterial genetic control. *Proc. Natl. Acad. Sci. U. S. A.* 101, 6421–6426. 10.1073/pnas.0308014101

Table S1. All the primers used in this study.

Primers for strains construction (5'-3')	
**The mRFP-targeting sgRNAs used in Figure 3a**	
T1	GGUGAAGGUCGUCCGUACGAAGG
T2	GCAGAAAAAAACCAUGGGUUGGG
T3	UUACAAAACCGACAUCAAACUGG
NT1	GAACGGCAGCGGACCACCUU
NT2	GAUGAACUCACCGUCUUGCAGGG
NT3	GAUGGUGUAGUCUUCGUUGUGGG
**Primers for q-PCR of the eGFP and mRFP**	
eGFP-qPCR-F	TCGTCACAACATTGAAGATGGTTC
eGFP-qPCR-R	GACAAGTAGTGGTTATCAGGCAAC
mRFP-qPCR-F	CGGTGCTCTGAAAGGTGAAATC
mRFP-qPCR-R	TTCGTTGTGGGAGGTGATGTC
**Primers for targeting histidine kinase**	
1-F	gatcgaagacacactagtACACACACATCTGTTGTAAAgttttagagctagaaatagcaagt
1-R	gatcgaagactaaaacTTTACAACAGATGTGTGTGTactagtattatacctaggactgag
2-F	gatcgaagacacactagtAACTGGTCTGCCAAACCCAAgttttagagctagaaatagcaagt
2-R	gatcgaagactaaaacTTGGGTTTGGCAGACCAGTTactagtattatacctaggactgag
3-F	gatcgaagacacactagtTATCTCAAGAGCCAGGAGTTgttttagagctagaaatagcaagt
3-R	gatcgaagactaaaacAACTCCTGGCTCTTGAGATAactagtattatacctaggactgag
4-F	gatcgaagacacactagtCGTCGGTATCGTCACACCAGgttttagagctagaaatagcaagt
4-R	gatcgaagactaaaacCTGGTGTGACGATACCGACGactagtattatacctaggactgag
5-F	gatcgaagacacactagtAATATCAAACTTACTTGAGAgttttagagctagaaatagcaagt
5-R	gatcgaagactaaaacTCTCAAGTAAGTTTGATATTactagtattatacctaggactgag
6-F	gatcgaagacacactagtGACGCAGAAAATACCTGAATgttttagagctagaaatagcaagt
6-R	gatcgaagactaaaacATTCAGGTATTTTCTGCGTCactagtattatacctaggactgag
7-F	gatcgaagacacactagtCATAGTGATCAATCAAGGTAgttttagagctagaaatagcaagt
7-R	gatcgaagactaaaacTACCTTGATTGATCACTATGactagtattatacctaggactgag
8-F	gatcgaagacacactagtGCTAACAATGTGAGGAAAATgttttagagctagaaatagcaagt
8-R	gatcgaagactaaaacATTTTCCTCACATTGTTAGCactagtattatacctaggactgag
9-F	gatcgaagacacactagtAAGAATGGCCTTGTTTTCATgttttagagctagaaatagcaagt
9-R	gatcgaagactaaaacATGAAAACAAGGCCATTCTTactagtattatacctaggactgag
10-F	gatcgaagacacactagtAGAATTAACTTTGCTTTGAGgttttagagctagaaatagcaagt
10-R	gatcgaagactaaaacCTCAAAGCAAAGTTAATTCTactagtattatacctaggactgag
11-F	gatcgaagacacactagtTGCCTTGCTCTGGGTGGGACgttttagagctagaaatagcaagt
11-R	gatcgaagactaaaacGTCCCACCCAGAGCAAGGCAactagtattatacctaggactgag

12-F	gatcgaagacacactagtTGATTGAGCCAATCAATGGTgttttagagctagaaatagcaagt
12-R	gatcgaagactaaaacACCATTGATTGGCTCAATCAactagtattatacctaggactgag
13-F	gatcgaagacacactagtCTTTTACGGTATGAAACCCGgttttagagctagaaatagcaagt
13-R	gatcgaagactaaaacCGGGTTTCATACCGTAAAAGactagtattatacctaggactgag
14-F	gatcgaagacacactagtGATTCTCTACGGAATCTAAAgttttagagctagaaatagcaagt
14-R	gatcgaagactaaaacTTTAGATTCCGTAGAGAATCactagtattatacctaggactgag
15-F	gatcgaagacacactagtAAGATCGCAGTAATGGTAAGgttttagagctagaaatagcaagt
15-R	gatcgaagactaaaacCTTACCATTACTGCGATCTTactagtattatacctaggactgag
16-F	gatcgaagacacactagtTTGGCAGTTTGATGCGTGATgttttagagctagaaatagcaagt
16-R	gatcgaagactaaaacATCACGCATCAAACTGCCAAactagtattatacctaggactgag
17-F	gatcgaagacacactagtTAGATGGGCAAGAAAGACCAgttttagagctagaaatagcaagt
17-R	gatcgaagactaaaacTGGTCTTTCTTGCCCATCTAactagtattatacctaggactgag
18-F	gatcgaagacacactagtTAGATTGTGTCAGTGAACTTgttttagagctagaaatagcaagt
18-R	gatcgaagactaaaacAAGTTCACTGACACAATCTAactagtattatacctaggactgag
19-F	gatcgaagacacactagtCTCTAAAGCCAAGTAAAAAAgttttagagctagaaatagcaagt
19-R	gatcgaagactaaaacTTTTTTACTTGGCTTTAGAGactagtattatacctaggactgag
20-F	gatcgaagacacactagtGAGTAGTTTTTGTTGAGCTTgttttagagctagaaatagcaagt
20-R	gatcgaagactaaaacAAGCTCAACAAAAACTACTCactagtattatacctaggactgag
21-F	gatcgaagacacactagtATACATTGGCTTGATGTGCAgttttagagctagaaatagcaagt
21-R	gatcgaagactaaaacTGCACATCAAGCCAATGTATactagtattatacctaggactgag
22-F	gatcgaagacacactagtTTAATACCGCCAACATAAGTgttttagagctagaaatagcaagt
22-R	gatcgaagactaaaacACTTATGTTGGCGGTATTAAactagtattatacctaggactgag
23-F	gatcgaagacacactagtTAAGTTCACGATCGGCTCACgttttagagctagaaatagcaagt
23-R	gatcgaagactaaaacGTGAGCCGATCGTGAACTTAactagtattatacctaggactgag
24-F	gatcgaagacacactagtGTGGCGATATTCCGAAGGGAgttttagagctagaaatagcaagt
24-R	gatcgaagactaaaacTCCCTTCGGAATATCGCCACactagtattatacctaggactgag
25-F	gatcgaagacacactagtTCGGTGGTTTGGCGCACGGTgttttagagctagaaatagcaagt
25-R	gatcgaagactaaaacACCGTGCGCCAAACCACCGAactagtattatacctaggactgag
26-F	gatcgaagacacactagtGGCTAGCTAGCTCTTTATGCgttttagagctagaaatagcaagt
26-R	gatcgaagactaaaacGCATAAAGAGCTAGCTAGCCactagtattatacctaggactgag
27-F	gatcgaagacacactagtCGGCTGTAGAGCCAAACAGTgttttagagctagaaatagcaagt
27-R	gatcgaagactaaaacACTGTTTGGCTCTACAGCCGactagtattatacctaggactgag
28-F	gatcgaagacacactagtGCGATGCTTAGGCGCATAACgttttagagctagaaatagcaagt
28-R	gatcgaagactaaaacGTTATGCGCCTAAGCATCGCactagtattatacctaggactgag
29-F	gatcgaagacacactagtTCATGACCCCAAAACCCCAAgttttagagctagaaatagcaagt
29-R	gatcgaagactaaaacTTGGGGTTTTGGGGTCATGAactagtattatacctaggactgag
30-F	gatcgaagacacactagtGTAAACGTATCTATTCTTTCgttttagagctagaaatagcaagt

30-R	gatcgaagactaaaacGAAAGAATAGATACGTTTACactagtattatacctaggactgag
31-F	gatcgaagacacactagtATAACACCAACGATGACCTTgttttagagctagaaatagcaagt
31-R	gatcgaagactaaaacAAGGTCATCGTTGGTGTTATactagtattatacctaggactgag
32-F	gatcgaagacacactagtTGTCTCGCCTCAATCATGGCgttttagagctagaaatagcaagt
32-R	gatcgaagactaaaacGCCATGATTGAGGCGAGACAactagtattatacctaggactgag
33-F	gatcgaagacacactagtAAAAATCCAAAATATCGTTGgttttagagctagaaatagcaagt
33-R	gatcgaagactaaaacCAACGATATTTTGGATTTTTactagtattatacctaggactgag
34-F	gatcgaagacacactagtCTTCATTCCAATCAACGTGTgttttagagctagaaatagcaagt
34-R	gatcgaagactaaaacACACGTTGATTGGAATGAAGactagtattatacctaggactgag
35-F	gatcgaagacacactagtATCGTAAGCGACGTTAACGGgttttagagctagaaatagcaagt
35-R	gatcgaagactaaaacCCGTTAACGTCGCTTACGATactagtattatacctaggactgag
**Primers for targeting glycine pathway**	
1-F	gatcgaagacacactagtTGGGAGGCAAATCGAGTCTCgttttagagctagaaatagcaagt
1-R	gatcgaagactaaaacGAGACTCGATTTGCCTCCCAactagtattatacctaggactgag
2-F	gatcgaagacacactagtAATGCAATAAAACTGCTGAGgttttagagctagaaatagcaagt
2-R	gatcgaagactaaaacCTCAGCAGTTTTATTGCATTactagtattatacctaggactgag
3-F	gatcgaagacacactagtATCAAAAACATACAAAGGTTgttttagagctagaaatagcaagt
3-R	gatcgaagactaaaacAACCTTTGTATGTTTTTGATactagtattatacctaggactgag
4-F	gatcgaagacacactagtCCTACAGTGTGTGAGCTTGAgttttagagctagaaatagcaagt
4-R	gatcgaagactaaaacTCAAGCTCACACACTGTAGGactagtattatacctaggactgag
5-F	gatcgaagacacactagtCCATTAGTGTGGGAGCTGGAgttttagagctagaaatagcaagt
5-R	gatcgaagactaaaacTCCAGCTCCCACACTAATGGactagtattatacctaggactgag
6-F	gatcgaagacacactagtAAGTGGTAACCCGCGATCATgttttagagctagaaatagcaagt
6-R	gatcgaagactaaaacATGATCGCGGGTTACCACTTactagtattatacctaggactgag
7-F	gatcgaagacacactagtAAACAGGTGCGCGAAGGATTgttttagagctagaaatagcaagt
7-R	gatcgaagactaaaacAATCCTTCGCGCACCTGTTTactagtattatacctaggactgag
8-F	gatcgaagacacactagtCATGATTTTGTAAGACTGCTgttttagagctagaaatagcaagt
8-R	gatcgaagactaaaacAGCAGTCTTACAAAATCATGactagtattatacctaggactgag
9-F	gatcgaagacacactagtGACCAGGTCCCATTAAAATAgttttagagctagaaatagcaagt
9-R	gatcgaagactaaaacTATTTTAATGGGACCTGGTCactagtattatacctaggactgag
10-F	gatcgaagacacactagtCCTGGCGAAGAGTTTCTTCCgttttagagctagaaatagcaagt
10-R	gatcgaagactaaaacGGAAGAAACTCTTCGCCAGGactagtattatacctaggactgag
11-F	gatcgaagacacactagtATCATCTGTCGCAGAGAGGTgttttagagctagaaatagcaagt
11-R	gatcgaagactaaaacACCTCTCTGCGACAGATGATactagtattatacctaggactgag
12-F	gatcgaagacacactagtCAGATTTGTTTGGTCCATTGgttttagagctagaaatagcaagt
12-R	gatcgaagactaaaacCAATGGACCAAACAAATCTGactagtattatacctaggactgag
13-F	gatcgaagacacactagtGGCTTCTCGCATGGCTTGGGgttttagagctagaaatagcaagt

13-R	gatcgaagactaaaacCCCAAGCCATGCGAGAAGCCactagtattatacctaggactgag
14-F	gatcgaagacacactagtCGATTTGCTGCTGAATCTGTgttttagagctagaaatagcaagt
14-R	gatcgaagactaaaacACAGATTCAGCAGCAAATCGactagtattatacctaggactgag
15-F	gatcgaagacacactagtTGACCAACAACCATAGGCACgttttagagctagaaatagcaagt
15-R	gatcgaagactaaaacGTGCCTATGGTTGTTGGTCAactagtattatacctaggactgag
16-F	gatcgaagacacactagtGTAATTCCGATGGGAAAAATgttttagagctagaaatagcaagt
16-R	gatcgaagactaaaacATTTTTCCCATCGGAATTACactagtattatacctaggactgag
17-F	gatcgaagacacactagtTGCTCTAGTTGCTCCAGCGCgttttagagctagaaatagcaagt
17-R	gatcgaagactaaaacGCGCTGGAGCAACTAGAGCAactagtattatacctaggactgag
18-F	gatcgaagacacactagtACCAAAGTATCCTTCTTCGTgttttagagctagaaatagcaagt
18-R	gatcgaagactaaaacACGAAGAAGGATACTTTGGTactagtattatacctaggactgag
19-F	gatcgaagacacactagtTAGTTTTGGTGTAGGTAATTgttttagagctagaaatagcaagt
19-R	gatcgaagactaaaacAATTACCTACACCAAAACTAactagtattatacctaggactgag
20-F	gatcgaagacacactagtTCAACGTGAAGTGCGTGTAGgttttagagctagaaatagcaagt
20-R	gatcgaagactaaaacCTACACGCACTTCACGTTGAactagtattatacctaggactgag
21-F	gatcgaagacacactagtCGTTGTCACGTACCCACTCGgttttagagctagaaatagcaagt
21-R	gatcgaagactaaaacCGAGTGGGTACGTGACAACGactagtattatacctaggactgag
22-F	gatcgaagacacactagtCAGAACCAAGTACAACAACTgttttagagctagaaatagcaagt
22-R	gatcgaagactaaaacAGTTGTTGTACTTGGTTCTGactagtattatacctaggactgag
23-F	gatcgaagacacactagtGGTGGAGTCCCTCTAACAAGgttttagagctagaaatagcaagt
23-R	gatcgaagactaaaacCTTGTTAGAGGGACTCCACCactagtattatacctaggactgag
24-F	gatcgaagacacactagtAAGATCACGTAAATCCTGCTgttttagagctagaaatagcaagt
24-R	gatcgaagactaaaacAGCAGGATTTACGTGATCTTactagtattatacctaggactgag

Table S2. Strains and plasmids used in this work

Strains	Description	Source
E. coli DH5α	Cloning host	Invitroge
Vibrio alginolyticus FA2	Wild type	Stored in lab
**Plasmid**		
pdCas9	dCas9 expression cassette, CmR, p15A	Addgene#44249
pE2a-sgRNA	sgRNA expression plasmid, TetR, colE1	This study
pE2a-2sgRNA	dual-sgRNA cassettes, TetR, colE1	This study
pE2a-tool	dual-sgRNA library construction tool plasmid, TetR, colE1	This study
pE2a-mRFP-sgRNA	mRFP expression cassette, TetR, colE1	This study
pE2a-mRFP-eGFP-sgRNA	mRFP and eGFP expression cassette, TetR, colE1	This study
pGRS-pTrc-eGFP	glycine riboswitch and eGFP cassette, TetR, colE1	This study
pGRS-pTrc-Del-eGFP	glycine riboswitch without aptamer and eGFP cassette	This study
pGRS-J23100-eGFP	Promoter J23100 of glycine riboswitch and eGFP cassette	This study
pGRS-J23100-Del-eGFP	Promoter J23100 of glycine riboswitch without aptamer	This study
pGRS-Native-eGFP	Native promoter of glycine riboswitch and eGFP cassette	This study
pGRS-Native-Del-eGFP	Native promoter of glycine riboswitch without aptamer	This study
dCas9-pGRS-eGFP	dCas9 and glycine riboswitch expression cassette	This study

Table S3. Detailed sequences for constructed plasmids in this study.

>The dcas9 protein peptide sequence	MDKKYSIGLA IGTNSVGWAV ITDEYKVPSK KFKVLGNTDR HSIKKNLIGA<br>LLFDSGETAE ATRLKRTARR RYTRRKNRIC YLQEIFSNEM AKVDDSFFHR<br>LEESFLVEED KKHERHPIFG NIVDEVAYHE KYPTIYHLRK KLVDSTDKAD<br>LRLIYLALAH MIKFRGHFLI EGDLNPDNSD VDKLFIQLVQ TYNQLFEENP<br>INASGVDAKA ILSARLSKSR RLENLIAQLP GEKKNGLFGN LIALSLGLTP<br>NFKSNFDLAE DAKLQLSKDT YDDDLDNLLA QIGDQYADLF LAAKNLSDAI<br>LLSDILRVNT EITKAPLSAS MIKRYDEHHQ DLTLLKALVR QQLPEKYKEI<br>FFDQSKNGYA GYIDGGASQE EFYKFIKPIL EKMDGTEELL VKLNREDLLR<br>KQRTFDNGSI PHQIHLGELH AILRRQEDFY PFLKDNREKI EKILTFRIPY<br>YVGPLARGNS RFAWMTRKSE ETITPWNFEE VVDKGASAQS FIERMTNFDK<br>NLPNEKVLPK HSLLYEYFTV YNELTKVKYV TEGMRKPAFL SGEQKKAIVD<br>LLFKTNRKVT VKQLKEDYFK KIECFDSVEI SGVEDRFNAS LGTYHDLLKI<br>IKDKDFLDNE ENEDILEDIV LTLTLFEDRE MIEERLKTYA HLFDDKVMKQ<br>LKRRRYTGWG RLSRKLINGI RDKQSGKTIL DFLKSDGFAN RNFMQLIHDD<br>SLTFKEDIQK AQVSGQGDSL HEHIANLAGS PAIKKGILQT VKVVDELVKV<br>MGRHKPENIV IEMARENQTT QKGQKNSRER MKRIEEGIKE LGSQILKEHP<br>VENTQLQNEK LYLYYLQNGR DMYVDQELDI NRLSDYDVDA IVPQSFLKDD<br>SIDNKVLTRS DKNRGKSDNV PSEEVVKKMK NYWRQLLNAK LITQRKFDNL<br>TKAERGGLSE LDKAGFIKRQ LVETRQITKH VAQILDSRMN TKYDENDKLI<br>REVKVITLKS KLVSDFRKDF QFYKVREINN YHHAHDAYLN AVVGTALIKK<br>YPKLESEFVY GDYKVYDVRK MIAKSEQEIG KATAKYFFYS NIMNFFKTEI<br>TLANGEIRKR PLIETNGETG EIVWDKGRDF ATVRKVLSMP QVNIVKKTEV<br>QTGGFSKESI LPKRNSDKLI ARKKDWDPKK YGGFDSPTVA YSVLVVAKVE<br>KGKSKKLKSV KELLGITIME RSSFEKNPID FLEAKGYKEV KKDLIIKLPK<br>YSLFELENGR KRMLASAGEL QKGNELALPS KYVNFLYLAS HYEKLKGSPE<br>DNEQKQLFVE QHKHYLDEII EQISEFSKRV ILADANLDKV LSAYNKHRDK<br>PIREQAENII HLFTLTNLGA PAAFKYFDTT IDRKRYTSTK EVLDATLIHQ<br>SITGLYETRIDLSQLGGD*
>The sgRNA library design<br>N_i: 1st N20 matching region<br>N_j: 2nd N20 matching region	TTGACAGCTAGCTCAGTCCTAGGTATAATACTAGTN_iGTTTTAGAGCTAGAA<br>ATAGCAAGTTAAAATAAGGCTAGTCCGTTATCAACTTGAAAAAGTGGCACC<br>GAGTCGGTGCCTAGTGCTTGGATTCTCACCAATAAAAAACGCCCGGCGGCA<br>ACCGAGCGTTCTGAACAAATCCAGATGGAGTTCTGAGGTCATTACTGGATCT<br>ATCAACAGGAGTCCAAGCTTGACAGCTAGCTCAGTCCTAGGTATAATACTA<br>GTN_jGTTTTAGAGCTAGAAATAGCAAGTTAAAATAAGGCTAGTCCGTTATCA<br>ACTTGAAAAAGTGGCACCGAGTCGGTGCCTAGTGCTTGGATTCTCACCAATA

	AAAAACGCCCGGCGGCAACCGAGCGTTCTGAACAAATCCAGATGGAGTTCTGAGGTCATTACTGGATCTATCAACAGGAGTC
>The plasmid sequence of pGRS-eGFP<br><br>Purple: sequence of the pTrc promoter<br><br>Blue: sequence of the glycine riboswitch element<br><br>Wave-underlined: aptamer of glycine riboswitch<br><br>Green: eGFP sequence	CGACTGCACGGTGCACCAATGCTTCTGGCGTCAGGCAGCCATCGGAAGCTGTGGTATGGCTGTGCAGGTCGTAAATCACTGCATAATTCGTGTCGCTCAAGGCGCACTCCCGTTCTGGATAATGTTTTTTGCGCCGACATCATAACGGTTCTGGCAAATATTCTGAAATGAGCTGTTGACAATTAATCATCCGGCTCGTATAATGTGTGGAATTGTGAGCGGATAACAATTTCAGAATTCAAAAGATCTGTTGAAGACTGCAGGAGAGTGGTTGTTAACCAGATTTTAACATCTGAGCCAAATAACCCGCCGAAGAAGTAAATCTTTCAGGTGCATTATTCTTAGCCATATATTGGCAACGAATAAGCGAGGACTGTAGTTGGAGGAACCTCTGGAGAGAACCGTTTAATCGGTCGCCGAAGGAGCAAGCTCTGCGCATATGCAGAGTGAAACTCTCAGGCAAAAGGACAGAGGAGTGAAAGGCCAATCTTTTAGTGAGCTCGCTAGAGCTCTGCTGTGCATTTTTCGCACCCTTTCTCTCTTCTCCTTATGTTTATTTTTTAAGGGGAAATCATGAATAACCTGCAATCTTTTCTGCAAATGGTTTCTAAAGGTGAAGAATTGTTTACTGGTGTTGTTCCAATTTTGGTTGAATTGGATGGTGATGTTAACGGTCACAAATTTTCTGTTTCTGGTGAAGGTGAAGGTGATGCAACTTACGGTAAATTGACTTTGAAATTTATTTGTACTACTGGTAAATTGCCTGTTCCATGGCCAACTTTGGTTACTACTTTGACTTACGGTGTTCAATGTTTTTCTCGTTACCCTGATCACATGAAACAACACGATTTTTTTAAATCTGCAATGCCTGAAGGTTACGTTCAAGAACGTACTATTTTTTTTAAAGATGATGGTAACTACAAAACTCGTGCAGAAGTTAAATTTGAAGGTGATACTTTGGTTAACCGTATTGAATTGAAAGGTATTGATTTTAAAGAAGATGGTAACATTTTGGGTCACAAATTGGAATACAACTACAACTCTCACAACGTTTACATTATGGCAGATAAACAAAAAAATGGCATTAAAGTTAACTTTAAAATTCGTCACAACATTGAAGATGGTTCTGTTCAATTGGCAGATCACTACCAACAAAACACTCCAATTGGTGATGGTCCTGTTTTGTTGCCTGATAACCACTACTTGTCTACTCAATCTGCATTGTCTAAAGATCCAAACGAAAAACGTGATCACATGGTTTTGTTGGAATTTGTTACTGCAGCGGGTATTACTTTGGGTATGGATGAATTGTACAAATAA
> The plasmid sequence of	CGACTGCACGGTGCACCAATGCTTCTGGCGTCAGGCAGCCATCGGAAGCTGTGGTATGGCTGTGCAGGTCGTAAATCACTGCATAATTCGTGTCGCTCAAGGCGCACTCCCGTTCTGGATAATGTTTTTTGCGCCGACATCATAACGGTTCTGGCAAATATTCTGAAATGAGCTGTTGACAATTAATCATCCGGCTCGTATAATGTGTGGAATTGTGAGCGGATAACAATTTCAGAATTCAAAAGATCTGTGAAAGGCCAATCTTTTAGTGAGCTCGCTAGAGCTCTGCTGTGCATTTTTCGCACCCTTTCTCTCTTCTCCTTATGTTTATTTTTTAAGGGGAAATCATGAATAACCTGCAATCTTTTCTGCAAATGGTTTCTAAAGGTGAAGAATTGTTTACTGGTGTTGTTCCA

	ATTTTGGTTGAATTGGATGGTGATGTTAACGGTCACAAATTTTCTGTTTCTGG<br>TGAAGGTGAAGGTGATGCAACTTACGGTAAATTGACTTTGAAATTTATTTGT<br>ACTACTGGTAAATTGCCTGTTCCATGGCCAACTTTGGTTACTACTTTGACTTA<br>CGGTGTTCAATGTTTTTCTCGTTACCCTGATCACATGAAACAACACGATTTTT<br>TTAAATCTGCAATGCCTGAAGGTTACGTTCAAGAACGTACTATTTTTTTAA<br>AGATGATGGTAACTACAAAACTCGTGCAGAAGTTAAATTTGAAGGTGATAC<br>TTTGGTTAACCGTATTGAATTGAAAGGTATTGATTTTAAAGAAGATGGTAAC<br>ATTTTGGGTCACAAATTGGAATACAACTACAACTCTCACAACGTTTACATTA<br>TGGCAGATAAACAAAAAAATGGCATTAAAGTTAACTTTAAAATTCGTCACA<br>ACATTGAAGATGGTTCTGTTCAATTGGCAGATCACTACCAACAAAACACTCC<br>AATTGGTGATGGTCCTGTTTTGTTGCCTGATAACCACTACTTGTCTACTCAAT<br>CTGCATTGTCTAAAGATCCAAACGAAAAACGTGATCACATGGTTTTGTTGGA<br>ATTTGTTACTGCAGCGGGTATTACTTTGGGTATGGATGAATTGTACAAATAA
> The plasmid sequence of pGRS-J23100-eGFP<br><br>Red: sequence of the J23100 promoter	TTGACGGCTAGCTCAGTCCTAGGTACAGTGCTAGCAATATTCAGATGAAGTA<br>TTAGATGAAAGTTGAAGACTGCAGGAGAGTGGTTGTTAACCAGATTTTAAC<br>ATCTGAGCCAAATAACCCGCCGAAGAAGTAAATCTTTCAGGTGCATTATTCT<br>TAGCCATATATTGGCAACGAATAAGCGAGGACTGTAGTTGGAGGAACCTCT<br>GGAGAGAACCGTTTAATCGGTCGCCGAAGGAGCAAGCTCTGCGCATATGCA<br>GAGTGAAACTCTCAGGCAAAAGGACAGAGGAGTGAAAGGCCAATCTTTTAG<br>TGAGCTCGCTAGAGCTCTGCTGTGCATTTTTCGCACCCTTTCTCTCTTCTCCT<br>TATGTTTATTTTTTAAGGGGAAATCATGAATAACCTGCAATCTTTTCTGCAA<br>ATGGTTTCTAAAGGTGAAGAATTGTTTACTGGTGTTGTTCCAATTTTGGTTG<br>AATTGGATGGTGATGTTAACGGTCACAAATTTTCTGTTTCTGGTGAAGGTGA<br>AGGTGATGCAACTTACGGTAAATTGACTTTGAAATTTATTTGTACTACTGGT<br>AAATTGCCTGTTCCATGGCCAACTTTGGTTACTACTTTGACTTACGGTGTTCA<br>ATGTTTTTCTCGTTACCCTGATCACATGAAACAACACGATTTTTTAAATCTG<br>CAATGCCTGAAGGTTACGTTCAAGAACGTACTATTTTTTTAAAGATGATGG<br>TAACTACAAAACTCGTGCAGAAGTTAAATTTGAAGGTGATACTTTGGTTAAC<br>CGTATTGAATTGAAAGGTATTGATTTTAAAGAAGATGGTAACATTTTGGGTC<br>ACAAATTGG<br>AATACAACTACAACTCTCACAACGTTTACATTATGGCAGATAAACAAAAAA<br>ATGGCATTAAAGTTAACTTTAAAATTCGTCACAACATTGAAGATGGTTCTGT<br>TCAATTGGCAGATCACTACCAACAAAACACTCCAATTGGTGATGGTCCTGTT<br>TTGTTGCCTGATAACCACTACTTGTCTACTCAATCTGCATTGTCTAAAGATCC<br>AAACGAAAAACGTGATCACATGGTTTTGTTGGAATTTGTTACTGCAGCGGGT<br>ATTACTTTGGGTATGGATGAATTGTACAAATAA

> The plasmid sequence of pGRS-J23100-Del-eGFP	TTGACGGCTAGCTCAGTCCTAGGTACAGTGCTAGCAATATTCAGATGAAGTA<br>TTAGATGAAAGTGAAAGGCCAATCTTTTAGTGAGCTCGCTAGAGCTCTGCTG<br>TGCATTTTTCGCACCCTTTCTCTCTTCTCCTTATGTTTATTTTTTAAGGGGAAA<br>TCATGAATAACCTGCAATCTTTTCTGCAAATGGTTTCTAAAGGTGAAGAATT<br>GTTTACTGGTGTTGTTCCAATTTTGGTTGAATTGGATGGTGATGTTAACGGTC<br>ACAAATTTTCTGTTTCTGGTGAAGGTGAAGGTGATGCAACTTACGGTAAATT<br>GACTTTGAAATTTATTTGTACTACTGGTAAATTGCCTGTTCCATGGCCAACTT<br>TGGTTACTACTTTGACTTACGGTGTTCAATGTTTTTCTCGTTACCCTGATCAC<br>ATGAAACAACACGATTTTTTTAAATCTGCAATGCCTGAAGGTTACGTTCAAG<br>AACGTACTATTTTTTTTAAAGATGATGGTAACTACAAAACTCGTGCAGAAGT<br>TAAATTTGAAGGTGATACTTTGGTTAACCGTATTGAATTGAAAGGTATTGAT<br>TTTAAAGAAGATGGTAACATTTTGGGTCACAAATTGG<br>AATACAACTACAACTCTCACAACGTTTACATTATGGCAGATAAACAAAAAA<br>ATGGCATTAAAGTTAACTTTAAAATTCGTCACAACATTGAAGATGGTTCTGT<br>TCAATTGGCAGATCACTACCAACAAAACACTCCAATTGGTGATGGTCCTGTT<br>TTGTTGCCTGATAACCACTACTTGTCTACTCAATCTGCATTGTCTAAAGATCC<br>AAACGAAAAACGTGATCACATGGTTTTGTTGGAATTTGTTACTGCAGCGGGT<br>ATTACTTTGGGTATGGATGAATTGTACAAATAA
> The plasmid sequence of pGRS-Native-eGFP<br><br>Yellow: sequence of the native promoter	CATTGGTACGGTAGCGAATCAACACAAACGCTAATGCTATCAGTAAGGCTA<br>TTGCAATTAATCTCACCATCTCCCGCCTCCAAGCGTGATTATCTTATAACACT<br>TATACGTAGTTTAACGTCAGAGAAAAGTGGATTAGCAAAAGACTTGCGCAA<br>CATTTCATCAGTTAATTTCTTTTGTAGGTGACATCACATTTTCTTTTCGCTAG<br>TATCCGCCTTGCAAATCGTTTTATTCAAGACGATTGTTCCGTTGAAGACTGC<br>AGGAGAGTGGTTGTTAACCAGATTTTAACATCTGAGCCAAATAACCCGCCG<br>AAGAAGTAAATCTTTCAGGTGCATTATTCTTAGCCATATATTGGCAACGAAT<br>AAGCGAGGACTGTAGTTGGAGGAACCTCTGGAGAGAACCGTTTAATCGGTC<br>GCCGAAGGAGCAAGCTCTGCGCATATGCAGAGTGAAACTCTCAGGCAAAAG<br>GACAGAGGAGTGAAAGGCCAATCTTTTAGTGAGCTCGCTAGAGCTCTGCTG<br>TGCATTTTTCGCACCCTTTCTCTCTTCTCCTTATGTTTATTTTTTAAGGGGAAA<br>TCATGAATAACCTGCAATCTTTTCTGCAAATGGTTTCTAAAGGTGAAGAATT<br>GTTTACTGGTGTTGTTCCAATTTTGGTTGAATTGGATGGTGATGTTAACGGTC<br>ACAAATTTTCTGTTTCTGGTGAAGGTGAAGGTGATGCAACTTACGGTAAATT<br>GACTTTGAAATTTATTTGTACTACTGGTAAATTGCCTGTTCCATGGCCAACTT<br>TGGTTACTACTTTGACTTACGGTGTTCAATGTTTTTCTCGTTACCCTGATCAC<br>ATGAAACAACACGATTTTTTTAAATCTGCAATGCCTGAAGGTTACGTTCAAG<br>AACGTACTATTTTTTTTAAAGATGATGGTAACTACAAAACTCGTGCAGAAGT<br>TAAATTTGAAGGTGATACTTTGGTTAACCGTATTGAATTGAAAGGTATTGAT

	TTTAAAGAAGATGGTAACATTTTGGGTCACAAATTGGAATACAACTACAAC<br>TCTCACAACGTTTACATTATGGCAGATAAACAAAAAAATGGCATTAAAGTT<br>AACTTTAAAATTCGTCACAACATTGAAGATGGTTCTGTTCAATTGGCAGATC<br>ACTACCAACAAAACACTCCAATTGGTGATGGTCCTGTTTTGTTGCCTGATAA<br>CCACTACTTGTCTACTCAATCTGCATTGTCTAAAGATCCAAACGAAAAACGT<br>GATCACATGGTTTTGTTGGAATTTGTTACTGCAGCGGGTATTACTTTGGGTA<br>TGGATGAATTGTACAAATAA
> The plasmid sequence of pGRS-Native-Del-eGFP	CATTGGTACGGTAGCGAATCAACACAAACGCTAATGCTATCAGTAAGGCTA<br>TTGCAATTAATCTCACCATCTCCCGCCTCCAAGCGTGATTATCTTATAACACT<br>TATACGTAGTTTAACGTCAGAGAAAAGTGGATTAGCAAAAGACTTGCGCAA<br>CATTTCATCAGTTAATTTCTTTTGTAGGTGACATCACATTTTCTTTTCGCTAG<br>TATCCGCCTTGCAAATCGTTTTATTCAAGACGATTGTTCCGTGAAAGGCCAA<br>TCTTTTAGTGAGCTCGCTAGAGCTCTGCTGTGCATTTTTCGCACCCTTTCTCT<br>CTTCTCCTTATGTTTATTTTTTAAGGGGAAATCATGAATAACCTGCAATCTTT<br>TCTGCAAATGGTTTCTAAAGGTGAAGAATTGTTTACTGGTGTTGTTCCAATT<br>TTGGTTGAATTGGATGGTGATGTTAACGGTCACAAATTTTCTGTTTCTGGTG<br>AAGGTGAAGGTGATGCAACTTACGGTAAATTGACTTTGAAATTTATTTGTAC<br>TACTGGTAAATTGCCTGTTCCATGGCCAACTTTGGTTACTACTTTGACTTACG<br>GTGTTCAATGTTTTTCTCGTTACCCTGATCACATGAAACAACACGATTTTTTT<br>AAATCTGCAATGCCTGAAGGTTACGTTCAAGAACGTACTATTTTTTTTAAAG<br>ATGATGGTAACTACAAAACTCGTGCAGAAGTTAAATTTGAAGGTGATACTTT<br>GGTTAACCGTATTGAATTGAAAGGTATTGATTTTAAAGAAGATGGTAACATT<br>TTGGGTCACAAATTGGAATACAACTACAACTCTCACAACGTTTACATTATGG<br>CAGATAAACAAAAAAATGGCATTAAAGTTAACTTTAAAATTCGTCACAACA<br>TTGAAGATGGTTCTGTTCAATTGGCAGATCACTACCAACAAAACACTCCAAT<br>TGGTGATGGTCCTGTTTTGTTGCCTGATAACCACTACTTGTCTACTCAATCTG<br>CATTGTCTAAAGATCCAAACGAAAAACGTGATCACATGGTTTTGTTGGAATT<br>TGTTACTGCAGCGGGTATTACTTTGGGTATGGATGAATTGTACAAATAA
>The pE2A plasmid sequence<br><br>Red: the sequence of *ccdB* gene	TTGACAGCTAGCTCAGTCCTAGGTATAATACTAGTGTCTTCGATCATGCAGT<br>TCAAGGTGTACACCTACAAGCGTGAGTCGCGTTACCGTCTGTTCGTCGATGT<br>GCAGTCGGACATCATCGACACGCCCGGCCGTCGTATGGTCATCCCGCTGGCC<br>AGCGCCCGTCTGCTGTCGGACAAGGTCAGCCGCGAGCTGTACCCGGTGGTG<br>CACGTGGGCGACGAGAGCTGGCGCATGATGACCACCGACATGGCCTCGGTC<br>CCGGTCAGCGTCATCGGCGAGGAGGTGGCCGACCTGTCGCACCGTGAGAAC<br>GACATCAAGAACGCCATCAACCTGATGTTCTGGGGCATCTGAGATCGAAGA<br>CGTGTTTTAGAGCTAGAAATAGCAAGTTAAAATAAGGCTAGTCCGTTATCA

	ACTTGAAAAAGTGGCACCGAGTCGGTGCCTAGTGCTTGGATTCTCACCAATA<br>AAAAACGCCCGGCGGCAACCGAGCGTTCTGAACAAATCCAGATGGAGTTCT<br>GAGGTCATTACTGGATCTATCAACAGGAGTCCAAGC

Table S4. Details of the target histidine kinase genes.

NO.	Name	Library	EC.number	Function
1	*btsS*	Library I	2.7.13.3	Sensor histidine kinase YehU
2	*dctB_1*	Library I	2.7.13.3	Signal transduction histidine kinase regulating C4-dicarboxylate transport system
3	*rcsC_1*	Library I	2.7.13.3	Sensor histidine kinase
4	*sasA_1*	Library I	2.7.13.3	Sensor histidine kinase PhoQ
5	*sasA_2*	Library I	2.7.13.3	Sensor histidine kinase
6	*sasA_3*	Library I	2.7.13.3	Sensor histidine kinase
7	HDDFHODA_01099	Library I	2.7.13.3	Signal transduction histidine kinase SypF
8	*sasA_4*	Library I	2.7.13.3	Signal transduction histidine kinase
9	*rcsC_3*	Library I	2.7.13.3	Signal transduction histidine kinase
10	HDDFHODA_01507	Library I	2.7.13.3	Sensor histidine kinase
11	HDDFHODA_01586	Library I	2.7.13.3	Two-component system sensor histidine kinase
12	*ttrS*	Library I	2.7.13.3	Tetrathionate reductase sensory transduction histidine kinase
13	HDDFHODA_01805	Library I	2.7.13.3	Signal transduction histidine kinase CheA
14	*atoS*	Library I	2.7.13.3	Flagellar sensor histidine kinase FleS
15	*rcsC_5*	Library I	2.7.13.3	Chitin catabolic cascade sensor histidine kinase ChiS
16	*cpxA*	Library I	2.7.13.3	Copper sensory histidine kinase CpxA
17	*sasA_5*	Library I	2.7.13.3	Sensor histidine kinase
18	*envZ*	Library I	2.7.13.3	Osmolarity sensory histidine kinase EnvZ
19	HDDFHODA_03340	Library I	2.7.13.3	Sensor histidine kinase
20	*sasA_6*	Library I	2.7.13.3	Signal transduction histidine kinase
21	*sasA_7*	Library I	2.7.13.3	Two-component system sensor histidine kinase
22	*rcsC_6*	Library I	2.7.13.3	Signal transduction histidine kinase
23	*dctB_2*	Library I	2.7.13.3	Signal transduction histidine kinase
24	*sasA_8*	Library I	2.7.13.3	Integral membrane sensor signal transduction histidine kinase, glucose catabolism cluster
25	*creC*	Library I	2.7.13.3	Sensory histidine kinase CreC of two-component signal transduction system

				CreBC
26	*sasA_9*	Library I	2.7.13.3	Sensor histidine kinase
27	*kdpD*	Library I	2.7.13.3	Osmosensitive K+ channel histidine kinase KdpD
28	*rcsC_7*	Library I	2.7.13.3	putative sensory box sensor histidine kinase/response regulator
29	HDDFHODA_04152	Library I	2.7.13.3	Two-component system sensor histidine kinase
30	HDDFHODA_04288	Library I	2.7.13.3	Signal transduction histidine kinase
31	*rcsC_8*	Library I	2.7.13.3	Sensory box sensor histidine kinase/response regulator VieS
32	*torS*	Library I	2.7.13.3	Trimethylamine-N-oxide sensor histidine kinase TorS
33	*rcsC_9*	Library I	2.7.13.3	Sensor histidine kinase
34	*sasA_10*	Library I	2.7.13.3	Signal transduction histidine kinase
35	*rcsC_11*	Library I	2.7.13.3	Sensory histidine kinase in two-component regulatory system with RstA

Table S5. Details of the target glycine pathway genes.

NO.	Name	Library	EC. number	Product
1	HDDFHODA_02026	Library II	3.1.3.3	Phosphoserine phosphatase
2	HDDFHODA_03430	Library II	3.1.3.3	Phosphoserine phosphatase
3	HDDFHODA_03190	Library II	3.1.3.3	Phosphoserine phosphatase
4	*sdaA*	Library II	4.3.1.17	L-serine dehydratase 1
5	*sdaB_1*	Library II	4.3.1.17	L-serine dehydratase 2
6	*sdaB_2*	Library II	4.3.1.17	L-serine dehydratase 2
7	*ilvA*	Library II	4.3.1.19	L-threonine dehydratase biosynthetic IlvA
8	*trpA*	Library II	4.2.1.20	Tryptophan synthase alpha chain
9	*pucG*	Library II	2.6.1.112	(*S*)-ureidoglycine--glyoxylate transaminase
10	*glyA*	Library II	2.1.2.1	Serine hydroxymethyltransferase
11	*glyA2*	Library II	2.1.2.1	Serine hydroxymethyltransferase 2
12	*gcvP*	Library II	1.4.4.2	Glycine dehydrogenase (decarboxylating)
13	*ltaE*	Library II	4.1.2.48	Low specificity L-threonine aldolase
14	*kbl*	Library II	2.3.1.29	2-amino-3-ketobutyrate coenzyme A ligase
15	*tdh_2*	Library II	1.1.1.103	L-threonine 3-dehydrogenase
16	*thrC*	Library II	4.2.3.1	Threonine synthase
17	*trpB_1*	Library II	4.2.1.20	Tryptophan synthase beta chain
18	*trpB_2*	Library II	4.2.1.20	Tryptophan synthase beta chain
19	*tdh_1*	Library II	1.1.1.103	L-threonine 3-dehydrogenase
20	*gcvT*	Library II	2.1.2.10	Aminomethyltransferase
21	*gcvH*	Library II	-	Glycine cleavage system H protein
22	*lpdA*	Library II	1.8.1.4	Dihydrolipoyl dehydrogenase
23	*serA*	Library II	1.1.1.95	D-3-phosphoglycerate dehydrogenase
24	*serC*	Library II	2.6.1.52	Phosphoserine aminotransferase

Table S6. Python3 programs used for to analyze the frequencies of dual-sgRNA cassettes from high-throughput sequencing.

> python3 extract-seq-fasta.py	#coding:utf-8<br><br>import argparse<br>import csv<br>import re<br>#<br>parser = argparse.ArgumentParser()<br>parser.add_argument("f1", help="Input a fastaq files of sequencing")<br>args = parser.parse_args()<br>f1 = args.f1<br><br>#fastaq<br>seqs={}<br>with open(f1,'r',encoding='UTF-8') as f:<br>lines = f.readlines()<br>id=''<br>i = 0<br>sq = ["",""]<br>for line in lines:<br>line = line.strip()<br>if(re.match(r'\A@',line)):<br>id = line<br>i = 0<br>sq = ["",""]<br>else:<br>if(re.match(r'\A\+',line)):<br>i = 1<br>else:<br>sq[i] += line<br>seqs[id] = sq<br>f2 = f1+"-out.fasta"<br>with open(f2,'w',encoding='UTF-8') as fout:<br>for ky in seqs:<br>seq = seqs[ky][0][47:67]+seqs[ky][0][289:309] + "\n"<br>id = ">" + ky.lstrip('@') + "\n"

	```<br>fout.write(id)<br>fout.write(seq)<br>```
> python3 ana_plot.py	```<br>#!/usr/bin/env python<br>#-*-coding:utf-8 -*-<br><br>import argparse<br>import numpy as np<br>import matplotlib.pyplot as plt<br>import re<br><br>parser = argparse.ArgumentParser()<br>parser.add_argument("file", help="Input a files containing data in sam format")<br>args = parser.parse_args()<br>file = args.file<br>fig_name = file+'-out.eps'<br>tab_name = file+'-out.tsv'<br><br>dic = dict()<br>reads_num = 0<br>num = 35<br>for line in open(file):<br>if not line.startswith('@'):<br>x = line.split('\t')<br>if not (x[2] == '*'):<br>reads_num += 1<br>tem = x[2].split('::')<br>if(num<int(tem[0])):<br>mum = int(tem[0])<br>if(num<int(tem[1])):<br>num = int(tem[1])<br>key_id = tem[0]+'_'+tem[1]<br>if(key_id in dic.keys()):<br>dic[key_id] += 1<br>else:<br>dic[key_id] = 1<br><br>max_hit_id = max(dic, key=dic.get)<br>```

	max_freq = float(dic[max_hit_id])/reads_num*10000<br><br>table = np.zeros((num,num))<br>for i in dic.keys():<br>x = int(i.split('_')[0])<br>y = int(i.split('_')[1])<br>z = float(dic[i])/reads_num*10000<br>table[x-1][y-1] = z<br>#cm = plt.cm.get_cmap('OrRd')<br>#cm = plt.cm.get_cmap('RdPu')<br>cm = plt.cm.get_cmap('YlOrRd')<br>sc = plt.scatter(x,y,c=z,vmin=0,vmax=300,s=40,cmap=cm,marker = 's')<br><br>fo = open(tab_name, "w")<br>for y in range(num-1,-1,-1):<br>fo.write(str(y+1))<br>fo.write("\t")<br>for x in range(num):<br>fo.write(str(table[x][y]))<br>fo.write("\t")<br>fo.write("\n")<br>fo.write("\t")<br>for i in range(num):<br>fo.write(str(i+1))<br>fo.write("\t")<br>fo.close()<br><br>font1 = {'family' : 'Times New Roman',<br>'weight' : 'normal',<br>'size' : 18,<br>}<br>cb = plt.colorbar(sc)<br>cb.set_label('Frequency (10^{-4})',fontsize = 12)<br><br>plt.xlim((0, 36))<br>plt.ylim((0, 36))

	#plt.tick_params(labelsize=12)<br>my_x_ticks = np.arange(1, 36, 2)<br>my_y_ticks = np.arange(1, 36, 2)<br>plt.xticks(my_x_ticks)<br>plt.yticks(my_y_ticks)<br><br>plt.xlabel("Position I",font1)<br>plt.ylabel("Position II",font1)<br><br>plt.savefig(fig_name)<br>plt.show()

Table S7. Python3 programs used for survivorship-based metabolic interaction analysis bioinformatical analysis (SMIA).

> cross-v2.py	<pre>#!/usr/bin/env python<br>#-*-coding:utf-8 -*-<br>import argparse<br>import numpy as np<br>import matplotlib<br>import matplotlib.pyplot as plt<br>import re<br>import pandas as pd<br>#<br>control = pd.read_csv("2-dui-3.fq-out.fasta-sam-out.tsv",sep='\t',header=None) #<br>sample = pd.read_csv("2-shi-3.fq-out.fasta-sam-out.tsv",sep='\t',header=None)<br>num = control.shape[0] - 1 #<br>#<br>control = control.drop(control.index[[num]])<br>control = control.drop(control.columns[[0,num+1]],axis=1)<br>sample = sample.drop(sample.index[[num]])<br>sample = sample.drop(sample.columns[[0,num+1]],axis=1)<br>#<br>con = control.to_numpy()<br>con = np.flipud(con)<br>sam = sample.to_numpy()<br>sam = np.flipud(sam)<br>delta = (sam+sam.T) - (con + con.T)<br>#<br>font1 = {'family' : 'Times New Roman',<br>'weight' : 'normal',<br>'size' : 12,<br>}<br>ticks_font = matplotlib.font_manager.FontProperties(family='times new roman', style='normal', size=10, weight='normal', stretch='normal')<br>#<br>fig1 = plt.figure('Figure1',figsize = (6,4),dpi=300).add_subplot(111) #<br>single = np.zeros((1,num))<br>for i in range(num):<br>single[0][i] = delta[i][i]/4</pre>

	<pre>x = range(1,num+1)<br>y = single[0]<br>colors = []<br>for n in range(num):<br>if(y[n]>0):<br>colors.append('red')<br>else:<br>colors.append('blue')<br><br>my_x_ticks = np.arange(1, num+1, 2)<br>fig1.set_xticks(my_x_ticks)<br>fig1.bar(x, y, color=colors)<br>#fig1.tick_params(labelsize=10)<br>for label in fig1.get_xticklabels():<br>label.set_fontproperties(ticks_font)<br>for label in fig1.get_yticklabels():<br>label.set_fontproperties(ticks_font)<br>fig1.set_xlabel("Target Gene",font1)<br>fig1.set_ylabel("ΔF$_{i}$ (10^{-4})",font1)<br>fig1.hlines(y=0.0, xmin=0, xmax=num+1, linewidth=0.5,<br>linestyles='dashed',colors='k')<br>plt.savefig("single.png",bbox_inches='tight', pad_inches=0.1)<br>#<br>with open("single.tsv",'w') as S:<br>for i in range(1,num+1):<br>S.write(str(i))<br>S.write('\t')<br>S.write('\n')<br>for i in range(num):<br>S.write(str(single[0][i]))<br>S.write('\t')<br>S.write('\n')<br># cross<br>cross = np.zeros((num,num))<br>for i in range(num):<br>for j in range(num):<br>if(i==j):</pre>

```
                cross[i][j] = delta[i][j]/2 - (single[0][i] + single[0][j])
            else:
                cross[i][j] = delta[i][j] - (single[0][i] + single[0][j])
#
fig2 = plt.figure('Figure2',figsize = (6,4),dpi=300).add_subplot(111) #
for i in range(num):
    for j in range(num):
        #if(j>i):
        #       continue
        x = i+1
        y = j+1
        z = cross[i][j]
        cm = plt.cm.get_cmap('bwr')
        if(cross[i][j] >0):
            sc = fig2.scatter(x,y,c=z,vmin=-
100,vmax=100,s=20,cmap=cm,marker = 'P')
        elif(cross[i][j]<0):
            sc = fig2.scatter(x,y,c=z,vmin=-
100,vmax=100,s=20,cmap=cm,marker = 'X')
        else:
            sc = fig2.scatter(x,y,c=z,vmin=-
100,vmax=100,s=20,cmap=cm,marker = 'o')
#
fig2.plot([1,num],[1,num],linestyle='--',c='k', linewidth=0.5)
#
cb = plt.colorbar(sc)
cb.set_label('Cross Index',fontsize=12)
cb.ax.tick_params(labelsize=8)
#
fig2.set_xlim([0, num+1])
fig2.set_ylim([0, num+1])

my_x_ticks = np.arange(1, num+1, 2)
my_y_ticks = np.arange(1, num+1, 2)
fig2.set_xticks(my_x_ticks)
fig2.set_yticks(my_y_ticks)
#fig2.tick_params(labelsize=10)
```

	```<br>for label in fig2.get_xticklabels():<br>label.set_fontproperties(ticks_font)<br>for label in fig2.get_yticklabels():<br>label.set_fontproperties(ticks_font)<br>#<br>fig2.set_xlabel("Target Gene",font1)<br>fig2.set_ylabel("Target Gene",font1)<br>#<br>plt.savefig("cross.png", bbox_inches='tight', pad_inches=0.1)<br>#tsv<br>with open("cross.tsv",'w') as C:<br>for i in range(num+1): #<br>if(i>0):<br>C.write(str(i))<br>C.write('\t')<br>C.write('\n')<br>for i in range(num): #<br>C.write(str(str(i+1))) #<br>C.write('\t')<br>for j in range(num):<br>C.write(str(cross[i][j]))<br>C.write('\t')<br>C.write('\n')<br>```

Table S8. Frequencies of dual-sgRNA cassettes in *E. coli* HK-L1 library.

35	0.323133	0.323133	0.969399	0.646266	0.323133	1.615665	0	0.969399	1.292532	0.646266	0.646266
34	12.27906	3.877597	3.554464	23.26558	2.585065	21.32678	2.261932	7.108928	5.17013	4.846996	10.01713
33	10.01713	2.585065	3.554464	22.94245	3.231331	21.32678	2.908198	6.139529	6.139529	2.585065	7.755194
32	14.86412	3.231331	4.20073	39.42224	6.785795	54.60949	5.493263	13.24846	14.54099	4.20073	12.27906
31	4.523863	0.969399	1.615665	11.30966	1.938799	12.60219	2.261932	2.908198	1.615665	1.615665	5.493263
30	3.877597	0.646266	2.585065	13.89472	2.261932	19.06485	1.615665	4.20073	2.261932	2.261932	5.816396
29	27.14318	8.724594	10.98653	76.58254	13.57159	98.5556	10.98653	24.88125	22.61932	6.785795	29.72825
28	9.693993	1.938799	2.908198	24.23498	4.20073	32.95958	1.292532	11.30966	10.66339	2.585065	14.54099
27	13.24846	1.938799	5.493263	30.05138	3.877597	40.71477	4.846996	15.51039	8.401461	4.846996	11.63279
26	4.846996	0.323133	2.585065	12.92532	1.292532	23.91185	2.908198	4.523863	4.523863	2.261932	8.078327
25	12.27906	1.938799	4.523863	34.57524	2.585065	37.16031	4.20073	10.98653	10.01713	3.877597	13.89472
24	16.80292	4.20073	4.846996	41.68417	5.493263	70.76615	3.877597	18.41859	13.24846	3.877597	15.51039
23	2.585065	1.615665	2.908198	12.27906	3.877597	18.09545	2.585065	4.523863	4.20073	1.292532	4.523863
22	7.432061	0.323133	3.554464	20.35739	4.20073	23.26558	0.969399	15.18726	4.846996	1.938799	9.37086
21	4.846996	0.969399	1.615665	13.89472	1.938799	10.01713	1.292532	3.231331	6.462662	2.585065	6.139529
20	5.493263	1.938799	2.585065	14.54099	1.615665	17.12605	3.231331	7.432061	3.554464	2.908198	6.785795
19	6.462662	0.969399	2.585065	15.18726	2.585065	17.44919	2.261932	5.493263	7.432061	2.585065	6.462662
18	3.877597	0.969399	1.615665	4.523863	0.969399	13.24846	1.615665	2.261932	3.554464	1.938799	3.877597
17	0.646266	0	0.323133	1.615665	0.646266	3.877597	0.323133	0.969399	0.646266	0.646266	1.292532
16	21.00365	4.846996	9.693993	61.07216	9.37086	89.831	7.432061	26.17378	24.23498	9.693993	25.52751
15	2.908198	0.323133	1.615665	7.108928	1.292532	8.724594	2.261932	2.585065	1.292532	0.646266	2.908198
14	2.908198	0.646266	0.646266	7.755194	0.969399	12.92532	0	3.877597	1.938799	1.615665	1.938799
13	10.01713	0.969399	2.908198	26.49691	4.846996	35.22151	4.846996	13.24846	9.047727	3.877597	12.92532
12	3.231331	0	1.938799	6.785795	0.969399	13.24846	0.969399	5.816396	3.877597	2.585065	3.877597
11	10.34026	1.938799	3.231331	33.92898	2.585065	33.28271	3.554464	11.30966	12.92532	3.877597	6.462662
10	7.432061	1.292532	3.877597	15.18726	5.17013	19.06485	3.231331	9.047727	6.139529	2.585065	6.139529
9	13.24846	1.615665	1.615665	24.55812	3.554464	31.34391	5.816396	13.57159	6.785795	4.20073	12.27906
8	4.846996	1.292532	3.877597	15.18726	3.877597	22.29618	1.938799	5.493263	4.523863	3.231331	6.785795
7	8.724594	0.969399	2.908198	10.98653	1.938799	13.24846	1.938799	6.785795	3.554464	2.908198	7.108928
6	8.724594	3.231331	4.846996	24.23498	2.908198	23.58872	2.261932	8.078327	9.693993	2.908198	8.724594
5	3.877597	0.323133	1.615665	5.493263	0.646266	11.30966	1.938799	3.554464	2.585065	1.292532	4.846996
4	24.88125	4.523863	6.462662	22.94245	12.27906	84.33774	9.37086	28.75885	18.09545	4.20073	26.82005
3	2.908198	0.323133	0	2.908198	0.646266	5.816396	0.969399	2.585065	1.615665	0.969399	1.938799
2	8.724594	0	3.554464	15.83352	2.585065	18.41859	3.877597	6.139529	2.261932	3.231331	5.17013
1	7.108928	1.938799	5.17013	35.22151	5.493263	49.7625	4.20073	16.15665	13.57159	7.108928	22.94245
	1	2	3	4	5	6	7	8	9	10	11

35	0.323133	0.323133	0.323133	0.323133	1.292532	0	0.323133	0.969399	0.646266	0.323133	1.292532	0.323133
34	4.846996	8.078327	4.20073	3.231331	16.80292	3.877597	3.554464	3.877597	5.17013	2.261932	8.724594	6.139529
33	4.846996	7.755194	3.554464	3.877597	14.86412	2.585065	1.938799	0.969399	2.908198	1.615665	6.139529	4.20073
32	8.078327	17.77232	4.523863	5.816396	23.26558	6.785795	2.908198	1.938799	7.755194	4.846996	21.32678	8.078327
31	1.615665	5.17013	2.261932	0.646266	7.108928	2.261932	1.292532	1.615665	1.615665	1.615665	3.877597	3.554464
30	0.969399	5.17013	2.585065	2.908198	5.816396	1.292532	1.615665	1.292532	3.231331	2.261932	2.908198	4.20073
29	15.83352	37.48344	9.047727	18.41859	48.7931	19.06485	10.66339	12.60219	11.95592	14.86412	28.43571	21.64992
28	3.877597	11.30966	2.261932	5.816396	15.83352	1.938799	1.938799	2.261932	5.493263	2.585065	6.139529	5.493263
27	4.846996	14.21786	2.908198	5.17013	23.26558	4.523863	6.462662	2.908198	6.462662	3.877597	12.27906	8.078327
26	1.615665	4.846996	2.261932	3.877597	8.724594	2.585065	2.908198	1.292532	2.585065	2.908198	6.139529	3.554464
25	3.231331	15.51039	7.432061	9.047727	19.06485	4.20073	4.20073	2.261932	6.462662	4.523863	9.693993	8.078327
24	8.078327	20.03425	6.139529	7.108928	32.95958	5.493263	5.493263	3.231331	10.66339	4.20073	13.57159	10.34026
23	3.877597	8.078327	1.615665	4.20073	9.693993	1.938799	3.231331	1.615665	0.969399	0.646266	4.846996	2.908198
22	3.877597	8.401461	1.615665	3.554464	14.21786	1.938799	2.585065	1.938799	4.846996	2.585065	4.20073	3.231331
21	1.938799	3.231331	1.938799	0.646266	8.724594	1.615665	1.292532	1.615665	2.585065	1.615665	3.231331	1.615665
20	5.816396	9.693993	3.554464	4.20073	6.139529	2.585065	1.938799	1.292532	2.261932	3.554464	3.554464	3.231331
19	3.231331	6.139529	2.585065	4.523863	7.755194	3.554464	1.938799	2.261932	2.908198	3.877597	7.432061	6.139529
18	0.969399	4.846996	2.261932	5.816396	5.816396	4.523863	0.969399	0.969399	2.908198	1.938799	2.908198	2.585065
17	0.323133	0.969399	0.969399	0.646266	1.292532	0.323133	0.323133	0	0.323133	0.323133	0	0.646266
16	9.37086	39.0991	12.92532	14.21786	28.43571	12.92532	8.078327	5.816396	13.89472	9.047727	25.20438	14.21786
15	1.615665	2.908198	0.969399	2.261932	4.523863	0.969399	0.323133	1.615665	0.646266	0.969399	2.261932	3.554464
14	3.877597	3.877597	0.969399	4.20073	5.816396	1.292532	0.646266	1.292532	2.261932	1.615665	5.816396	3.554464
13	6.462662	13.24846	6.785795	6.139529	22.61932	5.493263	4.846996	5.17013	7.432061	4.20073	13.24846	7.432061
12	0.969399	1.938799	0.969399	2.585065	5.816396	1.938799	2.261932	0.646266	0.969399	0.323133	3.877597	3.231331
11	4.523863	17.44919	7.755194	5.493263	21.00365	4.20073	2.585065	3.554464	6.462662	5.816396	12.60219	7.432061
10	2.585065	8.401461	0.969399	3.877597	9.693993	2.908198	2.908198	2.908198	2.585065	1.292532	8.401461	3.877597
9	8.401461	14.21786	4.523863	4.523863	15.18726	7.108928	1.938799	2.585065	4.846996	6.462662	9.047727	9.047727
8	2.908198	9.37086	3.231331	6.139529	9.37086	1.938799	1.938799	0.969399	3.877597	3.231331	6.462662	3.554464
7	3.231331	7.432061	2.585065	3.554464	9.37086	2.261932	2.908198	0.969399	3.554464	2.261932	4.523863	3.877597
6	2.908198	9.693993	1.615665	7.108928	14.54099	1.615665	2.908198	0.969399	4.20073	0.646266	9.37086	6.462662
5	0.969399	3.231331	1.615665	1.938799	4.846996	1.615665	1.615665	0.969399	1.938799	0.969399	0	3.554464
4	12.27906	27.46631	12.92532	13.89472	44.59237	8.078327	7.432061	8.401461	11.95592	10.34026	20.35739	14.54099
3	1.292532	1.938799	1.615665	0.646266	3.877597	0.323133	0.646266	0.969399	1.292532	0.646266	0.646266	0.969399
2	1.292532	5.17013	1.938799	3.554464	12.27906	2.261932	1.292532	1.938799	2.585065	2.261932	4.523863	3.877597
1	11.30966	12.27906	5.17013	9.37086	25.20438	7.108928	2.908198	4.523863	6.139529	9.37086	17.77232	8.724594
	12	13	14	15	16	17	18	19	20	21	22	23

35	0.646266	0.323133	0.323133	0	1.292532	0.323133	1.615665	0.969399	0.646266	0.323133	0	0
34	15.18726	9.047727	3.877597	6.462662	18.41859	6.785795	13.57159	8.401461	21.00365	2.585065	1.292532	4.523863
33	12.60219	9.37086	4.523863	3.877597	13.24846	9.693993	13.24846	6.462662	20.35739	2.261932	1.615665	3.554464
32	25.85065	13.89472	14.54099	8.401461	27.78945	18.74172	32.31331	10.66339	26.17378	6.139529	1.938799	8.724594
31	4.20073	3.554464	1.615665	1.938799	7.755194	4.523863	8.401461	0.323133	10.98653	4.20073	0.969399	1.615665
30	9.693993	3.554464	2.585065	2.585065	9.37086	4.523863	6.139529	1.938799	11.63279	2.908198	0.646266	1.615665
29	61.07216	25.52751	18.41859	24.23498	57.51769	10.66339	56.87143	17.44919	66.56542	10.98653	3.554464	12.27906
28	16.80292	8.401461	4.20073	10.01713	19.06485	10.01713	20.68052	5.493263	20.35739	3.231331	1.938799	3.877597
27	24.23498	11.95592	5.17013	7.755194	18.74172	13.24846	24.55812	7.108928	37.16031	3.554464	2.908198	4.20073
26	11.63279	8.401461	1.938799	4.846996	9.693993	4.846996	12.60219	3.554464	12.60219	2.261932	0.323133	4.20073
25	23.26558	3.877597	9.047727	10.66339	24.55812	14.54099	28.43571	5.17013	27.46631	2.585065	1.938799	4.846996
24	24.88125	17.12605	9.37086	10.66339	30.37451	16.80292	43.62297	9.37086	39.74537	6.462662	2.261932	5.17013
23	9.37086	3.877597	5.17013	5.816396	9.047727	7.108928	12.60219	3.877597	15.83352	1.615665	0.646266	2.908198
22	10.66339	4.20073	2.908198	3.877597	10.98653	5.816396	15.83352	2.908198	20.35739	3.554464	0	2.261932
21	11.63279	3.554464	2.908198	2.261932	11.30966	6.462662	8.724594	2.261932	12.27906	0.646266	0.323133	2.261932
20	9.047727	6.139529	4.846996	3.554464	10.01713	2.261932	13.24846	2.908198	13.89472	2.585065	1.292532	2.585065
19	12.27906	8.401461	2.908198	2.261932	11.30966	4.846996	12.60219	2.585065	13.89472	2.585065	1.292532	3.231331
18	4.20073	6.139529	2.585065	3.877597	5.816396	3.554464	6.785795	3.231331	7.755194	0.323133	0.323133	0.646266
17	3.231331	1.292532	0.323133	1.292532	1.615665	0.646266	2.261932	0.323133	3.231331	0.323133	0	0.646266
16	50.08563	23.91185	18.09545	19.06485	50.7319	29.40511	57.19456	16.15665	64.30349	9.693993	5.493263	9.693993
15	3.554464	1.938799	3.231331	3.554464	4.523863	1.938799	5.816396	1.938799	7.755194	1.292532	0.969399	1.292532
14	8.724594	2.585065	2.908198	2.261932	5.493263	4.523863	8.078327	1.615665	4.523863	1.615665	0.646266	0.646266
13	23.91185	9.37086	9.37086	13.24846	23.58872	13.57159	30.05138	6.785795	36.19091	4.846996	2.261932	4.523863
12	4.523863	3.231331	2.261932	2.908198	4.846996	3.554464	6.785795	1.615665	4.523863	0.646266	0.323133	2.261932
11	30.05138	8.401461	8.078327	8.401461	25.85065	10.66339	29.08198	6.785795	32.31331	4.846996	1.615665	2.585065
10	11.95592	11.95592	5.17013	5.816396	15.18726	6.785795	15.51039	3.554464	14.54099	3.231331	1.292532	4.523863
9	21.32678	8.724594	6.785795	10.34026	18.41859	15.51039	19.38799	5.17013	29.40511	1.615665	2.261932	6.139529
8	22.94245	6.785795	3.877597	2.585065	11.30966	5.17013	15.18726	3.231331	13.24846	2.585065	1.938799	1.938799
7	10.66339	4.20073	5.493263	5.17013	6.462662	7.108928	13.57159	3.231331	17.77232	1.615665	0.323133	4.846996
6	22.29618	9.693993	5.816396	8.401461	17.44919	12.92532	30.37451	4.846996	22.94245	5.17013	0.323133	5.816396
5	5.816396	1.292532	3.877597	3.877597	6.785795	4.523863	6.785795	1.615665	8.078327	0.969399	0.323133	1.615665
4	52.02443	19.71112	21.32678	17.12605	44.9155	25.85065	53.64009	8.724594	60.10276	6.785795	2.261932	10.34026
3	2.261932	2.261932	0.969399	0.969399	1.938799	0.969399	5.493263	0	4.20073	0.969399	0	0.323133
2	8.724594	4.20073	5.816396	6.462662	13.89472	4.846996	13.24846	3.554464	10.98653	2.261932	0.646266	1.292532
1	31.02078	14.54099	10.66339	10.98653	26.17378	13.89472	32.63644	10.34026	37.48344	4.846996	2.585065	7.108928
	24	25	26	27	28	29	30	31	32	33	34	35

Table S9. Frequency of the different dual-sgRNA cassettes of HK-L2 library in *Vibrio* FA2 containing dCas9 before inducing.

35	0.770713	0	0	1.156069	0	0.770713	0	0.385356	0	0	0	0	1.156069	0	0	0	0	0	0	0	0.385356	0	0	0.385356	0	0	0.385356	0.770713	0	0.770713	0	3.468208	0	0	0
34	10.01927	0.385356	1.156069	32.36994	1.156069	35.06744	0	2.312139	2.697495	3.853565	6.55106	0.770713	16.95568	1.156069	0.385356	19.65318	0	0	1.926782	0.770713	0	13.10212	1.926782	22.73603	2.312139	3.082852	1.541426	7.707129	3.853565	28.51638	0.770713	37.76493	1.156069	3.082852	1.156069
33	1.926782	0.385356	0	16.57033	0	25.81888	0	0.770713	1.156069	0.770713	3.468208	0	8.477842	1.156069	0	6.936416	0	0	1.156069	0	0.385356	1.541426	1.926782	8.477842	0.385356	1.541426	0.770713	3.853565	2.697495	12.33141	0.385356	20.42389	0	0	0
32	17.7264	0.770713	1.156069	68.20809	1.926782	124.4701	1.156069	6.165703	10.40462	0.385356	11.94605	0.770713	81.69557	4.624277	1.156069	43.93064	0.770713	1.156069	0.770713	1.926782	0	18.49711	5.009634	55.10597	3.853565	12.33141	6.165703	23.12139	8.092486	81.31021	1.926782	75.52987	3.082852	5.009634	1.541426
31	0.385356	0	0	1.926782	0	5.780347	0	1.541426	0	0	0.385356	0	1.926782	0	0	1.156069	0.385356	0	0	0	0	0	0	2.312139	0	0.770713	0	0.385356	0	6.165703	0	3.853565	0	0.385356	0
30	3.082852	0	0	10.40462	0	25.81888	0	1.156069	1.541426	0.385356	2.697495	0	10.78998	0.385356	0.385356	8.092486	0	0.385356	0	0.770713	0.385356	2.697495	2.697495	14.25819	0.385356	2.312139	2.312139	3.468208	4.238921	10.78998	0	27.74566	0	0	0
29	31.98459	1.926782	2.312139	111.368	2.697495	189.9807	0.770713	15.41426	13.10212	4.238921	21.57996	3.468208	97.49518	7.707129	2.697495	57.41811	0.770713	0.770713	0.385356	3.082852	1.541426	32.7553	7.321773	67.82274	8.092486	14.64355	7.707129	35.45279	10.78998	108.6705	3.082852	196.5318	5.39499	8.477842	1.156069
28	6.165703	0.385356	0	17.34104	1.156069	35.83815	0.385356	2.312139	4.238921	0.385356	3.853565	0.385356	18.11175	0.770713	0	18.11175	0	0.385356	0	1.156069	0	4.624277	1.541426	14.25819	1.541426	1.541426	2.312139	2.697495	3.082852	26.97495	0.385356	38.921	0.770713	1.926782	0
27	5.39499	0.385356	1.926782	21.96532	0	36.99422	0	1.541426	1.541426	0	5.39499	0	26.5896	0.385356	1.156069	18.88247	0.385356	0.385356	0	0.770713	0.385356	5.39499	2.312139	25.81888	1.541426	2.697495	0.385356	10.78998	5.009634	35.83815	0.385356	41.23314	1.156069	1.541426	1.926782
26	7.707129	0.385356	0	29.67245	0.770713	41.6185	0	1.541426	1.926782	0.770713	6.165703	1.156069	25.04817	0.770713	0.770713	6.55106	0	1.926782	0	0.770713	0	11.17534	2.312139	15.79961	3.082852	2.312139	3.082852	5.009634	2.312139	30.0578	0	38.15029	0.770713	0.385356	0
25	4.238921	0	0	16.57033	0	29.28709	0	1.926782	1.926782	0	3.853565	0.385356	17.7264	0.770713	0	10.01927	0	1.541426	0.385356	0.385356	0	2.312139	1.156069	11.56069	0.385356	1.926782	0.770713	8.477842	3.468208	17.34104	0.770713	28.13102	1.926782	1.156069	0.385356
24	15.0289	1.156069	1.156069	60.11561	1.926782	130.6358	1.541426	8.092486	5.009634	3.082852	8.092486	0.770713	80.92486	1.541426	1.541426	37.37958	0.770713	1.541426	0.385356	1.541426	0.770713	18.88247	3.468208	39.69171	4.238921	5.39499	5.009634	22.73603	16.57033	82.46628	1.541426	138.7283	2.312139	3.082852	1.156069
23	4.238921	0.770713	0.385356	11.94605	0	21.96532	0	2.697495	1.541426	0.385356	1.156069	0	15.41426	0.770713	0.770713	7.321773	0.385356	0.770713	0	0.770713	0	1.926782	0.770713	17.34104	0.770713	2.312139	1.541426	5.780347	1.926782	13.48748	0.770713	30.0578	0	0	0
22	5.009634	0.385356	0.770713	24.66281	0.385356	39.69171	0.385356	2.697495	1.156069	1.926782	4.624277	0.385356	31.59923	0	1.156069	11.94605	0	0.385356	0.385356	1.156069	0	3.082852	1.926782	20.03854	2.312139	1.926782	1.156069	7.707129	5.009634	34.68208	0.770713	47.7842	0	1.156069	0
21	0.770713	0	0	0.770713	0	2.312139	0	0	0	0.385356	0.770713	0.770713	1.926782	0.770713	0	1.541426	0	0	0	0	0	0.385356	0	0.770713	0.385356	0.385356	0	0.770713	0.770713	1.541426	0	3.853565	0	0.385356	0.385356
20	2.697495	0	0	7.321773	0.770713	8.863198	0	1.541426	0.385356	0	1.156069	0.385356	6.55106	0.385356	1.156069	5.009634	0	0	0.385356	0	0	1.926782	1.156069	5.780347	0	1.926782	0	1.156069	1.541426	9.248555	0	8.477842	0	0	0
19	1.541426	0	1.156069	5.39499	0.385356	13.10212	0.385356	0.385356	2.312139	0.385356	1.926782	1.541426	6.936416	0.770713	0.385356	1.926782	0	0	0	1.156069	0	1.541426	0.385356	6.55106	0.385356	1.541426	0	1.541426	1.541426	6.936416	0	10.40462	0	0.385356	0
18	1.156069	0	0	1.541426	0	4.624277	0	0	0	0	0.770713	0	1.926782	0	0	1.156069	0	0	0.385356	0	0	0.770713	0.770713	2.312139	0	1.156069	0	1.926782	0.385356	7.707129	0	6.165703	0	0	0
17	0.385356	0	0	0	0	0.385356	0	0	0	0	0	0	0.385356	0	0	0	0	0	0	0	0	0.385356	0	0.385356	1.156069	0	0	0	0	0	0	0	0	0	0
16	28.51638	0.770713	1.541426	111.368	2.312139	233.1407	2.312139	12.71676	11.17534	4.238921	26.20424	4.238921	140.6551	5.780347	2.697495	33.14066	1.541426	3.468208	0	3.853565	1.926782	32.36994	10.78998	88.24663	7.707129	13.48748	11.17534	56.6474	23.50674	129.0944	3.853565	210.4046	6.936416	4.238921	1.926782
15	0.770713	0	0	0.385356	0	1.541426	0	0	0.770713	0	0	0	1.541426	0	0.385356	0.770713	0	0	0	0	0	0.385356	0	0.770713	0	0.770713	0	0.385356	0	1.156069	0	1.156069	0	0	0
14	0	0	0	2.697495	0	6.936416	0	0.385356	0	0	0	0.385356	4.624277	0	0.770713	2.312139	0	0.385356	0	0	0.385356	0.770713	0.385356	5.009634	0.770713	0.385356	0	0.770713	0.385356	5.780347	0	7.321773	0	0	0
13	28.90173	1.926782	1.926782	103.6609	3.082852	156.4547	1.156069	12.71676	10.01927	2.697495	19.65318	4.624277	59.34489	8.863198	0.770713	67.82274	0.770713	1.926782	1.926782	6.936416	1.926782	24.66281	11.17534	67.43738	9.633911	15.0289	10.01927	42.38921	24.27746	110.5973	2.312139	182.659	5.39499	11.56069	0.385356
12	0.385356	0	0	2.312139	0	3.853565	0	0.770713	0.385356	0	2.312139	0	2.697495	0	0.385356	1.156069	0	2.697495	0	0.385356	0	0.770713	0	2.312139	0.385356	1.156069	0	0.770713	0.385356	1.541426	0	5.009634	0	0	0
11	5.780347	0.385356	1.156069	26.20424	1.541426	58.57418	0.385356	4.238921	4.238921	1.156069	0.385356	0.770713	40.07707	0.770713	0	21.19461	0.385356	0.385356	1.541426	0.385356	0	7.707129	4.624277	25.43353	3.468208	3.468208	3.468208	6.165703	3.082852	35.45279	1.541426	62.8131	2.312139	2.312139	0
10	5.780347	0	0	13.48748	0.385356	21.19461	0	1.156069	1.156069	1.541426	1.541426	0	12.71676	0.385356	0.770713	7.707129	0.770713	0	0.385356	0	0	2.697495	0.770713	10.01927	16.18497	2.312139	1.541426	3.468208	1.926782	13.48748	0	22.73603	0.385356	0.770713	0.385356
9	6.936416	0	0.770713	14.64355	1.926782	41.6185	0	5.780347	1.541426	1.156069	3.853565	0	28.51638	1.926782	0	11.56069	0.385356	0	0.385356	0.385356	1.156069	8.477842	1.926782	18.88247	1.156069	4.624277	1.541426	10.78998	3.082852	30.44316	0.385356	45.08671	0.770713	2.312139	0.385356
8	4.238921	0.385356	3.082852	12.33141	0	15.79961	0.385356	0.770713	0.770713	1.541426	1.156069	0	19.65318	0.385356	0	7.707129	0	0.385356	0	0.385356	0.385356	3.468208	0.770713	26.97495	0.770713	0.770713	1.156069	2.312139	1.926782	9.248555	0.385356	14.64355	0.385356	0.385356	0
7	0.770713	0	0	2.312139	0.770713	8.477842	0	3.082852	0	0	6.936416	0	6.165703	2.312139	0	1.541426	0	0.385356	0	0.385356	0.385356	1.926782	0	1.541426	1.541426	0.770713	0.385356	3.082852	0	4.238921	0	11.56069	0	0	0
6	20.80925	1.156069	4.238921	40.07707	1.926782	51.25241	0.385356	3.853565	4.624277	1.926782	9.633911	0.385356	56.26204	0.770713	0.770713	18.88247	1.156069	0.770713	0.385356	1.541426	1.541426	11.56069	3.082852	41.6185	2.312139	5.39499	2.312139	15.0289	6.55106	75.91522	1.156069	92.10019	3.468208	1.926782	0.770713
5	2.697495	0	0	3.082852	0.385356	5.39499	0	0	0	0.385356	0.385356	0	7.707129	0	0	0.770713	0	0	0.385356	0	0	0	0	3.082852	0.385356	0.770713	0	0.770713	0.770713	3.468208	0	6.165703	0.385356	0	0
4	38.53565	1.156069	3.082852	44.31599	2.697495	214.6435	1.926782	16.95568	10.78998	2.697495	22.73603	2.312139	115.2216	5.780347	1.926782	63.58382	1.541426	1.926782	0.770713	2.697495	0.770713	28.90173	8.863198	91.71484	9.633911	15.41426	8.092486	44.70135	13.87283	124.4701	2.697495	210.0193	4.238921	2.697495	0.385356
3	0.385356	0	0	0.385356	0	1.156069	0	0	0.385356	0	0.770713	0	2.312139	1.156069	0.385356	1.156069	0	0	0	0	0	0.770713	0.770713	0.770713	0	0	0.385356	0	0	1.541426	0	2.312139	0	0	0
2	1.156069	0.770713	0.385356	5.39499	0.770713	11.94605	0.385356	0.385356	1.926782	0.385356	0.770713	0.385356	9.633911	0	0	3.468208	0	0	0	0.385356	0	1.156069	0.385356	3.853565	0	0.385356	0.385356	2.312139	2.312139	10.01927	0	15.0289	0.770713	1.156069	0.385356
1	3.853565	1.156069	1.541426	33.14066	1.541426	81.31021	0.385356	7.321773	7.321773	3.468208	11.94605	1.156069	52.02312	0.770713	0	20.42389	0.770713	1.541426	1.156069	3.468208	0.385356	21.19461	4.238921	42.38921	2.697495	7.321773	2.312139	20.03854	9.248555	63.58382	1.156069	89.78805	1.926782	2.312139	0
	1	2	3	4	5	6	7	8	9	10	11	12	13	14	15	16	17	18	19	20	21	22	23	24	25	26	27	28	29	30	31	32	33	34	35

Table S10. Frequency of the different dual-sgRNA cassettes of HK-L2 library in *Vibrio* FA2 containing dCas9 after inducing.

35	0.09399	0	0	0.09399	0	0.751922	0	0.09399	0	0	0.09399	0	0.375961	0	0	0.09399	0	0	0	0	0	0	0	0.09399	0	0	0	0	0	0.09399	0	0.375961	0	0	0
34	8.365133	0.187981	1.315864	14.00455	0.845912	28.19708	0	1.879805	3.47764	2.819708	7.895182	0.375961	30.07688	1.503844	0	22.55766	0.09399	0.187981	2.537737	0.187981	0	10.33893	3.007688	14.66248	1.879805	1.785815	1.315864	3.759611	3.947591	8.271143	0.751922	49.34489	1.503844	3.101679	0.657932
33	3.853601	0.281971	0.09399	11.27883	0.281971	23.30959	0	1.503844	0.563942	0.375961	2.067786	0.375961	23.77954	0.09399	0	14.19253	0.09399	0.09399	0.281971	0.281971	0.09399	6.203357	0.845912	7.989172	0.657932	0.845912	1.409854	3.007688	2.349757	5.357445	0.375961	33.83649	0.187981	0.281971	0.09399
32	19.36199	0.563942	0.939903	43.70547	1.033893	132.4323	0.187981	6.767299	6.015377	1.503844	10.43292	0.939903	173.412	3.383649	0.657932	49.62686	0.281971	1.503844	0.845912	1.221873	0.375961	27.72713	3.195669	52.35258	3.289659	8.835085	3.57163	11.65479	14.28652	33.46053	0.375961	123.5032	1.315864	5.451435	0.187981
31	0.563942	0	0	0.375961	0	2.819708	0.09399	0.281971	0.09399	0	0	0	2.161776	0.09399	0.281971	1.221873	0.281971	0.09399	0	0	0	0.751922	0.187981	0.845912	0.281971	0.09399	0.375961	0.281971	0.657932	1.033893	0	4.041581	0	0.281971	0.09399
30	1.503844	0.09399	0	3.947591	0	7.519221	0	0.469951	0.939903	0.09399	0.751922	0	7.613211	0.281971	0	2.819708	0	0	0	0.281971	0.09399	2.349757	0.187981	3.47764	0.09399	0.469951	0.09399	1.315864	0.939903	2.631727	0.09399	15.97834	0.469951	0.09399	0
29	26.31727	1.503844	3.947591	60.52973	3.57163	158.4676	0.657932	10.43292	10.6209	2.067786	15.97834	1.409854	167.5846	8.835085	2.067786	59.02588	0.469951	1.221873	1.033893	1.785815	1.691825	49.06292	7.613211	53.57445	4.323552	15.22642	6.297348	19.36199	10.05696	33.17856	1.033893	229.5242	5.639416	6.391338	1.315864
28	1.409854	0	0	4.417542	0.281971	10.90287	0.09399	0.939903	0.375961	0	1.315864	0	20.20791	0.281971	0.09399	6.955279	0	0.281971	0	0.09399	0	2.725718	0.657932	8.365133	0.281971	0.657932	0.375961	0.845912	1.315864	2.913698	0.187981	21.80574	0.187981	0.469951	0
27	3.947591	0.281971	0.187981	12.5007	0.09399	29.8889	0	1.503844	1.503844	0.187981	1.879805	0.375961	32.05068	0.563942	0	10.80888	0	0.09399	0.09399	0.09399	0.469951	8.929075	0.939903	14.28652	0.187981	2.537737	1.127883	2.443747	2.725718	17.85815	0.187981	40.5098	0.563942	1.221873	0.281971
26	2.725718	0.281971	0.939903	10.6209	0.563942	49.53287	0.187981	2.819708	2.443747	0.657932	4.605523	0.09399	43.32951	1.033893	0.187981	14.19253	0	1.315864	0.469951	0.563942	0.187981	13.15864	0.657932	23.1216	2.913698	2.631727	1.879805	2.819708	7.425231	8.835085	0.375961	56.48815	0.469951	1.315864	0.187981
25	4.041581	0.09399	0.469951	10.05696	0.187981	24.24949	0.187981	1.409854	1.033893	0.751922	3.289659	0.187981	43.51749	0.751922	0.09399	9.305036	0	0.187981	0.09399	0.09399	0.281971	4.511533	1.315864	14.4745	0.281971	0.939903	0.939903	1.597834	3.195669	5.921387	0	42.67158	1.033893	0.845912	0
24	12.12474	0.469951	0.845912	31.11078	0.751922	113.7282	0.563942	4.135572	5.357445	0.469951	8.365133	1.033893	178.8635	2.349757	0.09399	44.17542	0.09399	0.375961	0.469951	0.657932	0.281971	16.82426	2.537737	48.68696	1.973796	5.921387	2.537737	8.929075	7.04927	25.94131	0.939903	166.8327	2.161776	2.631727	0.375961
23	2.819708	0.187981	0.187981	5.921387	0.09399	16.4483	0.187981	1.409854	1.127883	0	1.973796	0.09399	19.54997	0.469951	0	8.271143	0	0.09399	0.09399	0.09399	0	3.759611	0.281971	9.962968	1.033893	0.657932	0.939903	1.315864	1.127883	2.819708	0.09399	23.87353	0.281971	0.563942	0.187981
22	5.639416	0.187981	0.187981	15.03844	0.657932	46.24321	0.09399	3.759611	3.57163	0.657932	5.921387	0.469951	71.52659	1.033893	0.187981	22.08771	0.187981	0.375961	0.187981	0.751922	0.469951	7.519221	2.067786	18.79805	1.597834	2.819708	1.691825	5.075474	6.767299	12.31272	0.375961	75.09822	0.563942	1.221873	0.751922
21	0.281971	0	0	0.845912	0	1.785815	0	0	0.281971	0.09399	0.187981	0.09399	1.691825	0.09399	0.09399	0.563942	0	0	0	0	0	0.281971	0	0.939903	0	0.281971	0.09399	0.375961	0.187981	0.375961	0	2.067786	0.09399	0.09399	0
20	0.751922	0	0	2.819708	0	4.417542	0.09399	0.281971	0.657932	0	0.469951	0.09399	7.33124	0.09399	0	3.759611	0	0.09399	0	0	0	1.785815	0.281971	4.229562	0	0.281971	0.375961	0.751922	0.939903	0.845912	0	12.21873	0	0.09399	0
19	2.631727	0.187981	0.281971	6.673309	0.09399	15.79036	0.09399	1.597834	1.033893	0.09399	2.161776	0.469951	38.63	0.375961	0	4.417542	0	0.375961	0.281971	0.281971	0.09399	4.699513	0.845912	11.5608	0.469951	1.785815	0.657932	1.785815	4.229562	3.853601	0.09399	34.02448	0.187981	0.563942	0.375961
18	0.469951	0	0	0.751922	0	2.537737	0.09399	0.09399	0.563942	0	0.563942	0.187981	3.853601	0.751922	0	1.127883	0	0	0	0	0	1.127883	0.09399	0.469951	0	0.187981	0	1.409854	0.469951	0.845912	0.187981	6.767299	0	0	0.187981
17	0.09399	0	0	0.187981	0	0.09399	0	0	0.09399	0	0	0.09399	0.281971	0	0	0.187981	0	0	0	0	0	0	0	0.281971	0.375961	0	0	0.375961	0.09399	0	0	0.469951	0	0	0
16	26.0353	1.597834	1.785815	66.45112	2.349757	230.6521	0.375961	15.03844	19.64396	4.605523	20.11392	1.597834	293.7196	5.545425	0.375961	68.61289	0.563942	2.443747	1.503844	3.57163	1.221873	49.06292	11.27883	111.7544	7.14326	15.79036	6.673309	30.35885	27.35117	42.20163	1.315864	305.6563	5.357445	7.801192	0.563942
15	0.09399	0	0	0.09399	0.09399	1.127883	0	0	0.375961	0	0.09399	0.09399	0.563942	0	0.187981	0.375961	0	0	0	0	0	0.09399	0.375961	0.375961	0.187981	0	0	0.187981	0.09399	0.09399	0	0.751922	0	0.09399	0
14	1.973796	0	0.187981	1.503844	0.187981	6.297348	0.187981	0	0.563942	0	1.221873	0.09399	9.962968	0.187981	0.281971	2.067786	0	0.09399	0	0.09399	0.281971	1.691825	0.09399	4.229562	0.375961	0.657932	0.281971	1.409854	0.751922	2.537737	0.09399	8.929075	0.09399	0.751922	0.281971
13	53.01051	2.161776	4.135572	89.29075	4.041581	263.3607	1.033893	19.73796	28.949	3.195669	48.87494	4.605523	212.324	21.42978	1.221873	123.7852	1.033893	3.853601	3.101679	5.169464	2.913698	68.61289	15.03844	115.514	10.6209	30.9228	17.85815	42.67158	46.3372	63.81939	2.631727	517.6984	6.955279	12.5947	2.443747
12	0.939903	0	0	1.409854	0	1.973796	0.187981	0.09399	0.187981	0	1.315864	0.09399	5.545425	0.187981	0.09399	0.657932	0	0.281971	0	0	0	0.939903	0	1.879805	0	0.09399	0	0.09399	0.281971	0.657932	0	3.759611	0	0	0.09399
11	5.451435	0.375961	1.033893	13.15864	0.375961	28.19708	0.281971	2.443747	2.819708	0.751922	2.067786	0.469951	55.07829	0.657932	0.09399	16.07233	0	0.187981	0.563942	0.09399	0.187981	9.680997	1.221873	20.20791	1.127883	1.127883	1.033893	2.067786	3.101679	9.680997	0.09399	77.35399	0.563942	1.691825	0
10	1.127883	0.09399	0	2.913698	0.281971	12.78268	0	0.751922	0.751922	0.187981	0.751922	0.09399	14.38051	0.375961	0.09399	2.349757	0	0	0.09399	0.563942	0.187981	2.725718	0.281971	4.981484	8.083163	0.751922	0.469951	1.127883	0.657932	1.879805	0	14.85046	0.09399	0.563942	0.281971
9	5.921387	0.281971	0.375961	10.6209	0.09399	35.7163	0.187981	2.443747	1.315864	0.469951	3.195669	0.375961	63.06747	1.503844	0.09399	13.34662	0.09399	0.281971	0.09399	0.469951	0.281971	6.203357	0.939903	21.99372	0.845912	1.973796	1.879805	2.537737	4.793503	9.587007	0.375961	79.32778	0.469951	2.161776	0
8	2.255766	0	0.657932	4.229562	0.375961	12.78268	0.09399	0.657932	0.939903	0.187981	0.563942	0.09399	36.75019	0.845912	0	6.767299	0	0.281971	0.187981	0.09399	0	2.725718	1.597834	20.20791	0.187981	0.657932	0.281971	0.845912	1.691825	4.229562	0.09399	21.80574	0.469951	0.751922	0.281971
7	0.751922	0	0	1.409854	1.503844	3.195669	0	1.221873	0.281971	0.09399	4.793503	0.563942	3.66562	0.563942	0.187981	1.221873	0.09399	0.187981	0.187981	0.09399	0	0.563942	0.187981	1.597834	0.563942	0.375961	0	0.563942	1.221873	0.845912	0	5.827396	0.657932	0.09399	0
6	11.09085	0.563942	1.597834	24.24949	0.845912	40.5098	0	4.229562	4.323552	0.845912	6.297348	0.281971	132.7143	1.127883	0.09399	28.19708	0.09399	0.563942	0.375961	0.375961	0.469951	21.05382	2.255766	43.51749	1.879805	3.289659	0.939903	7.895182	6.579318	24.71944	0.375961	94.5542	0.845912	0.939903	0.09399
5	0.845912	0	0.09399	0.939903	0	3.947591	0	0.751922	0.751922	0	1.409854	0.09399	6.955279	0.09399	0	2.819708	0	0.09399	0.09399	0	0.09399	1.221873	0.657932	2.255766	0.939903	0.845912	0.375961	0.939903	0.563942	0.845912	0.187981	7.519221	0	0.187981	0.09399
4	16.35431	0.375961	0.939903	12.03075	1.409854	87.22296	0.375961	7.519221	5.639416	1.315864	7.707202	1.033893	114.7621	2.067786	0.751922	42.4836	0.469951	0.845912	0.469951	0.375961	0.281971	17.29421	2.819708	43.14153	1.503844	5.357445	3.007688	9.868978	9.962968	23.30959	0.563942	150.4784	1.879805	3.947591	0.281971
3	0.09399	0	0	0.375961	0	0.657932	0	0.09399	0	0	0.09399	0.09399	1.691825	0.845912	0	0.751922	0	0	0	0	0	0.657932	0	0.657932	0.09399	0	0	0.281971	0.187981	1.033893	0.09399	2.631727	0.187981	0.09399	0
2	3.101679	0.09399	0.281971	3.66562	0.281971	16.54229	0.09399	0.751922	0.281971	0.09399	1.033893	0.09399	10.15095	0.09399	0	4.229562	0.187981	0.187981	0.09399	0.09399	0.281971	2.067786	0.469951	4.135572	0.09399	0.845912	0.187981	0.845912	1.127883	1.597834	0.187981	19.36199	0.563942	0.375961	0
1	8.553114	0.187981	0.469951	22.08771	0.751922	77.72995	0.187981	4.135572	5.827396	1.127883	7.801192	1.503844	103.6713	2.443747	0.281971	33.46053	0.09399	0.657932	0.375961	0.563942	1.127883	17.3882	2.161776	41.26173	3.101679	6.955279	2.913698	9.962968	6.673309	23.87353	0.09399	130.6465	1.785815	2.349757	0.187981
	1	2	3	4	5	6	7	8	9	10	11	12	13	14	15	16	17	18	19	20	21	22	23	24	25	26	27	28	29	30	31	32	33	34	35

Table S11. Frequencies of dual-sgRNA cassettes in *E. coli* Gly-L1 library.

24	10.51525	5.841804	4.089263	26.28812	12.85197	9.931067	17.52541	12.26779	43.22935	18.69377	4.673443	4.089263	42.64517	0	30.96156	12.85197	12.26779	17.52541	11.09943	54.32878	21.03049	18.69377	30.96156	8.178526
23	25.11976	9.931067	14.60451	48.48697	19.86213	16.94123	18.69377	18.10959	54.91296	44.39771	14.60451	11.68361	83.5378	0	59.00222	8.762706	34.46664	19.86213	15.77287	64.84402	19.86213	8.762706	46.73443	30.37738
22	16.35705	7.010165	2.920902	11.68361	7.594345	5.257624	9.931067	14.02033	15.77287	35.635	6.425984	6.425984	28.04066	0	22.78304	4.673443	12.26779	8.762706	9.346886	27.45648	10.51525	4.673443	15.77287	1.752541
21	5.257624	2.920902	1.168361	9.346886	9.346886	6.425984	9.346886	4.673443	11.09943	13.43615	8.178526	2.336722	8.762706	0.58418	8.178526	3.505082	2.336722	3.505082	3.505082	9.931067	9.931067	7.594345	7.010165	5.257624
20	31.54574	15.18869	4.089263	38.55591	23.36722	23.36722	24.53558	20.44631	65.4282	42.64517	9.346886	8.762706	59.5864	0	42.64517	16.35705	29.7932	26.28812	17.52541	42.64517	29.20902	23.9514	39.14009	12.85197
19	5.841804	4.673443	3.505082	12.85197	9.346886	6.425984	7.010165	4.673443	23.9514	16.35705	4.673443	1.752541	19.86213	0	21.61467	4.089263	13.43615	9.931067	4.673443	15.77287	5.257624	5.257624	6.425984	14.02033
18	4.089263	1.752541	0.58418	5.257624	4.673443	4.673443	2.920902	2.920902	6.425984	8.762706	4.673443	2.336722	9.931067	0	20.44631	0.58418	1.168361	1.168361	1.168361	7.010165	10.51525	2.336722	6.425984	1.168361
17	29.7932	12.26779	6.425984	46.73443	19.86213	25.70394	20.44631	21.03049	35.635	52.57624	12.85197	7.010165	53.7446	0	47.31861	7.010165	20.44631	19.86213	22.78304	47.31861	19.86213	15.77287	35.05082	20.44631
16	5.841804	2.336722	0	5.841804	7.010165	7.010165	3.505082	5.841804	4.673443	15.18869	3.505082	0.58418	10.51525	0	5.257624	3.505082	3.505082	3.505082	3.505082	10.51525	3.505082	5.841804	15.77287	4.673443
15	12.85197	18.10959	4.089263	23.9514	13.43615	13.43615	11.09943	13.43615	31.54574	21.03049	24.53558	10.51525	37.97173	0.58418	23.9514	8.178526	17.52541	21.03049	11.68361	34.46664	16.35705	16.35705	32.7141	12.85197
14	19.86213	13.43615	9.346886	40.30845	30.37738	13.43615	18.69377	12.85197	29.7932	43.22935	9.346886	8.762706	37.38755	0	37.38755	12.26779	29.7932	22.78304	16.35705	58.41804	23.36722	11.09943	25.11976	18.10959
13	36.21918	29.20902	12.85197	48.48697	40.89263	32.12992	22.19886	36.21918	71.27001	60.17058	9.931067	7.594345	108.6576	0.58418	61.92312	13.43615	43.81353	35.635	21.03049	114.4994	42.64517	14.02033	68.93329	32.7141
12	4.673443	4.673443	0	5.257624	7.010165	8.178526	1.752541	3.505082	5.841804	3.505082	1.752541	0	8.762706	0	14.60451	3.505082	4.673443	11.09943	1.752541	9.931067	8.762706	3.505082	4.089263	1.168361
11	7.594345	1.752541	2.336722	5.257624	17.52541	1.168361	2.920902	7.010165	5.841804	4.673443	2.336722	4.673443	11.09943	0	3.505082	2.336722	1.752541	4.673443	4.673443	26.28812	7.010165	2.336722	11.68361	5.257624
10	22.19886	11.68361	7.010165	28.62484	19.27795	16.35705	18.69377	12.85197	33.29828	25.11976	9.346886	15.18869	63.67566	0.58418	44.98189	12.26779	25.70394	28.04066	14.60451	66.59657	16.94123	19.86213	39.72427	12.26779
9	1.168361	0.58418	0.58418	2.920902	4.673443	4.673443	0.58418	0	0.58418	2.920902	0.58418	0	4.673443	0	1.752541	0.58418	1.168361	1.168361	1.752541	2.336722	3.505082	1.752541	5.257624	1.168361
8	12.26779	8.762706	3.505082	22.78304	22.19886	12.85197	11.09943	9.346886	24.53558	26.8723	15.77287	5.257624	41.47681	0	21.61467	6.425984	31.54574	23.36722	14.02033	35.05082	23.36722	8.178526	23.36722	9.346886
7	7.594345	10.51525	1.168361	15.77287	15.18869	4.673443	12.26779	5.841804	14.60451	18.69377	2.336722	5.257624	24.53558	0	11.68361	5.257624	13.43615	4.673443	13.43615	22.78304	6.425984	2.920902	9.931067	5.841804
6	12.26779	13.43615	3.505082	26.8723	21.03049	11.09943	12.85197	11.09943	23.36722	27.45648	2.920902	8.178526	51.99206	0	27.45648	8.762706	14.02033	11.09943	17.52541	40.30845	9.346886	14.02033	14.60451	15.77287
5	44.98189	24.53558	8.178526	59.5864	18.10959	16.35705	28.62484	36.80336	78.28017	65.4282	30.96156	10.51525	80.61689	0.58418	73.60673	15.18869	42.64517	34.46664	28.62484	103.3999	36.21918	25.11976	65.4282	33.29828
4	38.55591	22.19886	6.425984	33.29828	32.7141	16.35705	26.8723	32.12992	36.21918	36.21918	12.26779	17.52541	80.03271	0.58418	54.32878	20.44631	37.38755	41.47681	25.70394	75.94345	35.05082	19.27795	77.69599	36.80336
3	8.762706	8.178526	3.505082	17.52541	9.346886	12.26779	8.762706	9.346886	32.12992	14.02033	5.841804	6.425984	43.81353	0	26.28812	3.505082	20.44631	16.35705	9.931067	33.88246	14.02033	8.762706	16.94123	9.931067
2	11.09943	6.425984	9.346886	18.10959	8.762706	10.51525	26.8723	10.51525	33.29828	35.05082	7.010165	4.089263	36.80336	0	30.96156	7.010165	22.19886	18.69377	21.03049	41.47681	16.94123	13.43615	25.11976	14.60451
1	13.43615	8.178526	0	17.52541	18.69377	8.178526	13.43615	7.594345	14.02033	9.346886	5.257624	5.841804	41.47681	0.58418	25.70394	11.09943	16.35705	6.425984	16.35705	34.46664	12.26779	8.178526	20.44631	12.85197
	1	2	3	4	5	6	7	8	9	10	11	12	13	14	15	16	17	18	19	20	21	22	23	24

Table S12. Frequency of the different dual-sgRNA cassettes of Gly-L2 library in *Vibrio* FA2 containing dCas9 before inducing.

24	11.54856	1.049869	1.049869	2.099738	0	1.049869	0	1.049869	59.84252	1.049869	0	1.049869	88.18898	1.049869	39.89501	0	5.249344	6.299213	2.099738	208.9239	0	0	62.99213	5.249344
23	38.84514	2.099738	0	9.448819	11.54856	4.199475	4.199475	1.049869	153.2808	10.49869	1.049869	0	354.8556	1.049869	121.7848	1.049869	30.44619	23.09711	4.199475	449.3438	5.249344	0	124.9344	12.59843
22	2.099738	0	0	1.049869	0	0	0	0	4.199475	0	0	0	9.448819	0	3.149606	0	0	1.049869	0	12.59843	0	0	8.39895	2.099738
21	3.149606	0	0	0	2.099738	1.049869	0	0	3.149606	1.049869	1.049869	0	13.64829	0	6.299213	1.049869	0	2.099738	1.049869	16.7979	1.049869	0	4.199475	0
20	49.34383	2.099738	1.049869	5.249344	4.199475	8.39895	2.099738	0	103.937	8.39895	2.099738	1.049869	216.273	1.049869	95.53806	0	17.84777	12.59843	11.54856	275.0656	1.049869	0	152.231	9.448819
19	3.149606	0	0	1.049869	1.049869	0	0	0	9.448819	0	0	0	19.94751	0	13.64829	0	4.199475	1.049869	1.049869	34.64567	0	0	6.299213	0
18	5.249344	0	0	1.049869	1.049869	0	0	1.049869	9.448819	0	1.049869	0	40.94488	1.049869	18.89764	0	0	0	0	24.14698	10.49869	0	14.69816	1.049869
17	28.34646	3.149606	0	11.54856	4.199475	4.199475	2.099738	1.049869	40.94488	8.39895	0	0	123.8845	0	54.59318	0	13.64829	11.54856	6.299213	244.6194	2.099738	3.149606	82.93963	13.64829
16	0	0	0	1.049869	0	0	0	0	0	1.049869	0	1.049869	1.049869	0	0	1.049869	1.049869	0	0	7.349081	0	0	2.099738	0
15	20.99738	4.199475	0	2.099738	3.149606	0	1.049869	0	66.14173	5.249344	0	1.049869	187.9265	2.099738	50.3937	1.049869	19.94751	22.04724	5.249344	275.0656	2.099738	0	139.6325	10.49869
14	11.54856	1.049869	0	3.149606	2.099738	3.149606	0	0	35.69554	4.199475	0	0	83.9895	0	40.94488	0	5.249344	6.299213	3.149606	217.3228	5.249344	1.049869	36.74541	4.199475
13	43.04462	2.099738	1.049869	16.7979	13.64829	7.349081	2.099738	4.199475	131.2336	17.84777	0	2.099738	433.5958	0	97.6378	1.049869	20.99738	25.19685	14.69816	635.1706	6.299213	0	206.8241	13.64829
12	0	0	0	0	0	0	0	0	0	0	0	0	4.199475	0	2.099738	0	0	4.199475	0	4.199475	1.049869	0	1.049869	0
11	0	0	0	0	0	0	3.149606	0	1.049869	0	0	0	3.149606	0	2.099738	0	0	0	0	4.199475	0	0	0	0
10	8.39895	1.049869	0	3.149606	0	1.049869	0	0	39.89501	6.299213	0	0	111.2861	0	38.84514	0	7.349081	6.299213	1.049869	170.0787	1.049869	1.049869	53.54331	2.099738
9	3.149606	0	0	0	2.099738	3.149606	0	0	4.199475	0	0	0	29.39633	0	1.049869	0	1.049869	1.049869	1.049869	12.59843	0	1.049869	6.299213	0
8	5.249344	0	0	1.049869	0	2.099738	0	0	5.249344	0	3.149606	0	20.99738	0	3.149606	0	3.149606	2.099738	3.149606	40.94488	4.199475	0	4.199475	1.049869
7	8.39895	3.149606	0	1.049869	0	1.049869	0	0	11.54856	1.049869	0	0	39.89501	0	20.99738	1.049869	1.049869	2.099738	2.099738	296.063	0	0	19.94751	3.149606
6	8.39895	0	0	0	1.049869	2.099738	0	0	29.39633	7.349081	3.149606	0	64.04199	0	22.04724	0	3.149606	5.249344	1.049869	118.6352	0	0	16.7979	4.199475
5	7.349081	1.049869	0	1.049869	0	0	2.099738	1.049869	29.39633	4.199475	0	1.049869	61.94226	1.049869	34.64567	0	8.39895	6.299213	4.199475	117.5853	1.049869	0	46.19423	4.199475
4	12.59843	2.099738	1.049869	2.099738	7.349081	3.149606	1.049869	1.049869	33.5958	2.099738	1.049869	0	90.28871	0	37.79528	0	5.249344	18.89764	0	164.8294	2.099738	1.049869	57.74278	7.349081
3	0	0	0	1.049869	0	0	0	0	1.049869	0	0	0	13.64829	0	3.149606	1.049869	5.249344	2.099738	0	12.59843	0	0	5.249344	0
2	3.149606	0	0	3.149606	0	1.049869	0	0	29.39633	2.099738	0	0	49.34383	0	26.24672	0	4.199475	7.349081	3.149606	104.9869	1.049869	2.099738	39.89501	5.249344
1	19.94751	1.049869	0	1.049869	2.099738	1.049869	3.149606	0	50.3937	1.049869	3.149606	0	183.727	0	55.64304	0	10.49869	5.249344	1.049869	277.1654	3.149606	0	82.93963	6.299213
	1	2	3	4	5	6	7	8	9	10	11	12	13	14	15	16	17	18	19	20	21	22	23	24

Table S13. Frequency of the different dual-sgRNA cassettes of Gly-L2 library in *Vibrio* FA2 containing dCas9 after inducing.

24	0	0	0	1.47776	1.47776	0	0	0	29.55519	1.47776	0	0	63.54367	0	25.12192	0	8.866558	4.433279	1.47776	127.0873	1.47776	0	42.85503	1.47776
23	14.7776	4.433279	0	1.47776	5.911039	2.955519	2.955519	1.47776	79.79902	7.388799	0	1.47776	223.1417	0	107.8765	0	17.73312	25.12192	5.911039	421.1615	5.911039	0	82.75454	7.388799
22	0	0	0	0	0	0	0	0	0	0	0	0	1.47776	0	0	0	0	1.47776	0	1.47776	0	1.47776	1.47776	1.47776
21	1.47776	0	0	0	0	0	0	0	7.388799	1.47776	0	0	8.866558	0	5.911039	0	1.47776	0	0	11.82208	0	0	4.433279	4.433279
20	20.68864	4.433279	0	8.866558	1.47776	5.911039	2.955519	1.47776	144.8205	10.34432	0	0	316.2406	1.47776	143.3427	0	31.03295	31.03295	7.388799	338.407	7.388799	1.47776	149.2537	11.82208
19	1.47776	0	0	0	0	0	0	0	8.866558	0	0	0	14.7776	0	8.866558	0	8.866558	1.47776	1.47776	25.12192	1.47776	0	2.955519	1.47776
18	2.955519	0	0	1.47776	1.47776	2.955519	0	0	11.82208	2.955519	0	0	42.85503	0	17.73312	0	2.955519	2.955519	1.47776	44.33279	7.388799	0	14.7776	1.47776
17	14.7776	0	1.47776	10.34432	1.47776	4.433279	2.955519	0	47.28831	4.433279	0	0	156.6425	0	78.32126	1.47776	7.388799	11.82208	4.433279	242.3526	2.955519	0	57.63263	11.82208
16	2.955519	0	0	0	0	0	0	0	0	1.47776	0	0	1.47776	0	0	0	0	1.47776	0	1.47776	0	0	0	0
15	4.433279	2.955519	0	4.433279	0	4.433279	1.47776	0	82.75454	5.911039	0	0	161.0758	0	67.97695	0	16.25536	39.89951	4.433279	393.0841	1.47776	1.47776	137.4317	8.866558
14	1.47776	0	0	4.433279	2.955519	2.955519	1.47776	0	25.12192	1.47776	0	0	45.81055	0	35.46623	0	8.866558	8.866558	2.955519	118.2208	0	0	19.21088	8.866558
13	20.68864	8.866558	1.47776	13.29984	8.866558	2.955519	4.433279	1.47776	150.7315	13.29984	1.47776	0	413.7727	1.47776	150.7315	2.955519	28.07743	36.94399	17.73312	849.7118	10.34432	0	226.0972	32.51071
12	0	0	0	0	0	0	0	0	0	0	0	0	1.47776	0	0	0	0	0	0	4.433279	0	0	0	0
11	0	0	0	0	0	0	0	0	1.47776	0	0	0	0	0	1.47776	0	1.47776	0	0	4.433279	0	0	0	0
10	4.433279	1.47776	0	4.433279	2.955519	2.955519	0	0	44.33279	5.911039	1.47776	0	70.93247	0	48.76607	0	7.388799	5.911039	2.955519	190.631	1.47776	0	48.76607	2.955519
9	0	0	0	0	0	1.47776	0	0	2.955519	0	0	0	44.33279	0	5.911039	0	1.47776	8.866558	0	32.51071	1.47776	0	13.29984	2.955519
8	4.433279	0	0	0	0	0	0	0	2.955519	0	4.433279	0	22.1664	0	8.866558	0	2.955519	4.433279	0	19.21088	0	0	7.388799	1.47776
7	8.866558	2.955519	0	2.955519	0	2.955519	0	0	48.76607	7.388799	0	0	137.4317	0	50.24383	0	5.911039	14.7776	4.433279	817.2011	5.911039	0	47.28831	2.955519
6	4.433279	0	0	0	1.47776	0	1.47776	1.47776	26.59967	1.47776	0	0	73.88799	0	10.34432	0	2.955519	16.25536	1.47776	94.57662	0	0	8.866558	5.911039
5	11.82208	2.955519	0	0	0	0	1.47776	0	20.68864	0	0	0	45.81055	0	20.68864	0	4.433279	4.433279	0	109.3542	0	0	19.21088	5.911039
4	2.955519	2.955519	0	4.433279	2.955519	1.47776	0	1.47776	32.51071	2.955519	0	0	62.06591	0	28.07743	0	2.955519	25.12192	1.47776	125.6096	0	0	29.55519	0
3	0	0	0	0	0	0	0	1.47776	0	0	0	0	1.47776	0	4.433279	0	0	1.47776	0	11.82208	0	0	1.47776	1.47776
2	4.433279	0	0	2.955519	1.47776	2.955519	0	0	26.59967	1.47776	0	0	28.07743	0	23.64416	1.47776	8.866558	7.388799	1.47776	82.75454	1.47776	0	25.12192	1.47776
1	2.955519	0	0	2.955519	0	0	0	0	23.64416	4.433279	0	0	76.84351	0	22.1664	0	2.955519	2.955519	0	159.598	1.47776	0	53.19935	2.955519
	1	2	3	4	5	6	7	8	9	10	11	12	13	14	15	16	17	18	19	20	21	22	23	24

Table S14. Frequency of the dual-sgRNA cassettes of HK-L2 library in dCas9-containing *Vibrio* FA2 cultivated without ampicillin (control, 1-CT-1).

35	0	0	0	0	0	0.256891	0	0	0	0	0	0	0	0	0	0.256891	0	0	0	0	0	0.770673	0	0.256891	0	0	0	0	0	0.256891	0	0.256891	0	0	0
34	3.596475	2.31202	0	133.8403	0.513782	1465.821	1.027564	5.651604	97.36173	2.055129	26.97357	1.284456	15.67036	5.394713	1.284456	77.32422	2.568911	0	46.75418	3.082693	2.568911	139.235	1.798238	163.3827	14.12901	1.027564	4.880931	4.880931	21.57885	10.53254	7.706733	272.0477	0.770673	8.991189	3.596475
33	1.027564	0	0	11.04632	0.513782	38.27677	0	1.284456	10.01875	0	5.137822	0	8.220515	0.770673	0	13.87212	0.513782	0	0.770673	0	2.055129	6.93606	0	22.60642	7.192951	0	0.770673	0.256891	2.055129	1.541347	0.770673	35.70786	0.256891	1.027564	0.256891
32	1.027564	0.256891	0	30.57004	0.513782	521.7458	0.513782	2.825802	30.05626	0.256891	4.110258	0.513782	12.33077	0.513782	0	43.4146	1.027564	0.256891	2.568911	0.770673	1.798238	7.706733	0.256891	59.34184	3.853367	0.770673	1.027564	0.513782	5.908495	3.082693	2.055129	29.28559	0	5.651604	0.256891
31	0	0	0	0.770673	0	3.339584	0	0	1.027564	0	0.513782	0	0	0	0	0.513782	0	0	0.513782	0	0.770673	0.256891	0	1.027564	0	0	0	0	0.256891	0	0	2.055129	0	0.770673	0
30	0.256891	0.256891	0	16.18414	0	98.90307	0	0.256891	24.40465	0.256891	3.339584	0.770673	2.055129	0.770673	0	18.49616	0.770673	0.256891	2.31202	0.513782	0.513782	6.422278	0	414.8791	3.082693	0	1.541347	1.027564	4.110258	1.798238	0.770673	50.60755	0.513782	4.880931	0
29	4.110258	1.284456	0	65.76412	3.339584	525.3423	1.798238	2.568911	55.23159	5.394713	15.92725	0	16.18414	1.027564	0.513782	92.22391	1.541347	0.770673	5.137822	1.284456	4.110258	25.946	2.055129	102.2427	7.706733	0.770673	3.082693	3.853367	12.33077	5.908495	8.477406	220.6695	1.798238	8.734298	0.770673
28	0	0.256891	0	3.339584	0	35.19408	0	0.256891	1.541347	0	0	0.256891	0.513782	0	0	2.568911	0	0	0	0	0.256891	1.027564	0	4.367149	1.284456	0	0.256891	0.256891	0.513782	0.513782	0	5.908495	0.256891	0.513782	0
27	2.568911	0	0	31.8545	0.256891	195.4941	0.770673	1.541347	40.07501	1.027564	5.137822	1.284456	8.734298	0.256891	0	43.92838	0.513782	0.513782	4.110258	0.513782	1.027564	11.04632	0.770673	75.52598	5.651604	0	1.284456	3.082693	8.220515	4.62404	1.541347	693.606	0.256891	10.27564	1.284456
26	0.256891	0	0	2.055129	0	13.61523	0	0.256891	1.027564	1.027564	0.513782	0	1.284456	0.513782	0	2.568911	0.256891	0	0	0	0	2.055129	0.256891	2.055129	1.284456	0	0.513782	0	0.513782	0	0.770673	3.596475	0	0.256891	0.256891
25	0.256891	0.256891	0	10.27564	0.513782	31.34071	0	0.256891	6.422278	0	0.256891	0	1.284456	0	0	11.81699	0	0	1.541347	0	0	2.568911	0	84.77406	0	0.256891	0	0.513782	1.798238	2.31202	0.513782	15.15658	1.027564	1.541347	0
24	0.256891	0.256891	0	9.504971	0	54.46091	0	0.513782	11.30321	0.256891	1.027564	0	7.706733	0	0	11.5601	0.513782	0	0.770673	0.256891	0.513782	2.825802	0.513782	17.4686	1.798238	0.256891	1.284456	2.568911	1.027564	3.339584	0	63.4521	0.256891	0.513782	0.256891
23	0.256891	0	0	3.596475	0	7.706733	0	1.798238	1.284456	0	0.256891	1.027564	0.513782	0	0	5.651604	0.256891	0	0.256891	0	0.256891	0.770673	0	3.596475	0.256891	0	0	0.256891	0.770673	0.256891	0.256891	4.62404	0	0.256891	0
22	0.770673	0.770673	0.256891	8.991189	0.256891	46.49729	0.256891	0.770673	11.5601	0.256891	0.513782	0.256891	2.825802	0.770673	0	36.47854	0.256891	0.513782	1.541347	0	1.284456	1.798238	0.513782	24.40465	1.027564	1.027564	1.027564	0.770673	2.31202	1.541347	0.770673	27.74424	0	1.027564	0.770673
21	0	0	0	0.770673	0	1.798238	0	0	0.513782	0	0	0	0	0	0	1.284456	0	0	0	0	0	0.256891	0	0.513782	0	0	0	0	0	0	0.256891	0.256891	0	0.256891	0
20	0	0	0	3.596475	0.256891	5.137822	0	0.256891	1.541347	0.513782	0.256891	0	0.256891	0	0	3.339584	0	0	0.256891	0.256891	0	0.770673	0	3.082693	0.256891	0	0	0.513782	0	1.027564	0	4.62404	0	0	0
19	0	0	0	2.568911	0	6.679169	0	0	1.027564	0	0	0	0.513782	0.513782	0	0.770673	0	0	0.513782	0	0	0.513782	0	2.825802	0	0	0.256891	0	0.256891	0	0	0.770673	0	0.256891	0
18	0	0	0	0.256891	0	0.770673	0	0.513782	0.256891	0	0.256891	0	0	0	0	0	0	0	0	0	0	1.284456	0	0.770673	0	0	0	0	0.513782	0.256891	0	1.541347	0	0	0
17	0	0	0	0	0	0.770673	0	0	0.256891	0	0	0	0	0	0	0	0	0	0	0	0	0	0	0	0	0	0	0	0	0	0	0	0	0	0
16	1.798238	0.256891	0	42.90081	0.770673	617.0524	0.513782	3.596475	153.364	1.541347	10.01875	0.513782	15.67036	2.055129	0.256891	25.43222	1.027564	1.027564	3.339584	1.027564	2.055129	14.89968	0.770673	145.9141	25.17533	0.256891	4.110258	5.394713	3.853367	4.62404	3.853367	177.5118	1.027564	7.449842	1.541347
15	0	0	0	0	0	0	0	0	0.256891	0	0	0	0	0	0	0	0	0	0	0	0	0.256891	0	0	0	0	0	0	0	0	0	0	0	0	0
14	0	0.256891	0	0.770673	0	1.284456	0	0	0.513782	0	0.256891	0	0	0	0	2.568911	0	0	0.256891	0	0	0	0	6.165386	0	0	0.256891	0	0	0.256891	0	2.825802	0	0	0
13	2.568911	0	0	28.7718	0.256891	75.78288	0	2.055129	13.10145	0.770673	5.651604	0.770673	1.798238	1.284456	0	18.23927	0.256891	0	1.027564	0	0.256891	6.422278	0.513782	24.91844	22.60642	0.256891	1.541347	2.055129	1.798238	17.2117	1.284456	51.37822	0	2.31202	1.541347
12	0	0	0	0.513782	0	1.541347	0	0	0.256891	0	0	0	0	0	0	1.284456	0	0	0	0	0	0.513782	0	1.798238	0.256891	0	0	0	0	0	0	0.513782	0	0	0
11	0	0	0	6.679169	0.256891	23.1202	0.256891	0	3.853367	0	0	0	0.513782	0	0	10.53254	0	0	0.256891	0	0.513782	4.62404	0	17.4686	1.284456	0.256891	0.513782	0.256891	0.513782	8.734298	0	12.07388	0	2.568911	0
10	0	0	0	1.798238	0	4.367149	0	0	1.541347	0	0.513782	0	0.513782	0	0	2.055129	0	0	0.256891	0	0	0	0	1.284456	0	0	0	0.256891	0	0	0	2.055129	0	1.284456	0
9	0.513782	0	0	21.57885	0	26.71667	0.513782	0.513782	4.367149	0.256891	2.31202	0.256891	2.31202	1.284456	0	30.31315	0	0	10.78943	0	0.513782	6.93606	0.256891	15.15658	0.513782	0	0.770673	1.284456	0.513782	0	0	19.52372	0	1.541347	0
8	0	0	0	1.798238	0.256891	14.3859	0	0	1.541347	0	0	0	0	0	0	2.568911	0	0	0.513782	0	0	0.513782	0	1.541347	0	0	0	0	0.256891	0.770673	0	10.01875	0	0.256891	0
7	0	0	0	0.256891	0	1.027564	0	0	0.256891	0.256891	0.256891	0	0	0	0	1.284456	0	0	0.256891	0	0	1.027564	0	0.770673	0	0	0	0	0	0	0	1.027564	0	0	0
6	0.256891	0	0	12.58766	0.256891	39.30434	0.256891	0.770673	17.2117	0.256891	2.055129	0.256891	3.339584	0	0	14.89968	0.770673	0.256891	2.31202	0	0.770673	3.339584	0.256891	235.3123	1.798238	0	0.513782	1.027564	3.339584	2.568911	0	42.38703	0	5.394713	0.513782
5	0	0	0	0.770673	0	3.853367	0	0	1.541347	0	0.256891	0	0	0.513782	0	2.568911	0	0	0	0	0	0.256891	0	3.339584	0.256891	0	0.256891	0.256891	0.256891	0	0	1.798238	0	0.256891	0.256891
4	6.93606	0.513782	0	21.57885	0	64.47967	0	0	14.89968	0.513782	1.798238	0.256891	5.394713	0.256891	0	35.45097	0	0	1.284456	0.513782	0	4.62404	0	24.40465	1.798238	0.256891	0	1.284456	2.055129	2.055129	0.770673	137.4367	0	4.367149	0
3	0	0	0	0	0	0.770673	0	0	0	0	0	0	0	0	0	0	0	0	0	0	0	0.256891	0	0	0	0	0	0	0	0	0	0	0	0	0
2	0	0	0	1.541347	0	5.651604	0	0.256891	0.513782	0	0.256891	0	0.256891	0	0	4.880931	0	0	0	0	0	0.256891	0	1.798238	0.256891	0	0	0.256891	0	0.256891	0	7.706733	0	0.256891	0
1	0.770673	0	0	5.137822	0	19.26683	0.256891	0.513782	2.055129	0	1.284456	0	1.027564	0	0	4.880931	0.256891	0	0	0	0	3.596475	0.513782	5.137822	1.027564	0.256891	0	0.770673	1.027564	1.798238	0	24.66155	0	0.256891	0.256891
	1	2	3	4	5	6	7	8	9	10	11	12	13	14	15	16	17	18	19	20	21	22	23	24	25	26	27	28	29	30	31	32	33	34	35

Table S15. Frequency of the dual-sgRNA cassettes of HK-L2 library in dCas9-containing *Vibrio* FA2 cultivated without ampicillin (control, 1-CT-2).

35	0	0	0	0	0	0	0	0	0	0	0	0	0	0	0	0	0	0	0	0	0	0	0	0	0	0	0	0	0	0	0	0	0	0	0
34	1.341742	5.366966	0	114.048	0	640.0107	1.341742	9.392191	32.2018	5.366966	22.80961	4.025225	36.22702	6.708708	1.341742	147.5916	2.683483	1.341742	18.78438	1.341742	1.341742	285.791	9.392191	116.7315	6.708708	1.341742	2.683483	8.050449	40.25225	37.56876	8.050449	542.0636	2.683483	6.708708	2.683483
33	0	0	0	25.49309	1.341742	73.79579	0	6.708708	6.708708	0	8.050449	0	26.83483	0	0	46.96096	0	0	0	0	0	14.75916	0	48.3027	6.708708	1.341742	1.341742	0	2.683483	8.050449	4.025225	95.26365	0	2.683483	0
32	0	0	0	45.61921	0	249.5639	0	8.050449	6.708708	1.341742	5.366966	0	38.91051	0	1.341742	50.98618	0	1.341742	0	0	1.341742	29.51831	0	67.08708	0	0	2.683483	0	1.341742	5.366966	2.683483	63.06185	0	5.366966	0
31	0	0	0	2.683483	0	1.341742	0	0	1.341742	0	0	0	2.683483	0	0	0	0	0	0	0	0	1.341742	0	1.341742	0	1.341742	0	0	0	0	0	5.366966	0	5.366966	0
30	0	0	0	16.1009	0	33.54354	1.341742	1.341742	0	0	2.683483	1.341742	1.341742	0	0	21.46787	0	0	1.341742	0	1.341742	4.025225	0	287.1327	0	0	0	0	4.025225	1.341742	0	60.37837	1.341742	1.341742	1.341742
29	12.07567	4.025225	0	95.26365	1.341742	405.206	2.683483	2.683483	28.17657	10.73393	17.44264	0	42.93573	0	1.341742	151.6168	1.341742	0	2.683483	2.683483	8.050449	53.66966	1.341742	161.009	8.050449	1.341742	4.025225	9.392191	12.07567	33.54354	12.07567	470.9513	1.341742	6.708708	1.341742
28	0	0	0	2.683483	0	16.1009	0	0	0	0	0	0	2.683483	0	0	4.025225	0	0	0	0	0	0	0	4.025225	0	0	0	0	0	0	0	12.07567	0	1.341742	0
27	1.341742	0	0	38.91051	0	162.3507	0	1.341742	17.44264	0	6.708708	1.341742	22.80961	1.341742	0	55.0114	0	0	0	1.341742	1.341742	36.22702	1.341742	75.13753	2.683483	6.708708	0	1.341742	5.366966	10.73393	1.341742	865.4233	0	1.341742	1.341742
26	0	0	0	6.708708	0	21.46787	0	1.341742	1.341742	0	0	0	2.683483	1.341742	0	6.708708	0	0	0	0	0	1.341742	0	2.683483	1.341742	0	0	0	1.341742	0	0	13.41742	0	0	2.683483
25	0	0	0	5.366966	0	18.78438	0	0	1.341742	0	0	0	1.341742	0	0	10.73393	0	0	0	0	0	0	0	22.80961	0	1.341742	1.341742	0	1.341742	1.341742	0	10.73393	1.341742	0	0
24	1.341742	0	0	25.49309	0	57.69489	0	0	8.050449	0	4.025225	0	25.49309	0	0	36.22702	0	0	2.683483	1.341742	1.341742	5.366966	2.683483	25.49309	1.341742	0	4.025225	1.341742	5.366966	10.73393	0	220.0456	1.341742	0	1.341742
23	0	0	0	2.683483	0	13.41742	0	1.341742	0	0	1.341742	0	1.341742	0	0	2.683483	0	0	0	0	0	2.683483	0	4.025225	0	0	0	0	0	0	0	14.75916	0	0	0
22	0	0	0	5.366966	2.683483	52.32792	0	0	1.341742	1.341742	4.025225	0	10.73393	0	0	28.17657	0	0	0	0	0	4.025225	0	34.88528	1.341742	0	1.341742	0	6.708708	9.392191	2.683483	75.13753	0	0	0
21	0	0	0	0	0	8.050449	0	0	0	0	0	0	0	0	0	0	0	0	0	0	0	0	0	0	0	0	0	0	0	0	0	2.683483	0	0	0
20	0	0	0	4.025225	0	4.025225	0	0	0	0	0	0	0	0	0	8.050449	0	0	0	0	0	0	0	2.683483	0	0	0	2.683483	0	2.683483	0	12.07567	0	0	0
19	0	0	0	5.366966	0	8.050449	0	0	0	0	2.683483	0	1.341742	0	0	1.341742	0	0	0	0	0	2.683483	0	5.366966	0	0	0	0	0	0	0	6.708708	0	0	0
18	0	0	0	0	0	1.341742	0	0	0	0	0	0	0	0	0	0	0	0	0	0	0	4.025225	0	2.683483	0	0	0	1.341742	0	0	0	1.341742	0	0	0
17	0	0	0	0	0	0	0	0	0	0	0	0	0	0	0	0	0	0	0	0	0	0	0	0	0	0	0	0	0	0	0	0	0	0	0
16	0	0	0	50.98618	0	230.7796	0	4.025225	36.22702	0	10.73393	0	75.13753	1.341742	0	57.69489	1.341742	0	1.341742	2.683483	2.683483	30.86006	4.025225	258.9561	9.392191	0	4.025225	5.366966	13.41742	13.41742	8.050449	234.8048	0	1.341742	1.341742
15	0	0	0	0	0	0	0	0	0	0	0	0	0	0	0	0	0	0	0	0	0	1.341742	0	0	0	0	0	0	0	0	0	0	0	0	0
14	0	0	0	0	0	0	0	0	1.341742	0	0	0	0	0	0	1.341742	0	0	0	0	0	1.341742	0	2.683483	0	0	0	0	0	0	0	1.341742	0	0	0
13	1.341742	0	0	17.44264	1.341742	162.3507	0	5.366966	12.07567	0	20.12612	0	6.708708	0	0	118.0733	0	0	0	2.683483	0	32.2018	0	55.0114	10.73393	1.341742	0	1.341742	5.366966	22.80961	5.366966	177.1099	1.341742	2.683483	4.025225
12	0	0	0	1.341742	0	0	0	0	0	0	0	0	0	0	0	0	0	0	0	0	0	0	0	4.025225	0	0	1.341742	0	0	0	0	1.341742	0	0	0
11	0	0	0	14.75916	0	22.80961	0	0	4.025225	2.683483	0	0	4.025225	0	0	16.1009	0	0	0	0	0	13.41742	0	20.12612	1.341742	0	2.683483	1.341742	0	4.025225	0	30.86006	0	0	0
10	0	0	0	5.366966	0	13.41742	0	0	0	0	1.341742	0	0	0	0	2.683483	0	0	0	0	0	0	0	2.683483	1.341742	0	0	0	1.341742	0	0	6.708708	0	0	0
9	0	0	0	12.07567	0	13.41742	0	0	0	0	1.341742	0	2.683483	0	0	13.41742	0	0	2.683483	0	1.341742	8.050449	0	6.708708	0	0	2.683483	0	1.341742	0	0	20.12612	0	1.341742	0
8	0	0	0	1.341742	0	9.392191	0	0	0	0	0	0	1.341742	0	0	1.341742	0	0	0	0	0	0	0	0	0	0	0	1.341742	0	1.341742	0	8.050449	0	0	0
7	0	0	0	1.341742	0	1.341742	0	0	0	0	1.341742	0	0	0	0	1.341742	0	0	0	0	0	1.341742	0	1.341742	0	0	0	0	0	0	0	1.341742	0	0	0
6	1.341742	0	0	9.392191	0	20.12612	0	2.683483	2.683483	0	2.683483	0	10.73393	0	0	13.41742	0	0	0	0	0	4.025225	0	57.69489	2.683483	0	0	0	2.683483	4.025225	0	26.83483	0	0	1.341742
5	0	0	0	0	0	1.341742	0	0	1.341742	0	0	0	0	0	0	4.025225	0	0	0	0	0	4.025225	0	4.025225	1.341742	0	0	0	0	0	0	1.341742	0	0	0
4	0	0	0	8.050449	0	28.17657	0	1.341742	4.025225	0	1.341742	0	5.366966	0	0	36.22702	1.341742	0	0	0	1.341742	6.708708	1.341742	9.392191	1.341742	1.341742	0	5.366966	2.683483	4.025225	0	56.35315	0	0	0
3	0	0	0	0	0	1.341742	0	0	0	0	0	0	0	0	0	0	0	0	0	0	0	0	0	0	0	0	0	0	0	0	0	0	0	0	0
2	0	0	0	4.025225	0	8.050449	0	0	1.341742	0	2.683483	0	1.341742	0	0	8.050449	0	0	0	0	0	0	0	2.683483	0	0	0	1.341742	0	1.341742	0	17.44264	0	0	0
1	0	0	0	8.050449	0	37.56876	0	1.341742	0	0	0	0	0	0	0	1.341742	0	0	0	0	0	5.366966	0	9.392191	0	0	0	0	0	6.708708	1.341742	34.88528	0	0	0
	1	2	3	4	5	6	7	8	9	10	11	12	13	14	15	16	17	18	19	20	21	22	23	24	25	26	27	28	29	30	31	32	33	34	35

Table S16. Frequency of the dual-sgRNA cassettes of HK-L2 library in dCas9-containing *Vibrio* FA2 cultivated without ampicillin (control, 1-CT-3).

35	0	0	0	0	0	0.349211	0	0	0	0	0	0	0	0	0	0	0	0	0	0	0	0	0	0	0	0	0	0	0	0	0.349211	0	0	0	0
34	2.444475	3.492108	0	145.2717	0.698422	1454.463	0.698422	4.190529	88.00112	5.238162	22.00028	3.492108	12.57159	6.984216	1.396843	75.77874	2.444475	0	49.58793	2.793686	2.095265	106.5093	4.190529	150.8591	13.96843	2.444475	5.238162	5.238162	15.71449	8.381059	6.984216	244.7968	1.047632	9.428691	5.936583
33	1.396843	0	0	19.90501	0.349211	35.6195	0.349211	5.936583	6.285794	0	4.53974	0.349211	9.07948	1.047632	0.349211	21.65107	0	0	1.396843	0	0	4.888951	0	27.23844	10.82553	0	1.396843	1.047632	2.444475	1.746054	1.396843	43.65135	0.349211	1.746054	0
32	1.396843	1.047632	0	28.9845	1.047632	519.9749	0.349211	2.095265	26.19081	1.746054	5.238162	0	11.87317	0.349211	0	39.46082	1.047632	0.349211	1.396843	0.349211	1.047632	4.53974	0.698422	58.66741	5.238162	0.349211	1.047632	1.396843	4.53974	3.492108	0	20.95265	0	4.53974	0.349211
31	0	0	0	1.047632	0	1.396843	0	0	1.396843	0	0	0	0.698422	0	0	0	0	0	0	0	0.349211	0.349211	0	1.047632	0	0.349211	0	0	0	0	0.349211	1.047632	0	2.444475	0
30	1.746054	0	0	17.80975	0	76.82637	0	0.698422	20.25423	0	4.190529	0	3.142897	0.349211	0	14.31764	0.349211	0	2.095265	0	0	4.190529	0	436.1643	2.095265	0.349211	1.047632	1.047632	4.190529	1.047632	0.349211	36.66713	0.698422	4.53974	0
29	5.936583	1.047632	0	71.58821	1.746054	536.737	0.698422	4.190529	50.63556	7.333426	16.41291	0.349211	22.34949	0.698422	0.349211	101.9695	1.047632	0	5.238162	1.396843	4.190529	22.6987	2.095265	102.3188	14.31764	2.095265	4.190529	4.190529	10.12711	8.031848	8.381059	222.4473	3.142897	9.777902	0.698422
28	0	0	0	1.746054	0	40.15924	0	0	2.095265	0	0.349211	0	1.047632	0	0	2.095265	0	0	0.698422	0	0	0.349211	0	8.381059	1.047632	0.349211	0	0.349211	0.349211	0	0	8.031848	0	0.349211	0
27	1.396843	0.349211	0	34.57187	0	182.6372	0	0.698422	45.74661	1.047632	6.984216	0	7.333426	1.396843	0	46.44503	1.396843	0	5.587373	1.396843	0.349211	10.47632	0.349211	79.27085	9.428691	0.698422	1.047632	2.793686	9.07948	3.142897	1.396843	673.2784	1.396843	5.587373	1.396843
26	0.349211	0	0	1.746054	0	15.01606	0	0.349211	1.396843	1.047632	0.698422	0	1.047632	0.698422	0	4.190529	0.349211	0	0	0	0.349211	0.698422	0.349211	6.984216	1.396843	0	0.349211	0	0.349211	0.349211	0.349211	6.635005	0	0	0.349211
25	0.698422	0	0	11.17475	0	28.9845	0	0	6.635005	0.349211	0.698422	0	0.349211	0.349211	0	9.777902	0	0	0	0	0.698422	2.793686	0.349211	82.41374	0.698422	0.349211	0	0.349211	0.698422	1.396843	0	8.381059	0.698422	1.047632	0
24	0.349211	0	0	13.61922	0	42.95293	0	0.349211	7.333426	0.349211	3.142897	0	9.428691	0	0	15.36527	0	0	0.349211	0.698422	0	2.095265	0	18.15896	4.190529	0	1.047632	2.793686	1.047632	2.793686	0.698422	71.93742	0.349211	2.793686	0
23	0	0	0	4.190529	0	10.12711	0	1.047632	1.047632	0	0.349211	1.047632	0.349211	0	0	5.587373	0	0	0	0	0.698422	1.047632	0	4.190529	0	0	0	2.095265	0	0.349211	0.349211	6.984216	0	0	0
22	0.349211	0.698422	0	4.190529	0	27.58765	0	1.047632	5.587373	0	0.349211	0	3.142897	0.698422	0.349211	28.28607	0	0	0.698422	0	0.349211	0.698422	0	20.95265	0	0.349211	1.047632	0	1.396843	1.396843	1.047632	23.04791	0	2.095265	0
21	0	0.349211	0	0	0	1.746054	0	0	0	0	0	0	0	0	0	2.444475	0	0	0	0	0	0	0	0.698422	0	0	0	0	0	0	0	0.349211	0	0	0
20	0	0	0	4.190529	1.047632	3.142897	0	0	0.698422	0	0.349211	0	0	0	0	1.396843	0	0	0	0	0	0	0	1.047632	0.349211	0	0	0	0.349211	2.444475	0	5.587373	0	0.349211	0
19	0	0	0	2.095265	0	3.492108	0	0.349211	0	0	0	0	0.349211	0	0	3.142897	0	0	0	0	0	1.396843	0	1.746054	0	0	0	0	0	0	0	1.396843	0	0.349211	0
18	0	0	0	0.698422	0	1.746054	0	0	0	0	0	0	0.349211	0	0	0.349211	0	0	0.349211	0	0	1.047632	0	1.047632	0	0	0	0	0.349211	0	0	0.698422	0	0	0
17	0	0	0	0.349211	0	0	0	0	0	0	0	0	0	0	0	0	0	0	0	0	0	0.349211	0	0	0	0	0	0	0	0	0	0	0	0	0
16	1.746054	0	0	49.93714	0	678.1673	0.349211	1.746054	147.367	1.746054	11.87317	0.349211	27.23844	1.396843	0.349211	29.33371	1.047632	1.396843	4.888951	1.746054	3.841319	15.36527	1.746054	169.018	35.96871	0.349211	3.492108	5.936583	8.031848	5.936583	3.492108	167.9704	1.047632	9.428691	0.349211
15	0	0	0	0	0	0	0	0	0.349211	0	0	0	0	0	0	0	0	0	0	0.349211	0	0	0	0	0	0	0	0.349211	0	0	0	0	0	0	0
14	0	0	0	0.349211	0	2.095265	0	0	0.349211	0	0.349211	0	0	0	0	3.841319	0	0	0	0	0	0.349211	0	6.635005	0	0	0	0	0.349211	0	0	1.047632	0	0	0
13	1.396843	0.349211	0	30.03213	0	74.3819	0	2.095265	12.22238	0.349211	5.587373	2.793686	2.095265	1.047632	0	22.6987	0.698422	0	0	0	0.698422	8.381059	0.349211	21.30186	22.6987	0	1.746054	2.444475	2.095265	20.25423	0.349211	49.93714	0	1.047632	2.095265
12	0	0	0	0.698422	0	1.746054	0	0	0.349211	0	0	0	0	0	0	1.746054	0	0	0	0	0	0	0	2.444475	0	0	0	0	0.349211	0	0	1.396843	0	0	0
11	0	0	0	6.984216	0	24.09554	0	0.349211	4.53974	0.698422	0.349211	0	0	0.349211	0	11.52396	0	0	0.698422	0	0.349211	2.444475	0.349211	13.61922	2.095265	0.349211	0.349211	0.349211	1.047632	9.07948	0	12.57159	0	4.53974	0
10	0.349211	0	0	2.793686	0	5.936583	0	0.698422	0.698422	0	1.396843	0	0.349211	0	0	1.047632	0	0	0	0	0	0.349211	0	0.698422	0.349211	0	0	0	0.349211	0	0	2.793686	0	0.698422	0
9	0.349211	0	0	16.41291	0	20.60344	0	0.698422	2.095265	0	4.53974	0	0.698422	1.396843	0	37.01634	0.349211	0.349211	10.12711	0.349211	0.349211	7.682637	0.349211	16.41291	0	0.349211	0.349211	1.047632	1.746054	0.698422	0.349211	22.00028	0.349211	1.047632	0.349211
8	0	0.349211	0	3.142897	0	12.9208	0	0	1.396843	0	0.349211	0	1.396843	0	0	1.746054	0	0	0.349211	0	0	0	0	3.142897	0	0	0	0	0	0.698422	0	8.381059	0	0.349211	0
7	0	0	0	0.698422	0	0	0	0	0	0	0	0	0	0	0	1.396843	0	0	0	0	0	1.047632	0	0.349211	0	0	0	0	0.349211	0	0	0.698422	0.698422	0	0
6	1.047632	0	0	9.07948	0.698422	38.06398	0	0.698422	14.66685	0.698422	2.095265	0	1.396843	0	0.349211	13.61922	0.349211	0	0.698422	0.349211	0	3.492108	0	249.6857	1.396843	0	0.349211	0.698422	2.444475	3.841319	0.698422	29.33371	0	3.841319	0
5	0	0	0	1.396843	0	6.984216	0	0	2.793686	0	0.698422	0	0	0.698422	0	1.746054	0.349211	0	0	0.349211	0	1.047632	0	3.492108	1.396843	0.349211	0	0	0	0	0	1.746054	0.349211	0.698422	0
4	5.587373	0	0	18.85738	0	60.76268	0	0.349211	12.22238	0	2.793686	0.698422	3.492108	1.047632	0	39.11161	0	0	1.396843	0	0	4.53974	0	25.49239	2.793686	0	0.349211	2.793686	1.746054	2.793686	0.698422	153.3035	0	1.396843	0
3	0	0.349211	0	0	0	0.698422	0	0	0.349211	0	0	0	0	0	0	0	0	0	0	0	0	0.349211	0	0.349211	0	0	0	0	0	0	0	0	0	0	0
2	0.349211	0	0	1.746054	0	3.492108	0	0.698422	0.349211	0	0.349211	0	1.047632	0	0	4.190529	0	0.698422	0	0	0	0.349211	0	2.444475	0.349211	0	0	0	0	0	0	9.428691	0	0.349211	0
1	0.349211	0	0	8.031848	0	18.50817	0	0.349211	3.492108	0	0.349211	0	0.349211	0	0	5.238162	0	0.349211	0.349211	0	0	3.142897	0.349211	3.841319	1.047632	0.349211	0.349211	0.349211	0.698422	1.746054	0	30.73055	0	0.698422	0
	1	2	3	4	5	6	7	8	9	10	11	12	13	14	15	16	17	18	19	20	21	22	23	24	25	26	27	28	29	30	31	32	33	34	35

Table S17. Frequency of the different dual-sgRNA cassettes of HK-L2 library in dCas9-containing *Vibrio* FA2 cultivated with 5 mg/mL of ampicillin (experiment, 1-Ex-1).

35	0	0	0	0	0	0.542123	0	0	0	0	0	0	0	0	0	0.271061	0	0	0	0	0	0	0	0.271061	0	0	0.271061	0	0	0	0	0	0	0	0
34	3.523799	5.42123	0	75.89721	2.710615	319.5815	0.542123	8.945029	16.80581	7.047598	25.75084	5.150168	64.78369	1.89743	2.710615	156.6735	1.626369	0.813184	3.523799	4.879107	2.168492	68.84962	4.879107	91.34772	12.19777	2.168492	6.776537	4.065922	28.73252	12.19777	6.776537	350.4825	2.168492	3.794861	7.860783
33	2.439553	0	0	32.79844	1.626369	131.4648	0	4.336984	5.150168	1.355307	13.28201	0	48.24894	0.813184	0.542123	90.53453	0.542123	0.271061	0.271061	1.355307	1.084246	11.38458	0.271061	60.17565	8.131844	1.89743	2.439553	1.89743	9.758213	4.608045	2.981676	151.2523	2.710615	4.065922	1.355307
32	2.168492	0.542123	0	31.71419	1.355307	156.6735	0.813184	4.879107	3.523799	3.523799	6.505475	0.271061	92.70303	0.813184	0.271061	65.59688	0.271061	1.084246	1.084246	0.813184	0.813184	18.16112	0.813184	71.83129	3.523799	1.355307	1.355307	1.355307	6.776537	4.608045	1.355307	45.26727	0.813184	2.981676	1.084246
31	0.271061	0	0	1.626369	0	7.589721	0	0.542123	1.084246	0	0	0	4.608045	0	0	3.523799	0	0	0	0	0	0.271061	0	2.710615	0.813184	1.355307	0.271061	0.271061	0.542123	0.542123	0	4.336984	0	2.710615	0
30	0.271061	0	0	3.794861	0	9.758213	0	1.084246	0.813184	0	1.89743	0	1.626369	0	0	11.9267	0	0	0	0	0.271061	1.89743	0	3.794861	0.542123	0	0.271061	0	0	0.813184	0.271061	11.38458	0.271061	0.542123	0
29	10.84246	3.252738	0	95.95576	5.42123	633.7417	1.89743	10.84246	27.64827	9.758213	30.90101	2.168492	140.6809	0.271061	1.89743	322.2921	2.439553	1.355307	4.065922	4.879107	7.589721	63.97051	5.963352	214.1386	14.90838	7.31866	8.673967	12.46883	18.16112	26.83509	14.90838	519.0827	6.234414	13.82414	5.150168
28	0	0	0	2.710615	0.542123	10.84246	0.271061	0.271061	0.542123	0	0.271061	0	1.084246	0	0	7.860783	0	0	0.271061	0	0	0.542123	0	4.336984	0.813184	0	0	0.542123	0.542123	0.271061	0.271061	12.19777	0.271061	0	0.271061
27	1.89743	0.813184	0	22.76916	0.542123	109.2378	0	4.879107	2.981676	0.813184	5.150168	0.813184	18.16112	0.271061	0	53.12805	0.813184	0	0.271061	1.626369	0.813184	14.36626	0.271061	57.73609	3.794861	1.084246	0.542123	2.168492	5.42123	7.31866	0.813184	110.322	0.271061	2.710615	0.271061
26	0.271061	0	0	7.31866	0	40.65922	0.271061	2.168492	0.542123	0.813184	1.355307	0	22.76916	0.542123	0.271061	21.41386	0	0	0	0	0.271061	7.31866	0	13.28201	0.813184	0	0.542123	0.271061	0.813184	1.084246	0.813184	18.16112	0	0.542123	2.710615
25	0.271061	0	0	8.402906	0.271061	30.08782	0	0	0.542123	0.542123	1.355307	0	4.879107	0.271061	0	15.17944	0.271061	0.271061	0	0.271061	0	1.355307	0.542123	19.51643	0.542123	1.084246	0.271061	0.271061	1.084246	3.252738	0.542123	13.01095	0.271061	0.271061	0
24	4.608045	0.271061	0	30.08782	0.271061	140.4098	0	3.523799	3.794861	1.626369	7.589721	0.271061	103.2744	0	0	64.51263	0.542123	0.542123	0.813184	0.813184	0.813184	8.945029	1.084246	44.72514	2.439553	1.084246	5.150168	6.776537	7.589721	9.758213	1.89743	213.3254	0.271061	2.981676	1.355307
23	0.542123	0	0	5.42123	0	22.4981	0	2.439553	0.271061	0	1.084246	0	4.879107	0	0	5.150168	0.271061	0	0	0	0.813184	1.89743	0	4.608045	0.813184	0.271061	0	1.626369	0.813184	0.813184	0	12.46883	0.542123	0.542123	0
22	0.813184	0.542123	0	8.131844	0.271061	39.03285	0	1.626369	3.794861	0.542123	3.794861	0	15.99263	0.271061	0.271061	25.75084	0.271061	0.542123	0.813184	0.271061	1.89743	3.252738	0.271061	24.12447	0.542123	0.271061	1.626369	2.168492	4.336984	4.065922	1.355307	52.0438	0	2.981676	0.271061
21	0	0	0	0.271061	0	1.355307	0	0.271061	0	0	0	0	0	0	0	2.710615	0	0	0	0	0	0	0	0.813184	0	0	0.271061	0	0	0	0	2.439553	0	0	0
20	0.542123	0	0	8.131844	0.271061	7.31866	0	0.813184	0.271061	0.271061	1.084246	0	3.794861	0	0	6.505475	0.271061	0	0	0	0.271061	0.813184	0	2.439553	0.271061	0	0	1.355307	1.084246	1.084246	0	8.673967	0	0.271061	0
19	0	0	0	3.523799	0	10.02927	0	0.542123	0.813184	0	1.084246	0	6.505475	0	0	6.234414	0	0	0	0	0.271061	3.523799	0	3.794861	0	0	0.271061	0.271061	1.084246	0.542123	0.271061	12.46883	0.271061	0.542123	0.271061
18	0.271061	0	0	0.271061	0	5.692291	0	0.271061	0	0	0.271061	0	1.084246	0	0	2.168492	0	0	0.271061	0.271061	0	2.168492	0	1.626369	0.542123	0	0	0	0	0	0.271061	3.252738	0	0	0.542123
17	0	0	0	0	0	0.542123	0	0	0	0	0.271061	0	0	0	0	0	0	0	0	0	0	0	0	0.542123	0	0	0.271061	0	0.271061	0	0	0.542123	0	0	0
16	6.234414	0.271061	0	42.82771	0.813184	222.5415	0.271061	4.879107	10.84246	3.252738	17.619	0.542123	83.21587	2.168492	0.542123	51.23062	0.542123	1.355307	1.626369	2.981676	4.608045	29.00358	2.168492	226.8785	7.047598	4.608045	2.168492	6.234414	9.21609	10.02927	4.065922	225.2521	2.439553	5.150168	4.879107
15	0	0	0	1.084246	0	1.084246	0	0	0	0	0	0	0	0	0	0.542123	0	0	0	0	0	0.271061	0	0.271061	0.271061	0	0.271061	0	0	0	0	0	0	0	0
14	0	0	0	1.084246	0	1.626369	0	0.542123	0	0	0	0	0	0	0	1.89743	0	0	0	0	0	0	0	1.626369	0.271061	0	0	0	0	0	0	3.252738	0	0.271061	0
13	2.168492	0.542123	0	46.35151	2.710615	350.2114	0.271061	18.70324	8.131844	11.65564	32.79844	2.981676	27.91933	1.355307	0.271061	272.6878	1.084246	0.271061	4.065922	2.439553	4.608045	32.52738	1.626369	85.65543	18.9743	1.355307	7.047598	5.150168	10.5714	20.05855	14.36626	303.8599	1.084246	6.234414	3.523799
12	0	0	0	0.813184	0	3.523799	0	0	0	0	0	0	0.271061	0	0	2.168492	0	0	0	0	0	0.271061	0	2.439553	0	0	0	0	0	0	0	4.608045	0	0	0
11	1.355307	0	0	13.28201	0.542123	49.33319	0.271061	1.626369	1.89743	0.813184	0.271061	0	8.673967	0	0	30.08782	0.813184	0	0.542123	0.271061	0.542123	14.0952	0.271061	34.15375	1.89743	0	1.084246	0.271061	1.626369	3.794861	0.813184	45.80939	0.271061	2.981676	0.542123
10	0	0	0	5.150168	0.271061	11.38458	0	0.542123	0.542123	0.271061	2.439553	0	2.168492	0	0	7.31866	0.271061	0	0	0	0.271061	0.271061	0	4.879107	0.271061	0	0	0.271061	0.813184	0.271061	0	9.21609	0	2.439553	0
9	0.271061	0.271061	0	9.487152	0.271061	16.80581	0	0.271061	0.813184	0	2.168492	0	5.963352	1.084246	0	13.55307	0	0	1.355307	0.271061	0.271061	8.945029	0	12.19777	0.271061	0.271061	1.355307	2.710615	2.439553	0.271061	0.271061	23.58235	0	0.813184	0
8	0	0	0	1.626369	0.813184	5.963352	0	0.271061	0	0	0.271061	0	0	0	0	3.794861	0	0.271061	0	0	0	1.355307	0	5.42123	0	0	0	0.271061	0.542123	0.813184	0	6.776537	0	0.271061	0
7	0	0	0	1.626369	0	0.813184	0	0.271061	0	0	0	0	0.542123	0	0	1.355307	0	0	0	0	0	0.813184	0	0.271061	0	0	0	0	0	0	0	0.813184	0	0	0
6	0.542123	0	0	8.945029	0.813184	67.49431	0	2.981676	2.439553	0.271061	2.981676	0	28.46146	0	0.271061	31.17207	0.542123	0.271061	0	0.542123	0.542123	4.879107	0.813184	25.20872	2.168492	1.355307	0.813184	0.271061	4.336984	1.355307	0.813184	45.26727	0	0.813184	0.271061
5	0	0	0	1.89743	0	6.776537	0	0.271061	1.084246	0	0.542123	0	0.813184	0.542123	0	5.692291	0	0	0	0.271061	0	0.271061	0	7.047598	0	0	0	0	0	0.542123	0	3.794861	0	0	0.813184
4	0.542123	0	0	7.31866	0.542123	30.08782	0	1.626369	0.813184	0.271061	3.252738	0	10.02927	0	0.542123	24.66659	0	0	0.271061	0.271061	0	3.252738	0.271061	13.01095	0	0.271061	0.813184	4.879107	0.542123	3.794861	0.542123	26.29296	0.271061	0.271061	0.271061
3	0	0	0	0	0	1.626369	0	0	0	0	0	0	0	0	0	0	0	0	0	0	0	0.271061	0	0.271061	0	0	0	0	0	0	0	0.271061	0	0	0
2	0	0	0	5.692291	0	7.31866	0	0.271061	0.271061	0.271061	0.813184	0	2.710615	0	0	3.794861	0	0.542123	0.271061	0	0.271061	2.439553	0.271061	3.252738	0.542123	0	0	0.813184	0.542123	0.813184	0	20.32961	0	0	0.542123
1	0.542123	0	0	11.11352	0.271061	79.42101	0.271061	3.523799	2.710615	0	4.065922	0	6.234414	0	0.271061	22.22704	0	0	0.542123	0.813184	1.084246	11.65564	0.271061	17.34793	2.710615	1.084246	0	0.271061	1.355307	8.402906	0.542123	50.6885	0	0.542123	0.542123
	1	2	3	4	5	6	7	8	9	10	11	12	13	14	15	16	17	18	19	20	21	22	23	24	25	26	27	28	29	30	31	32	33	34	35

Table S18. Frequency of the different dual-sgRNA cassettes of HK-L2 library in dCas9-containing *Vibrio* FA2 cultivated with 5 mg/mL of ampicillin (experiment, 1-Ex-2).

35	0	0	0	0	0	0	0	0	0	0	0	0	0	0	0	0.635445	0	0	0	0	0	0	0	0	0	0	0	0	0	0	0	0	0	0	0
34	1.906335	4.448116	0	90.86865	2.541781	583.3386	1.27089	10.16712	40.03304	5.083561	29.86592	5.719006	25.41781	3.812671	1.906335	134.0789	1.27089	0	12.07346	2.541781	4.448116	209.0614	1.906335	92.13954	13.97979	1.27089	9.531677	3.812671	37.49126	3.177226	12.07346	379.9962	1.27089	10.16712	9.531677
33	0.635445	0	0	27.32414	1.27089	74.34708	0	4.448116	10.80257	0.635445	6.989896	0.635445	20.33424	0	0	32.4077	1.27089	0	0	0	0.635445	9.531677	0	51.47106	17.79246	1.27089	0.635445	1.27089	6.989896	1.906335	2.541781	81.33698	0	1.906335	1.906335
32	3.812671	0	0	29.86592	0.635445	671.6655	0.635445	10.16712	19.06335	1.27089	7.625342	0.635445	55.28373	1.27089	0	74.98253	0	0	1.906335	0	1.27089	26.6887	0	61.00273	6.354451	1.27089	3.812671	0	6.989896	0	2.541781	48.29383	0.635445	5.083561	0
31	0	0	0	1.27089	0	4.448116	0	0	1.906335	0	0.635445	0	1.27089	0.635445	0	1.27089	0	0	0	0	0	0.635445	0	2.541781	0	0.635445	0.635445	0	0	0	0	4.448116	0.635445	3.812671	0
30	0	0	0	2.541781	0.635445	10.80257	0	0	0.635445	0	0	0.635445	0.635445	0	0	4.448116	0	0	0	0	0	0	0	15.88613	0	0	1.27089	0	0	0	0	3.812671	0	0	0
29	10.16712	2.541781	0	71.16985	11.43801	625.278	2.541781	8.260787	37.49126	12.7089	24.78236	1.906335	71.8053	1.27089	1.27089	221.7704	1.906335	1.27089	5.083561	3.177226	5.083561	56.55462	5.719006	130.9017	16.52157	1.27089	9.531677	2.541781	22.24058	6.354451	13.97979	559.8272	3.177226	10.80257	2.541781
28	0	0	0	2.541781	0	19.6988	0.635445	0	0.635445	0	0	0	0.635445	0	0	1.906335	0	0	0	0	0	0.635445	0	6.354451	1.906335	0	1.906335	0	0	0	0	5.719006	0	0	0
27	1.27089	0.635445	0	19.6988	0.635445	181.1019	0	3.177226	15.88613	1.906335	6.354451	1.27089	12.07346	0.635445	0	47.65838	1.906335	0	1.27089	0	0	20.33424	0	61.00273	6.989896	1.27089	0.635445	3.812671	10.16712	1.906335	0.635445	613.84	1.27089	1.906335	1.906335
26	0	0	0	3.812671	0	34.31404	0	0.635445	0.635445	1.906335	0	0	6.354451	1.27089	0	8.896232	0	0	0	0	0	5.719006	0	8.896232	2.541781	0	0	0.635445	0	0	3.177226	11.43801	0	0	1.906335
25	0	0	0	5.719006	1.27089	36.85582	0	0	1.906335	0	1.906335	0.635445	3.177226	0.635445	0.635445	22.24058	0.635445	0	0	0	0	2.541781	0	48.92927	0.635445	0	0	0	1.27089	0	0	10.16712	0	1.906335	0
24	2.541781	0	0	15.25068	1.906335	83.87876	0	0.635445	5.719006	0.635445	3.177226	0	31.13681	0.635445	0	26.6887	0	0	0	0	0.635445	6.989896	0	38.12671	3.812671	0	0	3.812671	2.541781	0.635445	1.27089	134.0789	0.635445	1.27089	0
23	0	0	0	4.448116	0	12.07346	0	4.448116	1.27089	0	0	0.635445	0	0	0	10.80257	0	0	0	0	1.27089	1.27089	0	2.541781	0	0.635445	1.27089	1.27089	0.635445	0	0	12.07346	0	0	0
22	0	0.635445	0	10.16712	1.27089	58.46095	0	1.906335	8.260787	0.635445	3.812671	0	8.260787	0	0.635445	23.51147	0.635445	0	1.27089	1.27089	0.635445	10.16712	0	20.96969	4.448116	1.27089	3.812671	1.27089	3.177226	0.635445	1.906335	56.55462	0	1.27089	0.635445
21	0	0	0	0	0	3.177226	0	0	1.27089	0	0	0	0	0	0	1.27089	0	0	0	0	0	0	0	1.27089	0.635445	0	0	0	0	0	0	1.27089	0	0	0
20	0	0	0	1.27089	0	5.083561	0	0	0	0	0	0	1.27089	0	0	1.27089	0	0	0	0	0.635445	1.27089	0	4.448116	0	0	0	0	0	0.635445	0	5.083561	0	0	0
19	0	0	0	2.541781	0.635445	5.719006	0	0	0.635445	0	0	0	0.635445	0	0	3.177226	0	0	0	0	1.27089	0.635445	0	1.27089	0.635445	0	0	0	0.635445	0	0	5.083561	0	0	0
18	0	0	0	1.27089	0	3.812671	0	0.635445	0	0	1.27089	0	0	0	0	0	0	0	0	0	0	0	0	0.635445	0	0	0	0	0.635445	0	0	1.906335	0	0	0.635445
17	0	0	0	0	0	0	0	0	0	0	0	0	0	0	0	0	0	0	0	0	0	1.27089	0	0	0	0	0	0	0	0	0	1.906335	0	0	0
16	3.177226	0	0	34.94948	0.635445	485.4801	1.27089	6.989896	144.246	1.906335	17.79246	4.448116	30.50137	2.541781	0	33.67859	1.27089	0	2.541781	0.635445	3.812671	26.6887	0.635445	155.0486	23.51147	2.541781	3.177226	4.448116	4.448116	4.448116	1.906335	251.6363	0	2.541781	3.812671
15	0	0	0	0	0	0	0	0	0	0	0	0	0	0	0	0	0	0	0	0	0	0	0	0	0	0	0	0	0	0	0.635445	0	0	0	0
14	0	0	0	1.27089	0	0.635445	0	0	0	0	0	0	0	0	0	1.906335	0	0	0	0	0	0	0	1.906335	0	0	0	0	0.635445	0	0	1.27089	0	0	0
13	2.541781	0	0	19.6988	0.635445	169.0284	1.906335	4.448116	5.719006	3.177226	15.25068	9.531677	10.16712	0.635445	0	81.97242	0.635445	0	1.27089	0.635445	0.635445	19.06335	0	34.94948	40.03304	0.635445	3.812671	2.541781	4.448116	1.906335	5.719006	126.4536	0	2.541781	8.260787
12	0	0	0	0.635445	0	2.541781	0	0	0	0	0.635445	0	0	0	0	2.541781	0	0	0	0	0	1.27089	0	6.354451	0	0	0	0	0	0.635445	0	3.812671	0	0	0
11	0.635445	0	0	13.34435	0.635445	40.03304	0	0.635445	3.177226	0.635445	0	0.635445	3.177226	0	0	22.87602	0	0	0	0	0	9.531677	0.635445	24.78236	3.812671	0	0.635445	0.635445	1.906335	1.27089	0	20.96969	0	1.906335	0
10	0	0.635445	0	1.906335	0	5.719006	0	0	1.27089	0	3.812671	0	1.27089	0.635445	0	2.541781	0	0	0	0	0	0.635445	0.635445	3.177226	0	0	0	0.635445	1.27089	0	0	1.27089	0	2.541781	0
9	0	0	0	13.97979	0	19.06335	0	0.635445	0.635445	0	4.448116	0	2.541781	1.27089	0	17.79246	0	0	10.16712	0	0	9.531677	0	6.989896	1.906335	0.635445	1.27089	0	1.27089	0	0	38.12671	0.635445	1.27089	0
8	0	0	0	1.906335	0	16.52157	0	0	1.27089	0	0.635445	0	0	0	0	3.177226	0	0	0	0	0	1.27089	0	6.354451	1.27089	0	0	0	0.635445	0.635445	0	13.97979	0	0.635445	0
7	0	0	0	0	0	1.27089	0	0	0	0	0	0	0.635445	0	0	1.906335	0	0	0	0	0.635445	1.906335	0	0.635445	0	0	0	0	0	0.635445	0	1.906335	0	0	0
6	0	0	0	1.906335	0.635445	58.46095	0	2.541781	4.448116	0.635445	1.27089	0	10.80257	0	0	15.25068	0	0	1.27089	0	0	8.896232	0.635445	69.89896	3.177226	0	0.635445	1.27089	5.719006	0.635445	0	36.22037	0	0	0.635445
5	0	0	0	1.906335	0	7.625342	0	0	1.906335	0	2.541781	0	1.27089	1.906335	0	2.541781	0	0	0	0	0	0	0	6.989896	0	0	0	0	0	0	0	2.541781	0	0	0.635445
4	6.354451	0	0	21.60513	0	35.58493	0	0.635445	2.541781	0	1.906335	0	5.719006	0	0	15.88613	0	0	0.635445	0	0	4.448116	0.635445	5.719006	1.27089	0.635445	0.635445	4.448116	1.27089	0.635445	0	43.84571	0	1.906335	0
3	0	0	0	1.27089	0	0.635445	0	0	0	0	0	0	0	0	0	0	0	0	0	0	0	0	0	0	0	0	0	0	0	0	0	0	0	0	0
2	0	0	0	3.177226	0	6.989896	0	0.635445	0	0	0	0	1.906335	0	0	4.448116	0.635445	0	0	0	0	0.635445	0	1.27089	0	0	0	0	1.27089	0	0.635445	10.80257	0	0	0
1	0	0	0	6.989896	0	32.4077	0	2.541781	1.906335	0	1.906335	0	0	0	0	19.06335	0	0	0	0	0.635445	11.43801	1.27089	5.083561	3.812671	0	0	0	1.906335	1.906335	0	35.58493	0.635445	1.27089	0
	1	2	3	4	5	6	7	8	9	10	11	12	13	14	15	16	17	18	19	20	21	22	23	24	25	26	27	28	29	30	31	32	33	34	35

Table S19. Frequency of the different dual-sgRNA cassettes of HK-L2 library in dCas9-containing *Vibrio* FA2 cultivated with 5 mg/mL of ampicillin (experiment, 1-Ex-3).

35	0	0	0	0	0	0	0	0	0	0	0	0	0	0	0	0	0	0	0	0	0	0	0	0	0	0	0	0	0	0	0	0	0	0	0
34	4.63392	6.95088	0	47.49768	1.15848	702.0389	0	5.7924	42.86376	1.15848	13.90176	5.7924	45.18072	2.31696	1.15848	130.9082	1.15848	0	12.74328	1.15848	5.7924	192.3077	1.15848	84.56905	12.74328	0	2.31696	2.31696	16.21872	10.42632	6.95088	392.7247	0	8.109361	3.47544
33	4.63392	0	0	12.74328	2.31696	67.19184	1.15848	2.31696	9.267841	0	12.74328	0	33.59592	0	0	48.65616	0	0	0	0	0	6.95088	0	44.02224	9.267841	2.31696	2.31696	0	1.15848	1.15848	3.47544	110.0556	4.63392	1.15848	0
32	0	0	0	20.85264	1.15848	528.2669	0	4.63392	26.64504	0	8.109361	0	79.93513	0	0	54.44856	0	0	1.15848	1.15848	2.31696	27.80352	1.15848	72.98424	5.7924	0	1.15848	1.15848	6.95088	1.15848	2.31696	61.39944	0	3.47544	0
31	0	0	0	0	0	1.15848	0	0	0	0	0	0	4.63392	0	0	1.15848	0	0	0	0	0	2.31696	0	1.15848	0	0	0	1.15848	0	0	0	3.47544	0	3.47544	0
30	0	0	0	0	0	3.47544	0	1.15848	0	0	0	2.31696	2.31696	0	0	3.47544	0	0	0	0	0	0	0	10.42632	1.15848	0	0	0	1.15848	1.15848	0	5.7924	0	0	0
29	4.63392	4.63392	0	38.22984	3.47544	698.5635	1.15848	4.63392	37.07136	11.5848	16.21872	2.31696	74.14272	0	0	203.8925	2.31696	2.31696	6.95088	0	3.47544	41.70528	1.15848	154.0778	9.267841	4.63392	5.7924	2.31696	10.42632	8.109361	10.42632	597.7757	4.63392	8.109361	1.15848
28	0	0	0	1.15848	0	8.109361	0	0	1.15848	0	0	0	2.31696	0	0	0	0	0	0	0	0	0	0	1.15848	0	0	0	0	0	0	0	5.7924	0	0	0
27	1.15848	0	0	6.95088	0	134.3837	0	1.15848	12.74328	1.15848	1.15848	0	17.3772	0	0	50.97312	2.31696	0	0	1.15848	1.15848	13.90176	0	50.97312	3.47544	2.31696	0	0	3.47544	3.47544	0	466.8675	0	1.15848	0
26	0	0	0	1.15848	0	24.32808	0	0	2.31696	1.15848	0	0	9.267841	0	0	10.42632	0	0	0	0	0	4.63392	0	4.63392	0	0	1.15848	0	0	1.15848	1.15848	12.74328	0	0	0
25	0	0	0	10.42632	0	18.53568	0	0	1.15848	0	0	0	4.63392	0	0	13.90176	0	0	0	0	0	3.47544	0	57.924	0	1.15848	0	0	1.15848	1.15848	0	15.06024	0	0	0
24	1.15848	0	0	15.06024	0	81.09361	0	0	16.21872	0	3.47544	0	55.60704	0	0	24.32808	0	0	1.15848	0	0	11.5848	1.15848	45.18072	2.31696	1.15848	1.15848	2.31696	3.47544	3.47544	1.15848	195.7831	0	5.7924	1.15848
23	0	0	0	3.47544	0	3.47544	0	2.31696	0	0	0	0	1.15848	0	0	0	0	0	0	0	0	0	0	1.15848	1.15848	0	1.15848	0	0	0	0	6.95088	0	0	0
22	1.15848	0	0	6.95088	0	45.18072	0	1.15848	4.63392	0	0	0	15.06024	0	0	28.962	0	0	1.15848	0	0	4.63392	0	30.12048	0	0	0	0	1.15848	1.15848	1.15848	64.87488	0	1.15848	0
21	0	0	0	0	0	4.63392	0	0	0	0	1.15848	0	0	0	0	3.47544	0	0	0	0	0	0	0	0	0	0	0	0	0	0	0	3.47544	0	0	0
20	0	0	0	0	0	3.47544	0	0	0	0	0	0	1.15848	0	0	2.31696	1.15848	0	1.15848	0	0	0	0	1.15848	0	0	0	0	0	0	0	3.47544	0	0	0
19	0	0	0	1.15848	0	4.63392	0	0	0	0	1.15848	0	0	1.15848	0	4.63392	0	0	0	0	0	0	0	0	0	0	0	0	0	0	0	4.63392	0	0	0
18	1.15848	0	0	0	0	3.47544	0	1.15848	0	0	0	0	0	0	0	3.47544	0	0	0	0	0	0	0	0	0	0	0	0	0	0	0	2.31696	0	0	0
17	0	0	0	0	0	0	0	0	0	0	0	0	0	0	0	0	0	0	0	0	0	0	0	0	0	0	0	0	0	0	0	0	0	0	0
16	5.7924	0	0	23.1696	0	626.7377	0	0	157.5533	1.15848	10.42632	1.15848	50.97312	0	0	40.5468	0	0	4.63392	2.31696	3.47544	35.91288	0	209.6849	16.21872	1.15848	3.47544	5.7924	10.42632	3.47544	3.47544	434.43	2.31696	5.7924	3.47544
15	0	0	0	0	0	0	0	0	0	0	0	0	0	0	0	0	0	0	0	0	0	0	0	0	0	0	0	0	0	0	0	0	0	0	0
14	0	0	0	0	0	3.47544	0	0	0	0	0	0	0	0	0	1.15848	0	0	0	0	0	0	0	1.15848	0	0	0	0	0	0	0	0	0	0	0
13	0	0	0	12.74328	0	171.4551	0	10.42632	12.74328	4.63392	25.48656	10.42632	9.267841	0	0	108.8971	0	0	1.15848	0	0	24.32808	2.31696	34.7544	31.27896	0	5.7924	1.15848	1.15848	4.63392	8.109361	207.3679	0	1.15848	4.63392
12	0	0	0	0	0	3.47544	0	0	0	0	0	0	0	0	0	2.31696	0	0	0	0	0	0	0	3.47544	0	0	0	0	0	0	0	1.15848	0	0	0
11	1.15848	0	0	4.63392	0	23.1696	0	0	4.63392	1.15848	0	0	1.15848	0	0	12.74328	0	0	0	0	1.15848	12.74328	0	16.21872	1.15848	0	1.15848	0	0	1.15848	0	28.962	0	5.7924	0
10	0	0	0	2.31696	0	9.267841	0	0	0	0	2.31696	0	0	0	0	1.15848	0	0	0	0	0	0	0	0	0	0	0	0	0	0	0	5.7924	0	1.15848	0
9	0	0	0	4.63392	0	19.69416	0	1.15848	0	1.15848	2.31696	0	1.15848	0	0	22.01112	0	0	15.06024	0	1.15848	11.5848	0	8.109361	0	0	0	0	2.31696	0	1.15848	26.64504	0	0	0
8	0	0	0	2.31696	0	4.63392	0	0	1.15848	0	0	0	0	0	0	1.15848	0	0	0	0	0	1.15848	0	5.7924	0	0	0	0	0	1.15848	0	10.42632	0	0	0
7	0	0	0	1.15848	0	1.15848	0	0	0	0	0	0	1.15848	0	0	0	0	0	0	0	0	0	0	0	0	0	0	0	0	0	0	0	0	0	0
6	0	0	0	6.95088	0	49.81464	0	2.31696	5.7924	0	1.15848	1.15848	11.5848	0	0	18.53568	1.15848	0	1.15848	0	1.15848	5.7924	0	91.51993	3.47544	0	0	1.15848	3.47544	3.47544	0	40.5468	0	0	0
5	0	0	0	0	0	5.7924	0	0	0	0	0	0	0	0	0	3.47544	0	0	0	0	0	1.15848	0	1.15848	0	0	0	0	0	0	0	2.31696	0	0	0
4	2.31696	0	0	3.47544	0	8.109361	0	0	4.63392	0	0	0	6.95088	0	0	6.95088	0	0	1.15848	0	0	2.31696	0	5.7924	1.15848	0	0	0	0	1.15848	0	28.962	0	0	0
3	0	0	0	0	0	0	0	0	0	0	0	0	0	0	0	0	0	0	0	0	0	0	0	0	0	0	0	0	0	0	0	0	0	0	0
2	0	0	0	1.15848	0	3.47544	0	0	1.15848	0	0	0	1.15848	0	0	1.15848	0	0	0	0	0	0	0	1.15848	0	0	0	0	0	0	0	15.06024	0	0	0
1	3.47544	0	0	4.63392	0	41.70528	0	0	2.31696	0	0	0	0	0	0	9.267841	0	0	0	0	0	13.90176	0	6.95088	1.15848	0	0	1.15848	0	5.7924	1.15848	39.38832	0	0	0
	1	2	3	4	5	6	7	8	9	10	11	12	13	14	15	16	17	18	19	20	21	22	23	24	25	26	27	28	29	30	31	32	33	34	35

Table S20. Frequency of the dual-sgRNA cassettes of HK-L2 library in dCas9-containing *Vibrio* FA2 cultivated without ampicillin (control, 2-CT-1).

35	0	0	0	0	0	0	0	0	0	0	0	0	0	0	0	0	0	0	0	0	0	0.448471	0	0	0	0	0	0	0	0	0	0	0	0	0
34	3.139295	0	0	129.608	0.448471	2294.376	0.448471	0.448471	107.1845	0	6.727061	0	1.793883	0.896941	0.448471	20.18118	1.345412	0	50.22872	0	0.896941	197.7756	0.896941	149.7892	9.417885	0.448471	0.448471	1.345412	3.587766	4.036236	3.139295	150.2377	0	15.248	0.448471
33	0	0	0	2.242354	0	5.381649	0	0	0.896941	0	0	0	2.242354	0	0	1.793883	0	0	0	0	0	0.896941	0	7.175531	0.896941	0	0	0.448471	0	0	0	4.933178	0	0	0
32	0.448471	0	0	21.52659	0	850.3005	0	0.448471	35.87766	0.448471	1.793883	0	13.00565	0	0	28.70213	0	0	2.242354	0	0	8.520944	0	52.91954	3.587766	0	0	0	0.448471	1.793883	0.896941	17.04189	0	8.520944	0
31	0	0	0	0	0	0.896941	0	0	0	0	0	0	0	0	0	0.448471	0	0	0	0	0	0	0	0.896941	0	0	0	0.448471	0	0	0	0	0	0.896941	0
30	0.448471	0	0	11.66024	0	100.009	0	0	23.76895	0	0.448471	0	0.896941	0	0	4.036236	0	0	1.793883	0	0	3.587766	0.448471	596.4661	4.036236	0	0	0.448471	0.448471	0.448471	0	32.73836	0	6.727061	0
29	0.896941	0	0	37.67154	0.896941	552.0675	0	1.345412	27.80518	0.448471	2.242354	0	0.896941	0	0	17.49036	0.448471	0	3.587766	0	0.448471	6.27859	0.448471	44.84707	3.587766	0	1.345412	0.448471	0.896941	3.139295	0.896941	171.7643	0	3.587766	0
28	0	0	0	0.448471	0	44.3986	0	0	0	0	0	0	0	0	0	0.448471	0	0	0	0	0	0.896941	0	4.933178	0	0	0.448471	0	0	0.448471	0	3.587766	0	0.448471	0
27	0.448471	0	0	13.90259	0	129.1596	0	0	37.22307	0	1.345412	0	3.139295	0	0	14.35106	0	0	2.690824	0	0	5.381649	0	42.60472	3.587766	0.448471	0.448471	0.448471	0.448471	2.690824	0.448471	622.9258	0.448471	3.139295	0
26	0	0	0	0	0	2.242354	0	0	0	0.448471	0	0	0.448471	0	0	0.448471	0	0	0	0	0	0	0	0.448471	0	0	0	0	0	0	0	0	0	0	0
25	0	0	0	5.381649	0	16.59342	0	0	3.139295	0	0	0	0.448471	0	0	4.036236	0	0	0.448471	0	0	0.896941	0	102.6998	0	0	0	0	0.448471	0.448471	0.448471	7.175531	0.448471	1.345412	0
24	0.448471	0	0	2.242354	0	15.248	0	0	3.587766	0.448471	0.448471	0	3.587766	0	0	1.345412	0	0	0	0	0.448471	2.690824	0	7.175531	4.036236	0	0	0.448471	0	0.448471	0	10.31483	0	0.448471	0
23	0	0	0	1.345412	0	1.793883	0	0.448471	0	0	0	0	0	0	0	0.896941	0	0	0	0	0	0.896941	0	0	0	0	0	0.448471	0	0	0	0.896941	0	0	0
22	0.448471	0.448471	0	4.484707	0.448471	48.88331	0	0.896941	11.66024	0.448471	0.896941	0.448471	1.345412	0.448471	0.448471	25.11436	0	0.448471	0.896941	0	0	0.448471	0.448471	30.04754	0.448471	0.448471	0	0.448471	0.896941	0	0	14.79953	0	1.345412	0.448471
21	0	0	0	0.448471	0	0.448471	0	0	0	0	0	0	0	0	0	0	0	0	0	0	0	0	0	0	0	0	0	0	0	0	0	0	0	0	0
20	0	0	0	0.448471	0.448471	1.345412	0	0	0	0	0	0	0	0	0	0.896941	0	0	0	0	0	0.896941	0	0	0	0	0	0	0	0	0	0.896941	0	0	0
19	0	0	0	0	0	0.448471	0	0	0	0	0	0	0	0	0	0	0	0	0	0	0	0	0	0	0	0	0	0	0	0	0	0	0	0	0
18	0	0	0	0	0	0.448471	0	0	0	0	0	0	0	0	0	0.448471	0	0	0	0	0	0	0	0	0	0	0	0	0	0	0	0.448471	0	0	0
17	0	0	0	0	0	0	0	0	0	0	0	0	0	0	0	0	0	0	0	0	0	0	0	0.448471	0	0	0	0	0	0	0	0	0	0	0
16	1.793883	0	0	22.42354	0	909.9471	0	1.793883	189.7031	0	1.345412	0	5.830119	0.448471	0	4.933178	0	0	2.242354	0	0.448471	4.484707	0	65.47672	27.80518	0	0	4.484707	1.793883	3.139295	0	174.0066	0	8.520944	0.448471
15	0	0	0	0	0	0	0	0	0	0	0	0	0	0	0	0	0	0	0	0	0	0.448471	0	0	0	0	0	0	0	0	0	0	0	0	0
14	0	0	0	0	0	0.448471	0	0	0	0	0	0	0	0	0	0	0	0	0	0	0	0	0	3.587766	0	0	0	0	0	0	0	0	0	0.448471	0
13	0	0	0	8.520944	0	23.76895	0	0.896941	5.381649	0	1.345412	0	0	0	0	1.345412	0	0	0.448471	0	0	6.27859	0	10.31483	39.91389	0	0	0	0	29.59907	0	11.21177	0	2.242354	0.896941
12	0	0	0	0	0	0.448471	0	0	0	0	0	0	0	0	0	0.448471	0	0	0	0	0	0.896941	0	0	0	0	0	0	0	0	0	0.448471	0	0	0
11	0.448471	0	0	1.345412	0	3.587766	0	0	0	0	0	0	0	0	0	1.793883	0	0	0	0	0	1.345412	0	4.484707	0	0	0	0	0	5.381649	0	2.690824	0	1.793883	0
10	0	0	0	0	0	0.896941	0	0	0	0	0	0	0	0	0	0	0	0	0	0	0	0	0	0	0	0	0	0	0	0	0	0.448471	0	0	0
9	0	0	0	12.55718	0	13.00565	0	0	2.242354	0	0.448471	0	0.448471	0	0	26.0113	0.448471	0	6.727061	0	0	1.793883	0	7.624002	0	0	0.448471	0.896941	0	0	0	5.830119	0.448471	0	0
8	0	0	0	0	0	12.10871	0	0	0	0	0	0	0	0	0	0.448471	0	0	0	0	0	0	0	0.448471	0	0	0	0	0	0	0	1.345412	0	0	0
7	0	0	0	0.448471	0	0	0	0	0	0	0	0	0	0	0	0	0	0	0	0	0	0	0	0.448471	0	0	0	0	0	0	0	0	0	0	0
6	0.448471	0	0	14.79953	0	43.05319	0.448471	0	25.56283	0.448471	0	0	1.345412	0	0	3.587766	0	0	1.793883	0	0.448471	4.933178	0.448471	463.7187	2.242354	0.448471	0.448471	0.448471	0.896941	0.448471	0.448471	31.39295	0	5.830119	0.448471
5	0	0	0	0	0	1.345412	0	0	0.896941	0	0	0	0	0	0	0	0	0	0	0	0	0.896941	0	0.448471	0	0	0	0	0	0	0	0.896941	0	0	0
4	10.31483	0	0	26.0113	0	60.99202	0	0	17.04189	0	0.448471	0	0.448471	0	0	30.94448	0	0	0.896941	0	0	2.242354	0	25.11436	0.896941	0	0	0.448471	0	0.448471	0	239.9318	0	5.830119	0
3	0	0	0	0	0	0	0	0	0	0	0	0	0	0	0	0	0	0	0	0	0	0	0	0	0	0	0	0	0	0	0	0	0	0	0
2	0.448471	0	0	0	0	1.345412	0	0	0	0	0	0	0	0	0	6.727061	0	0	0	0	0	0	0	0.896941	0	0	0	0	0	0	0	1.345412	0	0.448471	0
1	0	0	0	1.345412	0	7.624002	0	0	2.690824	0	0	0	0	0.448471	0	0	0	0	0	0	0	3.139295	0	1.793883	0.448471	0	0	0	0	0	0	29.59907	0	0.896941	0
	1	2	3	4	5	6	7	8	9	10	11	12	13	14	15	16	17	18	19	20	21	22	23	24	25	26	27	28	29	30	31	32	33	34	35

Table S21. Frequency of the dual-sgRNA cassettes of HK-L2 library in dCas9-containing *Vibrio* FA2 cultivated without ampicillin (control, 2-CT-2).

35	0	0	0	0	0	0	0	0	0	0	0	0	0	0	0	0	0	0	0	0	0	0	0	0	0	0	0	0	0	0	0	0	0	0	0
34	2.137666	1.42511	0	52.01653	0	438.2215	0	4.987887	22.08921	2.850221	8.550663	1.42511	41.3282	0.712555	0	74.10574	0	0	4.987887	0	0.712555	134.6729	0	111.8712	3.562776	0.712555	2.137666	0	10.68833	3.562776	1.42511	426.108	0	4.275331	1.42511
33	0.712555	0	0	12.11344	0	44.89098	0	0.712555	4.987887	0	3.562776	0	25.65199	0	0	115.4339	0	0	0	0	0	4.987887	0	36.34032	0.712555	0.712555	0.712555	0	2.137666	0.712555	0	112.5837	0	0	0
32	0.712555	0	0	12.82599	0	281.4593	0	2.850221	6.412997	0.712555	2.137666	0	34.20265	0	0	12.82599	0.712555	0.712555	0	0	0	2.850221	0	55.57931	1.42511	0	0	0	0	1.42511	0	43.46587	0	0.712555	0
31	0	0	0	0.712555	0	2.137666	0	0	0	0	0	0	4.987887	0	0	0.712555	0	0	0	0	0	1.42511	0	0.712555	0	0.712555	0	0	0	0	0	4.987887	0	3.562776	0
30	0.712555	0.712555	0	4.275331	0	37.05287	0	0.712555	7.125552	0	1.42511	0	5.700442	0	0	4.987887	0	0	0	0	0	2.850221	0	323.5001	0.712555	0	0	0	2.137666	2.137666	0	69.11786	0	0	0
29	3.562776	0	0	32.06499	2.137666	225.88	0	2.137666	13.53855	0	2.850221	0	31.35243	0	0	71.25552	0	0	1.42511	0	2.137666	24.93943	0	106.1707	5.700442	0	0.712555	0.712555	2.137666	9.263218	3.562776	372.6664	2.137666	4.275331	0
28	0	0	0	0	0	0.712555	0	0	0	0	0	0	0	0	0	0	0	0	0	0	0	0	0	0.712555	0	0	0	0	0	0	0	0.712555	0	0	0
27	0.712555	0	0	29.92732	0.712555	256.5199	0	1.42511	42.04076	0.712555	6.412997	0	39.90309	0	0	90.49451	0	0.712555	0.712555	0	1.42511	27.78965	0	210.9163	2.850221	0.712555	1.42511	1.42511	11.40088	6.412997	2.137666	3382.5	1.42511	9.263218	0.712555
26	0	0	0	0.712555	0	6.412997	0	0	0	0	0	0	2.850221	0	0	2.850221	0	0	0	0	0	2.850221	0	2.137666	0.712555	0	0	0	0	0	0	3.562776	0	0	0
25	0	0	0	2.137666	0	9.263218	0	0	0.712555	0	0	0	0	0	0	2.850221	0	0	0	0	0	0.712555	0	22.80177	0	0	0	0	0	0.712555	0	6.412997	0	0	0
24	0	0	0	14.96366	0	57.71697	0	0.712555	6.412997	0	1.42511	0	58.42953	0	0	14.96366	0	0	0	0	0	3.562776	0	29.92732	0.712555	0	0	0	0	1.42511	0.712555	241.5562	0	1.42511	0
23	0	0	0	1.42511	0	0.712555	0	0	0	0	0	0	0	0	0	0	0	0	0	0	0	0	0	0	0	0	0	0	0	0	0	1.42511	0	0	0
22	0	0.712555	0	1.42511	0	19.23899	0	0.712555	2.137666	0	0.712555	0	4.275331	0	0	12.11344	0	0.712555	0.712555	0	0.712555	0	0	24.22688	1.42511	0.712555	0	0	0.712555	2.137666	0	79.80618	0	0	0
21	0	0	0	0.712555	0	0	0	0	0	0	0	0	0	0	0	0	0	0	0	0	0	0	0	0.712555	0	0	0	0	0	0	0	0.712555	0	0	0
20	0	0	0	0	0	0.712555	0	0	0	0	0	0	0	0	0	0	0	0	0	0	0	0	0	0.712555	0	0	0	0	0	0	0	0.712555	0	0	0
19	0	0	0	0.712555	0	0.712555	0	0	0	0	0	0	1.42511	0	0	1.42511	0	0	0	0	0	0.712555	0	2.137666	0	0	0	0	0	0	0	3.562776	0	0.712555	0
18	0	0	0	0	0	1.42511	0	0	0	0	0	0	0	0	0	0.712555	0	0	0	0	0	0	0	2.137666	0	0	0	0	0	0	0	2.137666	0	0	0
17	0	0	0	0	0	0	0	0	0	0	0	0	0	0	0	0	0	0	0	0	0	0	0	0	0	0	0	0	0	0	0	0	0	0	0
16	0	0	0	12.82599	0	122.5595	0	2.137666	29.21476	0	1.42511	0	41.3282	0	0	7.125552	0	0	0	0	0	12.82599	0	209.4912	0.712555	0	0	0	0	2.137666	0.712555	180.2765	1.42511	0.712555	0
15	0	0	0	0	0	0	0	0	0	0	0	0	0	0	0	0	0	0	0	0	0	0	0	0	0	0	0	0	0	0	0	0	0	0	0
14	0	0	0	0	0	0	0	0	0	0	0	0	0	0	0	0	0	0	0	0	0	0	0	0	0	0	0	0	0	0	0	0	0	0	0
13	0	0	0	5.700442	0	128.9725	0	4.987887	7.838107	0.712555	12.11344	0	4.987887	0.712555	0	54.86675	0	0	0.712555	0	1.42511	9.975773	0	33.4901	9.975773	0.712555	3.562776	0	1.42511	19.95155	1.42511	150.3492	0	1.42511	0.712555
12	0	0	0	0	0	0.712555	0	0	0	0	0	0	0	0	0	0	0	0	0	0	0	0	0	0	0	0	0	0	0	0	0	0	0	0	0
11	0	0	0	2.137666	0	12.82599	0	0	0.712555	0	0	0	0.712555	0	0	2.850221	0	0	0	0	0	2.137666	0	19.95155	0	0	0	0	0.712555	4.275331	0	17.10133	0	0	0
10	0	0	0	0	0	2.850221	0	0	0	0	0	0	0	0	0	0	0	0	0	0	0	0	0	0	0	0	0	0	0	0	0	3.562776	0	0.712555	0
9	0	0	0	5.700442	0	6.412997	0	0	0.712555	0	0	0	1.42511	0	0	9.975773	0	0	1.42511	0	0	3.562776	0	7.125552	0	0	0	0	0	0	0	19.23899	0	2.137666	0
8	0	0	0	0.712555	0	3.562776	0	0	0	0	0	0	0	0	0	0.712555	0	0	0	0	0	0	0	2.137666	0	0	0	0	0	0.712555	0	2.137666	0	0	0
7	0	0	0	0.712555	0	0	0	0	0	0	0	0	0	0	0	0	0	0	0	0	0	0	0	0	0	0	0	0	0	0	0	0	0	0	0
6	0	0	0	4.275331	0	16.38877	0	0	4.275331	0	0.712555	0	3.562776	0	0	9.263218	0	0	0.712555	0	0	2.850221	0	130.3976	1.42511	0	0	0	0	1.42511	0	57.71697	0.712555	1.42511	0
5	0	0	0	0	0	0.712555	0	0	0	0	0	0	0	0	0	0	0	0	0	0	0	0	0	1.42511	0	0	0	0	0	0	0	0.712555	0	0	0
4	0	0	0	1.42511	0	9.263218	0	0	0.712555	0	0.712555	0	1.42511	0	0	9.975773	0	0	0	0	0	0.712555	0	4.275331	0.712555	0	0	0	0	1.42511	0	60.56719	0	0	0
3	0	0	0	0	0	0	0	0	0	0	0	0	0	0	0	0	0	0	0	0	0	0	0	0	0	0	0	0	0	0	0	0	0	0	0
2	0	0	0	0.712555	0	5.700442	0	0	0	0	0	0	0.712555	0	0	0.712555	0	0	0	0	0	0.712555	0	0	0	0	0	0	0	0	0	12.82599	0	0	0
1	0	0	0	0.712555	0	9.975773	0	0.712555	0.712555	0	0	0	0	0	0	1.42511	0	0	0	0	0	2.137666	0	2.850221	0	0	0	0	0	0	0	9.975773	0	0	0
	1	2	3	4	5	6	7	8	9	10	11	12	13	14	15	16	17	18	19	20	21	22	23	24	25	26	27	28	29	30	31	32	33	34	35

Table S22. Frequency of the dual-sgRNA cassettes of HK-L2 library in dCas9-containing *Vibrio* FA2 cultivated without ampicillin (control, 2-CT-3).

35	0	0	0	0	0	0	0	0	0	0	0	0	0	0	0	0	0	0	0	0	0	0	0	0	0	0	0	0	0	0	0	0	0	0	0
34	2.300588	0.657311	0	124.2318	0	2265.094	0	1.643277	100.5686	0.328655	5.258488	0.328655	4.929832	1.643277	0	26.62109	0	0	50.28429	0	0.657311	143.2938	0.657311	142.9651	11.8316	0.328655	1.314622	2.300588	3.943866	6.901765	2.629244	125.2177	0	10.84563	1.971933
33	0	0	0	5.587143	0	5.915798	0	0.657311	1.314622	0	0.985966	0	1.971933	0.328655	0	1.971933	0	0	0	0	0.328655	0.985966	0	6.244454	1.314622	0	0.657311	0	0	0	0.328655	4.929832	0.657311	0	0
32	0.985966	0	0	20.37664	0	814.4083	0	0.657311	35.49479	0	1.314622	0	10.51698	0	0	24.97782	0	0	4.272521	0.328655	0	2.957899	0	46.34042	3.61521	0.328655	0.657311	0.328655	1.314622	1.314622	0.328655	15.44681	0	5.915798	0
31	0	0	0	0	0	0	0	0	0.657311	0	0	0	0	0	0	0	0	0	0	0	0	0	0	0	0	0.328655	0	0	0	0	0	0.328655	0	0.328655	0
30	0.657311	0	0	16.10412	0	96.29605	0	0	29.25034	0	0.657311	0	0.657311	0	0	3.286555	0	0	1.643277	0	0	2.300588	0.328655	648.4372	2.629244	0	0	1.314622	0.328655	0.985966	0	28.92168	0	6.244454	0
29	0.657311	0	0	33.1942	0.985966	576.133	0	0.985966	27.2784	0	3.286555	0.328655	3.61521	0	0.328655	14.46084	0	0	1.643277	0	0.657311	4.272521	0.657311	45.0258	3.61521	0	0.985966	0.328655	1.643277	0.657311	0.328655	141.6505	0	6.573109	0.328655
28	0	0	0	0.657311	0	42.39656	0	0	2.629244	0	0.328655	0	0.328655	0	0	0.328655	0	0	0	0	0	0	0	2.957899	0.328655	0	0	0.328655	0	0	0	5.587143	0	0.328655	0
27	0.985966	0	0	17.09008	0	133.7628	0	0	27.60706	0	1.314622	0	0.328655	0	0	14.46084	0	0	1.643277	0	0	4.929832	0	53.57084	2.957899	0	0.328655	0	0.328655	2.629244	0	689.8478	0	4.929832	0.657311
26	0	0	0	0.328655	0	1.314622	0	0	0	0.328655	0	0	0	0	0	0	0	0	0	0	0	0	0	0.985966	0	0	0	0	0	0	0	0.328655	0	0	0
25	0	0	0	6.244454	0	17.7474	0	0	4.601177	0	0	0	0	0.328655	0	1.643277	0	0	0.328655	0	0	0.985966	0	119.3019	0	0	0	0	0.328655	0.657311	0	4.929832	1.643277	1.971933	0
24	0	0	0	1.643277	0	14.13219	0	0	2.957899	0	0	0	3.61521	0	0	0.328655	0	0	0.328655	0.328655	0	0.657311	0	7.23042	2.629244	0	0.328655	0.328655	0	0.657311	0	14.13219	0	0.657311	0
23	0	0	0	0	0	1.971933	0	0.657311	0.328655	0	0	0.328655	0	0	0	2.300588	0	0	0	0	0	0.328655	0	0.328655	0	0	0	0	0	0	0	0.657311	0	0	0
22	0.328655	0.657311	0.328655	1.314622	0	21.36261	0	0	5.258488	0.657311	0	0.328655	0	0	0	26.29244	0	0	0.657311	0.657311	0	0.328655	0	15.77546	0.657311	0	0	0	0	0.328655	0	8.873698	0	0.657311	0
21	0	0	0	0	0	0.328655	0	0	0	0	0	0	0	0	0	0.328655	0	0	0	0	0	0.328655	0	0	0	0	0	0	0	0	0	0	0	0	0
20	0	0	0	1.314622	0.328655	0.328655	0	0	0.328655	0	0	0	0	0	0	0	0	0	0	0	0	0.328655	0	0	0	0	0	0	0	0	0	0.985966	0	0	0
19	0	0	0	0	0	1.314622	0	0	0	0	0	0	0	0.328655	0	0.328655	0	0	0	0	0	0	0	0	0.328655	0	0	0	0	0	0	0.328655	0	0	0
18	0	0	0	0	0	0.328655	0	0	0	0	0	0	0	0	0	0	0	0	0	0	0	0	0	0.328655	0	0	0	0	0	0	0	1.643277	0	0	0
17	0	0	0	0	0	0	0	0	0	0	0	0	0	0	0	0	0	0	0	0	0	0.328655	0	0.328655	0	0	0	0	0	0	0	0	0	0	0
16	1.314622	0	0	18.07605	0.328655	1009.301	0	0.657311	185.033	0	2.300588	0.657311	2.629244	0	0	7.559076	0.328655	0	4.601177	0	0.328655	6.573109	0	79.86328	31.55093	0	0.328655	7.559076	0.328655	3.286555	0.657311	184.0471	0.985966	8.545042	0.328655
15	0	0	0	0	0	0	0	0	0	0	0	0	0	0	0	0	0	0	0	0	0	0	0	0	0	0	0	0	0	0	0	0	0	0	0
14	0	0	0	0	0	0.657311	0	0	0.328655	0	0	0	0	0	0	0.985966	0	0	0	0	0	0	0	3.943866	0	0	0	0	0	0	0	0.657311	0	0	0
13	0	0	0	10.18832	0	22.01992	0.328655	0.328655	4.929832	0.657311	2.957899	0.657311	0	0	0	3.943866	0	0	0.657311	0	0	6.901765	0	8.545042	37.46672	0	0	0.657311	0.328655	33.52286	0	12.48891	0	0.657311	1.314622
12	0.328655	0	0	0	0	0.657311	0	0	0.328655	0	0	0	0	0	0	0	0	0	0	0	0	0	0	0	0	0	0	0	0	0	0	0	0	0.328655	0
11	0	0	0	1.314622	0	7.559076	0	0.328655	0.657311	0	0	0	0	0	0	1.971933	0	0	0	0	0	1.643277	0	3.61521	0.657311	0	0	0.328655	0	6.901765	0	2.629244	0	1.314622	0
10	0	0	0	0	0	0	0	0	0	0	0.328655	0	0	0	0	0	0	0	0	0	0	0	0	0	0	0	0	0	0	0	0	0.657311	0	0.328655	0
9	0	0	0	10.84563	0	16.10412	0	0.328655	1.643277	0	0.328655	0	0	0.657311	0	30.56496	0	0	9.202353	0	0	0.985966	0	6.244454	0.657311	0	0.328655	0	0	0	0	5.915798	0	0.328655	0
8	0	0	0	0.985966	0.328655	13.14622	0	0	0	0	0	0	0	0	0	0.328655	0	0	0	0	0	0.657311	0	0.328655	0	0	0	0	0	0	0	0.985966	0	0	0
7	0	0	0	0	0	0.657311	0	0	0	0	0	0	0	0	0	0.657311	0	0	0	0	0	0	0	0	0	0	0	0	0	0	0	0	0	0	0
6	0.328655	0	0	11.8316	0	42.0679	0.328655	0	18.40471	0	0	0	1.643277	0	0.328655	4.272521	0	0	1.643277	0	0	1.971933	0	482.7949	1.643277	0	0.328655	0	0	0.657311	0.328655	28.59303	0	3.286555	0
5	0	0	0	0.657311	0	1.314622	0	0	0.328655	0	0	0	0	0.328655	0	0.985966	0	0	0	0	0	0	0	0.985966	0	0	0	0	0	0	0	0	0	0	0
4	6.901765	0	0	28.59303	0	61.45857	0	0	15.11815	0	0	0	1.314622	0	0	33.1942	0	0	0.657311	0	0	2.300588	0	20.04798	0.985966	0	0.328655	0.657311	0	0.657311	0	212.9687	0	5.587143	0
3	0	0	0	0	0	0.328655	0	0	0	0	0	0	0	0	0	0	0	0	0	0	0	0	0	0	0	0	0	0	0	0	0	0	0	0	0
2	0.328655	0	0	0.657311	0	0.328655	0	0	0	0	0	0	0	0	0	4.272521	0	0	0	0	0	0	0	0.328655	0	0	0	0	0	0	0	2.300588	0	0	0
1	0	0	0	1.314622	0	5.587143	0	0	1.643277	0	0.328655	0.328655	0	0	0	1.314622	0	0	0	0	0	0.328655	0	1.314622	0	0	0	0	0	0.328655	0	31.22227	0	0.328655	0
	1	2	3	4	5	6	7	8	9	10	11	12	13	14	15	16	17	18	19	20	21	22	23	24	25	26	27	28	29	30	31	32	33	34	35

Table S23. Frequency of the different dual-sgRNA cassettes of HK-L2 library in dCas9-containing *Vibrio* FA2 cultivated with 5 mg/mL of ampicillin (experiment, 2-Ex-1).

35	0	0	0	0	0	0	0	0.382102	0	0	0	0	0	0	0	0	0	0	0	0	0	0	0	0	0	0	0	0	0	0	0	0	0	0	0
34	2.674716	6.49574	0	64.57529	1.910512	429.8651	0.382102	14.13779	18.72301	3.438921	32.4787	9.934661	45.08807	0	0.382102	90.94035	2.292614	0.764205	4.585228	0	5.349433	111.956	0	92.85087	12.60938	2.674716	3.056819	0	35.53552	3.438921	10.31676	262.8864	0.764205	6.113637	14.13779
33	1.528409	0	0	22.54404	0	157.4262	0	10.69887	8.406251	0.764205	16.4304	0.764205	41.26705	0	0	126.4759	0.382102	0.382102	0.382102	0	0.764205	14.13779	0	93.23297	33.625	1.528409	3.438921	1.146307	9.170456	0.382102	3.438921	102.4034	0.764205	0	0.764205
32	1.528409	0	0	32.0966	2.292614	377.8992	0	8.788354	3.438921	0.382102	14.51989	0.764205	116.5412	0	0	49.2912	0.382102	1.146307	1.528409	0.382102	2.292614	20.63353	0.382102	75.27416	7.259944	0.382102	2.674716	0	13.37358	0	2.292614	37.82813	0	4.585228	0.382102
31	0	0	0	0	0	6.877842	0	0	1.146307	0	1.146307	0	5.349433	0	0	0.764205	0	0.382102	0	0	0	0.382102	0	1.910512	0.764205	1.146307	0	0	0	0	0.382102	3.821023	0	2.674716	0
30	0	0.382102	0	0	0	0.764205	0	0	0	0	0	0	0	0	0	2.292614	0	0	0	0	0	0	0	0.764205	0	0	0	0	0	0	0	0.382102	0	0	0
29	7.642047	3.821023	0	113.4844	5.731535	990.4092	3.056819	24.45455	40.50285	11.46307	43.17756	3.438921	140.6137	0.382102	0	320.5839	0.764205	3.056819	4.585228	0.382102	7.259944	79.47728	1.910512	274.3495	29.03978	6.113637	9.934661	0	25.21875	0.382102	14.13779	620.5342	6.113637	21.77983	2.674716
28	0	0	0	0	0	0	0	0	0	0	0	0	0	0	0	0	0	0	0	0	0	0.764205	0	0	0	0	0	0	0	0	0	0	0	0	0
27	0.382102	0.764205	0	9.934661	0	84.06251	0	3.056819	3.056819	0.764205	3.056819	0.764205	12.99148	0	0	25.98296	0.382102	0	0	0	0	5.349433	0	37.06393	3.056819	0	0.764205	0	3.056819	0	0	120.3622	0	1.910512	1.146307
26	0.382102	0.382102	0	3.438921	0	33.625	0	3.821023	0.764205	0.382102	2.292614	0.382102	15.6662	0	0	8.024149	0	0	0	0	0	3.821023	0	7.642047	1.146307	0	0.382102	0	0.764205	0	0.764205	7.642047	0	0.764205	0
25	0	0	0	12.22727	0	56.55114	0	0	4.203126	0.764205	2.292614	1.146307	10.69887	0	0	16.8125	0	0.382102	0.764205	0	0.382102	4.203126	0.382102	60.37217	1.146307	1.528409	0.382102	0	2.292614	0	0.382102	18.72301	0	1.146307	0.382102
24	1.528409	0	0	35.15341	0.382102	188.3764	0.382102	5.349433	5.731535	0.764205	9.552558	0	71.07103	0	0.382102	34.38921	0	0.382102	0	0	0.764205	7.642047	0	75.65626	3.821023	0.382102	0.764205	0.382102	5.731535	0.382102	1.146307	163.5398	0.764205	2.292614	1.910512
23	0	0	0	0.764205	0	9.170456	0	0.764205	0.382102	0	0.382102	0	1.528409	0	0	2.292614	0	0	0	0	0	0.382102	0.382102	2.292614	0	0.382102	0	0	0.764205	0	0	7.642047	0	0	0
22	0	0.764205	0	3.438921	0.764205	41.64915	0	3.438921	4.203126	0	6.113637	0.382102	17.1946	0	0	16.8125	0	0.764205	3.056819	0	0.764205	6.49574	0	28.65767	1.910512	1.146307	0.764205	0	4.203126	0.382102	1.146307	46.23438	0.764205	2.674716	0.382102
21	0	0	0	0.382102	0	1.910512	0	0	0	0	0.764205	0	0	0	0	1.146307	0	0	0	0	0	0.382102	0	1.910512	0	0	0	0	0.382102	0	0	0.764205	0	0	0
20	0	0	0	0	0	1.910512	0	0	0	0	0	0	0.382102	0	0	0	0	0	0	0	0	0	0	1.146307	0	0	0	0	0	0	0	1.146307	0	0	0
19	0	0	0	1.528409	0.382102	4.585228	0	0	0.764205	0	0	0	3.438921	0	0	2.292614	0	0	0	0	0	3.438921	0	1.146307	0	0	0	0	0.382102	0	0.382102	3.821023	0	0.382102	0
18	0.382102	0	0	1.528409	0	7.642047	0	0.382102	1.146307	0	0.382102	0	1.528409	0	0	4.203126	0	0	0	0	0	0.764205	0	1.528409	0.382102	0.382102	0	0	0.382102	0	0	2.292614	0.382102	0	0
17	0	0	0	0	0	0.382102	0	0	0	0	0	0	0	0	0	0	0	0	0	0	0	0	0	0.382102	0	0	0	0	0	0	0	0.382102	0	0	0
16	1.910512	0.764205	0	18.72301	0.382102	169.2713	0.382102	5.731535	13.75568	0.382102	10.69887	0.764205	34.00711	0	0	14.90199	0	0	0.764205	0	2.674716	13.37358	0.382102	159.3367	9.934661	1.528409	1.910512	0	7.642047	0.382102	0.764205	123.8012	1.146307	3.438921	0.764205
15	0	0	0	0	0	0.382102	0	0	0	0	0	0	0	0	0	0.382102	0	0	0	0	0	0	0	0	0	0	0	0	0	0	0	0	0	0	0
14	0	0	0	0	0	0	0	0	0	0	0	0	0	0	0	0	0	0	0	0	0	0	0	0	0	0	0	0	0	0	0	0	0	0	0
13	1.528409	0.382102	0	27.89347	1.146307	437.5072	0.764205	26.74716	6.113637	5.731535	32.0966	8.406251	21.39773	0	0	122.6548	0.382102	1.528409	3.821023	0	2.292614	30.95029	0	73.74575	39.73864	1.528409	5.349433	0	12.22727	1.910512	7.642047	199.4574	0.764205	7.259944	6.113637
12	0	0	0	0.382102	0	3.821023	0	0	0	0	0	0	0.764205	0	0	3.438921	0	0	0	0	0	1.146307	0	7.642047	0	0	0	0	0	0	0	6.113637	0	0	0
11	1.910512	0.382102	0	25.98296	0	102.4034	0	1.528409	6.113637	2.674716	0.382102	0.382102	9.552558	0	0	31.71449	0	0	0.382102	0	0	16.8125	0	61.13637	5.349433	0	0.764205	0	5.731535	0	0.382102	63.04688	0	6.49574	0.382102
10	0	0	0	1.910512	0	4.96733	0	0	0.764205	0	2.674716	0.382102	0.764205	0	0	1.146307	0	0	0	0	0	1.146307	0	3.056819	0.764205	0	0	0	1.146307	0	0	1.910512	0	1.528409	0
9	0	0	0	8.406251	0	24.83665	0.382102	1.146307	0.382102	0.764205	5.731535	0.382102	4.96733	0	0	5.349433	0	0	3.056819	0.382102	0	12.22727	0	9.170456	1.528409	0	0	0	1.528409	0	0.764205	28.65767	0	0	0
8	0	0	0	3.821023	0	18.34091	0	0	0	0	0.764205	0	2.292614	0	0	3.438921	0	0	0	0	0.382102	1.910512	0	14.90199	0	0.382102	0.382102	0	0.764205	0	0	19.86932	0	0	0
7	0	0	0	1.910512	0	2.292614	0	0	0	0	0	0	0	0	0	1.146307	0	0	0	0	0	1.146307	0	0.382102	0.382102	0.382102	0	0	0	0	0	1.528409	0.764205	0	0
6	1.146307	0	0	8.406251	0.382102	136.7926	0	4.585228	4.585228	0.764205	6.113637	0.764205	32.0966	0	0	23.30824	0	0.764205	0.382102	0	0	4.585228	0	61.90058	3.438921	0.382102	0.764205	0.382102	6.113637	0	0	50.0554	0.382102	1.528409	0.764205
5	0	0	0	0.382102	0	7.642047	0	0	0.382102	0	2.292614	0	1.146307	0.382102	0	4.203126	0	0	0	0	0	0.382102	0	9.170456	0.764205	0	0	0	0	0	0	1.146307	0	0	0
4	0.382102	0	0	2.674716	0	28.65767	0	1.528409	0.764205	0	1.910512	0	6.877842	0	0	13.37358	0	0	0	0	0.764205	1.528409	0	10.69887	1.146307	0	0.382102	0.382102	1.528409	0	0	23.69034	0	1.528409	0.382102
3	0	0	0	0	0	0	0	0	0	0	0	0	0	0	0	0	0	0	0	0	0	0	0	0	0	0	0	0	0	0	0	0	0	0	0
2	0	0	0	3.821023	0	6.49574	0	0.382102	0.764205	0	0	0	4.203126	0	0	0.382102	0	0.764205	0	0	0	0.764205	0	1.146307	0	0	0	0	0.382102	0	0.382102	17.1946	0	0	0
1	1.146307	0	0	4.203126	0	63.04688	0	3.821023	1.528409	0	4.585228	0	3.438921	0	0	8.788354	0	0	0.764205	0	0.764205	8.024149	0	14.51989	0.382102	0	0.382102	0	0.382102	0	0	30.18608	0.382102	0.382102	0.764205
	1	2	3	4	5	6	7	8	9	10	11	12	13	14	15	16	17	18	19	20	21	22	23	24	25	26	27	28	29	30	31	32	33	34	35

Table S24. Frequency of the different dual-sgRNA cassettes of HK-L2 library in dCas9-containing *Vibrio* FA2 cultivated with 5 mg/mL of ampicillin (experiment, 2-Ex-2).

35	0	0	0	0	0	0.926827	0	0.463414	0	0	0	0	0	0	0	0	0	0	0	0	0	0	0	0	0	0	0	0	0	0	0	0	0	0	0
34	2.317068	1.390241	0	14.82923	0.926827	735.4372	0.463414	2.780481	19.46337	0.463414	31.0487	0.926827	12.04875	0	0.926827	37.99991	1.390241	0	21.78043	0	0.463414	149.6826	0	35.21943	17.60971	2.780481	2.317068	0.463414	12.04875	0	6.024376	372.121	0	3.707308	2.317068
33	0.463414	0	0	2.780481	0.463414	49.12183	0	1.390241	1.853654	0	8.341443	0	14.36582	0	0	10.1951	0	0	0	0	0	4.634135	0	16.68289	12.97558	1.390241	0.463414	0	2.317068	0	0.463414	75.5364	1.390241	0.463414	0
32	3.707308	0.463414	0	12.51216	0	1793.874	0	4.634135	25.02433	0.926827	20.39019	0	33.36577	0	0	25.95116	0.463414	0.463414	3.707308	0.463414	3.707308	26.41457	0	57.92669	13.43899	0.926827	0.463414	0	4.170722	0.463414	0.463414	106.1217	0.926827	5.560962	0.463414
31	0	0	0	0	0	3.707308	0	0	0	0	1.853654	0	4.170722	0	0	0	0	0	0	0	0	0	0	1.390241	0	0.926827	0	0	0	0	0	3.707308	0	2.780481	0
30	0	0	0	0	0	0.926827	0	0	0	0	0	0	0	0	0	0	0	0	0	0	0	0.463414	0	0	0	0	0	0	0	0	0	0.926827	0	0	0
29	7.414616	3.243895	0	18.07313	1.390241	899.949	1.853654	8.341443	30.12188	3.243895	37.07308	0	37.99991	0	0	66.26813	0	0	2.317068	0.463414	5.560962	31.51212	0	85.26808	16.21947	2.317068	0.926827	0	4.170722	0	12.97558	1143.705	4.170722	5.560962	0.463414
28	0	0	0	0	0	0	0	0	0	0	0	0	0	0	0	0	0	0	0	0	0	0	0	0	0	0	0	0	0	0	0	0	0	0	0
27	1.390241	0	0	5.097549	0	261.3652	0.463414	0.926827	28.73164	0	11.58534	0.463414	6.024376	0	0	18.99995	0.926827	0.463414	2.780481	0.463414	0.926827	11.58534	0	25.95116	11.12192	1.853654	0.463414	0.463414	4.634135	0	1.390241	1526.021	0	4.634135	1.390241
26	0	0	0	0	0	11.12192	0	0	0	0	1.390241	0	0.926827	0	0	0.926827	0	0	0	0	0	2.317068	0	0	2.780481	0	0	0	0	0	0.463414	4.634135	0	0	0.463414
25	0	0	0	0.926827	0	36.60967	0	0	2.317068	0	0.926827	0	1.390241	0	0	5.097549	0	0	0	0	0	1.853654	0	50.51207	0.926827	0	0	0	0.926827	0	0	19.46337	0	0.463414	0
24	0.463414	0	0	2.780481	0	60.24376	0	0.463414	3.243895	0	3.243895	0	6.951203	0	0	5.560962	0	0	0.926827	0	0.463414	3.243895	0	10.1951	3.243895	0	0	0	0.463414	0	0.463414	90.36563	0.463414	1.390241	0
23	0	0	0	0.926827	0	0.463414	0	0.926827	0	0	0	0	0	0	0	2.780481	0	0	0	0	0	0.463414	0	0.463414	0	0	0	0	0	0	0	2.317068	0	0	0
22	1.853654	0	0	0.926827	0.463414	50.04866	0.463414	2.317068	3.707308	0	1.853654	0.926827	2.780481	0	0.463414	22.70726	0.463414	0	0.926827	0	0	3.243895	1.390241	7.87803	0.463414	1.390241	0.926827	0	0.926827	0	0.463414	71.36568	0	1.390241	0.463414
21	0	0	0	0	0	3.243895	0	0	0	0	0	0	0	0	0	1.390241	0	0	0	0	0	1.390241	0	1.853654	0.463414	0	0	0	0	0	0	2.317068	0	0	0
20	0	0	0	0	0	0.926827	0	0	0	0	0	0	0	0	0	0.926827	0	0	0	0	0	0.926827	0	0	0	0	0	0	0	0	0	7.414616	0	0	0
19	0	0	0	0.463414	0	1.390241	0	0	0	0	0	0	0	0	0	0	0	0	0.463414	0	0	0	0	0.463414	0	0	0	0	0.463414	0	0	2.780481	0	0	0
18	0	0	0	0.463414	0	3.243895	0	0	0	0	0.463414	0	0	0	0	0	0	0	0	0	0	0.926827	0	0.463414	0	0	0	0	0.463414	0	0	4.170722	0	0	0
17	0	0	0	0	0	0	0	0	0	0	0	0	0	0	0	0	0	0	0	0	0	0.463414	0	0	0	0	0	0	0	0	0	0.463414	0	0	0
16	1.390241	0	0	1.390241	0	151.5362	0	1.390241	60.24376	0	3.707308	0	1.853654	0	0	1.390241	0.463414	0	0.463414	0	0.926827	4.170722	0	17.60971	11.58534	0	0.463414	0	1.390241	0	0.463414	119.5607	0	2.317068	0.463414
15	0	0	0	0	0	0	0	0	0	0	0	0	0	0	0	0	0	0	0	0	0	0.926827	0	0	0	0	0	0	0	0	0	0	0	0	0
14	0	0	0	0	0	0.463414	0	0	0	0	0	0	0	0	0	0	0	0	0	0	0	0	0	0	0	0	0	0	0	0	0	0.463414	0	0	0
13	0.463414	0	0	1.853654	0.463414	74.60957	0	5.097549	4.634135	0.926827	11.58534	0.463414	0.463414	0	0	5.097549	0	0	1.853654	0	0.926827	4.170722	0	5.097549	89.43881	0	0.463414	0	0.926827	0	1.390241	54.21938	0	0.926827	0.926827
12	0	0	0	0	0	0	0	0	0	0	0	0	0	0	0	0.463414	0	0	0	0	0	0	0	0.926827	0	0	0	0	0	0	0	0	0	0	0
11	0.926827	0	0	3.243895	0	41.2438	0	0.463414	2.317068	1.853654	0.926827	0	0.926827	0	0	12.04875	0	0	0	0	0	13.90241	0	22.70726	5.560962	0.926827	0.463414	0	0.463414	0.463414	0	29.19505	0	2.317068	0
10	0	0	0	0.463414	0	2.780481	0	0	0	0	2.780481	0	0	0	0	0	0	0	0	0	0	0	0	0.926827	0	0	0	0	0	0	0	0	0	0	0
9	0	0	0	6.024376	0	9.26827	0	0.463414	0.463414	0	1.853654	0	0.463414	0	0	7.87803	0	0	31.0487	0	0.463414	4.634135	0.463414	1.390241	0.463414	0	0	0	0.926827	0	0	15.75606	0	0.463414	0
8	0	0	0	0	0.463414	37.07308	0	0	0.463414	0.463414	0	0	0	0	0	0.463414	0	0	0	0	0	0.463414	0	1.853654	0	0	0	0	0	0	0	8.341443	0	0	0
7	0	0	0	0.463414	0	0.463414	0	0.463414	0	0	0.463414	0	0	0	0	0	0	0	0	0	0	0.926827	0	0	0	0	0	0	0	0	0	2.317068	0	0	0
6	0.926827	0	0	0	0	50.51207	0	1.390241	1.390241	0.463414	1.390241	0	2.780481	0	0	3.707308	0	0	0.463414	0	0.463414	2.317068	0.463414	71.82909	0.463414	0.463414	0.463414	0	0.926827	0	0	42.17063	0	0.463414	0
5	0	0	0	0	0	2.317068	0	0	0.463414	0	1.390241	0	0	0	0	0.463414	0	0	0	0	0	0	0	2.317068	0.463414	0	0	0	0	0	0	0.926827	0	0	0
4	0	0	0	1.390241	0	5.560962	0	0	0	0	1.853654	0	0	0	0	1.853654	0	0	0	0	0	0	0	0.463414	0.463414	0	0	0	0	0	0	16.68289	0	0	0
3	0	0	0	0	0	0	0	0	0	0	0	0	0	0	0	0	0	0	0	0	0	0.463414	0	0	0	0	0	0	0	0	0	0.463414	0	0	0
2	0	0	0	0	0	3.243895	0	0	0	0	0	0	0	0	0	0.463414	0	0	0.463414	0	0	1.390241	0	0	0	0	0	0	0.463414	0	0	6.024376	0	0	0
1	0.463414	0	0	0	0	14.36582	0	0.926827	0.463414	0	1.390241	0	0.463414	0	0	0	0	0	0	0	0	2.780481	0	2.317068	0.926827	0	0	0	0	0	0	16.68289	0	0	0
	1	2	3	4	5	6	7	8	9	10	11	12	13	14	15	16	17	18	19	20	21	22	23	24	25	26	27	28	29	30	31	32	33	34	35

Table S25. Frequency of the different dual-sgRNA cassettes of HK-L2 library in dCas9-containing *Vibrio* FA2 cultivated with 5 mg/mL of ampicillin (experiment, 2-Ex-3).

35	0	0	0	0	0	0	0	0	0	0	0	0	0	0	0	0	0	0	0	0	0	0	0	0	0	0	0	0	0	0	0	0	0	0	0
34	4.153563	3.560197	0	16.02089	0.593366	943.4522	0	7.120394	51.02949	0.593366	32.04177	2.373465	17.20762	0.593366	0	63.49018	1.780098	0	11.86732	0.593366	3.560197	187.5037	0	109.1794	11.86732	1.780098	5.340295	0	30.26167	0.593366	4.153563	351.2728	2.966831	1.780098	3.560197
33	1.780098	0	0	2.373465	1.186732	131.1339	0	4.746929	4.153563	0.593366	17.80098	0	14.24079	0	0	24.92138	0	0	0	0	0	8.900492	0	49.24939	24.32801	0.593366	0	0	5.933662	0	1.186732	101.4656	1.186732	0.593366	0.593366
32	1.780098	0	0	8.900492	0	1015.843	0.593366	5.933662	29.07494	1.186732	13.64742	0	42.129	0	0	43.9091	0	0	0.593366	0	1.186732	41.53563	1.186732	134.1008	4.746929	0.593366	2.373465	0	10.08722	0	0	55.77642	0.593366	2.966831	0
31	0	0	0	0	0	5.933662	0	0	0	0	0.593366	0	4.153563	0	0	0	0	0	0.593366	0	0	0.593366	0	2.373465	0	0.593366	0	0	0	0	0	4.153563	0	2.373465	0
30	0	0	0	0.593366	0	0.593366	0	0	0	0	0.593366	0	0	0	0	0.593366	0	0	0	0	0	0	0	0	0	0	0	0	0	0	0	0	0	0	0
29	5.340295	2.966831	0	18.98772	2.373465	1055.005	0.593366	4.746929	53.99632	5.340295	39.16217	0	56.36979	0.593366	0	131.7273	0	0.593366	3.560197	0.593366	7.71376	59.92998	0	193.4374	16.02089	2.373465	2.966831	0	10.68059	0	7.71376	677.6242	3.560197	9.493859	3.560197
28	0	0	0	0	0	0.593366	0	0	0	0	0	0	0	0	0	0	0	0	0	0	0	0	0	0	0	0	0	0	0	0	0	0.593366	0	0	0
27	1.780098	0	0	0.593366	0	98.49878	0	2.373465	8.307126	0	7.120394	0	3.560197	0	0	17.80098	0	0	0.593366	0	0.593366	7.120394	0	23.73465	2.373465	0	0	0	2.373465	0	0	388.0615	0.593366	2.373465	0
26	0	0	0	0	0	7.120394	0	0	0.593366	0	0	0.593366	0	0	0	1.780098	0	0	0	0	0.593366	1.186732	0	2.966831	1.780098	0	0	0	0	0	0	1.780098	0	0	0
25	0	0	0	3.560197	0.593366	60.52335	0	0	2.966831	0.593366	1.186732	0	2.966831	0	0	6.527028	0	0	0	0	0	4.153563	0	64.08355	0.593366	1.780098	0	0	0	0	1.780098	19.58108	0	1.186732	0
24	2.966831	0	0	4.746929	0.593366	182.7568	0	2.373465	12.46069	0.593366	5.933662	0	17.20762	0	0	16.02089	0	0	0.593366	0	1.186732	7.71376	0	35.60197	6.527028	1.780098	4.153563	0	2.966831	1.186732	0.593366	183.9435	0	3.560197	1.780098
23	0	0	0	0.593366	0	2.966831	0	0	0.593366	0	0.593366	0	0	0	0	2.966831	0	0	0	0	0	0	0	1.186732	0	0	0	0	0	0	0	0.593366	0	0	0
22	1.186732	0	0	0	0.593366	58.14988	0	2.966831	5.933662	0	2.966831	0.593366	3.560197	0	0	26.70148	0.593366	0.593366	2.373465	0	1.186732	7.120394	0	55.18305	1.780098	1.186732	1.186732	0	1.780098	0	1.186732	54.58969	0	1.186732	0
21	0	0	0	0	0	9.493859	0	0	0	0	0	0	0	0	0	0.593366	0	0	0	0	0	1.186732	0	4.746929	0	0	0	0	0	0	0	0	0	0	0
20	0	0	0	0	0	0.593366	0	0	0	0	0	0	0	0	0	0	0	0	0	0	0	0.593366	0	0	0	0	0	0	0	0	0	2.966831	0	0	0
19	0.593366	0	0	0	0	1.780098	0	0	0	0	0	0	0	0	0	0	0	0	0	0	0	1.186732	0	1.780098	0	0	0	0	0	0	0	3.560197	0	0	0
18	0	0	0	0	0	11.27396	0	0	0	0	0.593366	0	0	0	0	1.186732	0	0	0	0	0	4.153563	0	0	0	0	0	0	2.373465	0	0	6.527028	0	0	0
17	0	0	0	0	0	0	0	0	0	0	0	0	0	0	0	0	0	0	0	0	0	0	0	0	0	0	0	0	0	0	0	0	0	0	0
16	4.746929	0.593366	0	4.153563	0	623.0345	0	2.966831	112.1462	0.593366	18.39435	0.593366	10.68059	0.593366	0	11.27396	0	1.780098	1.186732	0	4.153563	23.14128	0	113.9263	22.54791	0	3.560197	0	10.68059	0	0	226.6659	1.780098	6.527028	1.186732
15	0	0	0	0	0	0	0	0	0	0	0	0	0	0	0	0	0	0	0	0	0	0	0	0	0	0	0	0	0	0	0	0	0	0	0
14	0	0	0	0	0	0	0	0	0	0	0	0	0	0	0	0	0	0	0	0	0	0	0	0	0	0	0	0	0	0	0	1.186732	0	0	0
13	0	0	0	1.780098	0	107.9926	0	7.71376	5.933662	2.373465	17.20762	1.186732	1.186732	0	0	18.98772	0	0	0.593366	0	1.780098	13.05406	0	18.39435	30.26167	0	3.560197	0	2.373465	0.593366	3.560197	71.79731	0	2.373465	1.186732
12	0	0	0	0	0	1.186732	0	0	0	0	0	0	0	0	0	0	0	0	0	0	0	0	0	1.780098	0	0	0	0	0	0	0	0.593366	0	0	0
11	1.780098	0.593366	0	2.966831	0	47.46929	0	0.593366	4.746929	0	0	0	1.186732	0	0	21.95455	0	0	0	0	1.186732	8.900492	0	49.84276	2.373465	0.593366	0.593366	0	1.186732	0	0	24.32801	0	6.527028	0
10	0	0	0	0	0	4.153563	0	0	1.186732	0	6.527028	0	0	0	0	0.593366	0	0	0	0	0	0	0	1.186732	0.593366	0	0	0	0	0	0	1.186732	0	0	0
9	0	0	0	1.186732	0	21.36118	0	0	1.186732	0	5.340295	0	0	0	0	11.86732	0	0	8.307126	0	1.186732	11.86732	0	7.71376	1.186732	0	0	0	2.373465	0	0.593366	13.64742	0	0.593366	0
8	0	0	0	0	0	19.58108	0	0	0	0	1.186732	0	0.593366	0	0	0.593366	0	0	0	0	0	1.186732	0	6.527028	0.593366	0	0	0	0	0	0	11.27396	0	0	0
7	0	0	0	0.593366	0	0	0	0	0.593366	0	0	0	1.186732	0	0	1.780098	0	0	0	0	0	1.780098	0	0.593366	0	0	0	0	0.593366	0	0	1.780098	0	0	0.593366
6	1.780098	0	0	1.186732	0	83.07126	0	1.780098	8.307126	0.593366	3.560197	0	5.340295	0	0	7.71376	0.593366	0	0	0.593366	0.593366	2.373465	0	193.4374	0.593366	0.593366	0	0	2.373465	0	0.593366	56.96315	0	0	1.780098
5	0	0	0	0	0	2.373465	0	0	0.593366	0	0.593366	0	0	0	0	1.780098	0	0	0	0	0	0	0	3.560197	1.780098	0	0	0	0	0	0	0.593366	0	0	0
4	0	0	0	0	0	1.186732	0	0	0.593366	0	0	0	0	0	0	1.186732	0	0	0	0	0	0	0	0.593366	0	0	0	0	0	0	0	8.307126	0	0	0
3	0	0	0	0	0	0	0	0	0	0	0	0	0	0	0	0	0	0	0	0	0	0	0	0	0	0	0	0	0	0	0	0	0	0	0
2	0	0	0	0	0	1.780098	0	0.593366	0	0	0	0	0	0	0	0	0	0	0	0	0	1.186732	0	2.966831	0.593366	0.593366	0	0	0.593366	0	0	8.900492	0	0	0
1	0	0	0	1.186732	0.593366	35.0086	0	1.780098	1.186732	0	1.780098	0	1.780098	0	0	3.560197	0	0	0	0	0	7.120394	0	5.340295	0.593366	0	0	0	0	0	0	27.29484	0	0.593366	0
	1	2	3	4	5	6	7	8	9	10	11	12	13	14	15	16	17	18	19	20	21	22	23	24	25	26	27	28	29	30	31	32	33	34	35

Table S26. Mapping ratios of the numbers of reads and dual-sgRNA cassettes based on the NGS data calculation and statistics.

Group name	Mapping ration (%)
HK-L1	90.2 %
HK-L2	85.3 %
HK-S	80.5 %
Gly-L1	92.1 %
Gly-L2	83.5 %
Gly-S	81.6 %

Table S27. Abbreviation and corresponding full name used in this study.

Abbreviation	Full name
MRP	metabolic reprogramming
SMRP	smart metabolic reprogramming
CPA	cellular perception apparatus
HK	histidine kinase
CDCK	CRISPRi-mediated dual-gene combinational knockdown strategy
SMIA	survivorship-based metabolic interaction analysis
T	template DNA strand
NT	non-template DNA strand
FACS	fluorescence-activated cell sorting
NGS	next-generation sequencing
HTS	high-throughput screening

Table S28. Bioinformatics designs for several microbial cells.

Organism	Sensitive Nodes Count	Histidine Kinase Count	Regulator Count	Primers Design
Bacillus licheniformis ATCC 14580	291	23	320	☑
Bacillus licheniformis ATCC 14581	323	31	292	☑
Bacillus subtilis 168	279	22	248	☑
Escherichia coli K12 (MG1655)	236	11	209	☑
Escherichia coli BL21 (DE3)	230	16	209	☑
Klebsiella pneumoniae SGH10	430	25	397	☑
Pseudomonas aeruginosa PAO1	512	44	456	☑
Synechococcus sp. PCC7002	64	11	55	☑
Vibrio natriegens ATCC 14048	195	30	166	☑
Vibrio alginolyticus FA2	366	35	320	☑
Synechococcus sp. PCC11901	75	12	65	☑
Pseudomonas putida S16	458	51	412	☑
